# Phosphates as Assisting Groups in Glycan Synthesis

**DOI:** 10.1021/acscentsci.3c00896

**Published:** 2023-12-20

**Authors:** Eric T. Sletten, Giulio Fittolani, Nives Hribernik, Marlene C. S. Dal Colle, Peter H. Seeberger, Martina Delbianco

**Affiliations:** † Department of Biomolecular Systems, Max Planck Institute of Colloids and Interfaces, Am Mühlenberg 1, 14476 Potsdam, Germany; ‡ Department of Chemistry and Biochemistry, Freie Universität Berlin, Arnimallee 22, 14195 Berlin, Germany

## Abstract

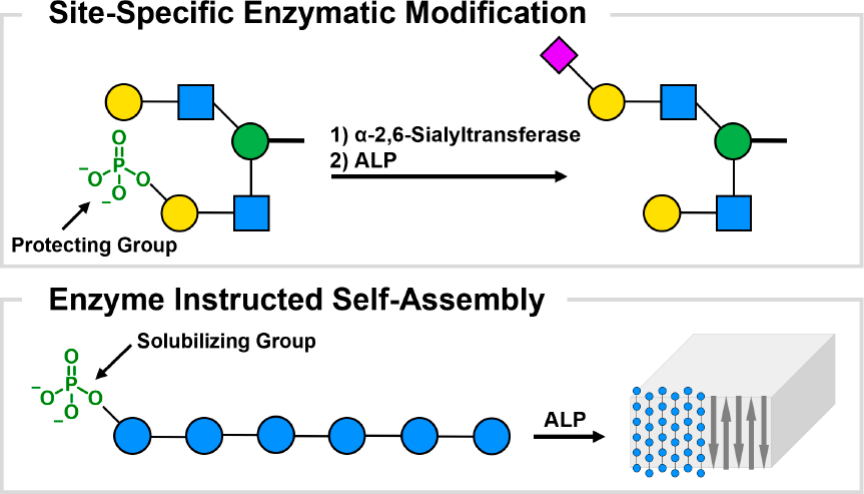

In nature, phosphates are added to and cleaved from molecules to direct biological pathways. The concept was adapted to overcome limitations in the chemical synthesis of complex oligosaccharides. Phosphates were chemically placed on synthetic glycans to ensure site-specific enzymatic elongation by sialylation. In addition, the deliberate placement of phosphates helped to solubilize and isolate aggregating glycans. Upon traceless removal of the phosphates by enzymatic treatment with alkaline phosphatase, the native glycan structure was revealed, and the assembly of glycan nanostructures was triggered.

## Abstract 2

Phosphate esters are presented as traceless assisting groups to enable site-specific enzymatic modification of glycans and to trigger the assembly of glycan nanostructures.

## Introduction

Phosphate esters are ubiquitous in nature as they underpin many biological functions.^1−3^ Negatively charged phosphate esters can be added by kinases and cleaved by phosphatases to tune the biological properties of molecules to regulate binding, compartmentalization, and enzymatic activity.^1−3^ Inspired by nature, phosphate groups have been exploited in the synthesis of peptides and nucleic acids.^4−6^ Phosphate groups have been installed on a synthetic peptide to shield specific serine residues from enzymatic ribosylation,^7^ to prevent aggregation during the synthesis of self-assembling peptides, or to trigger the formation of peptide-based supramolecular assemblies.^8−11^ In glycan synthesis, the potential of phosphate esters remains mostly unexplored, with phosphotriesters serving as anomeric leaving groups for chemical glycosylations^12,13^ or in deoxygenation reactions.^14^

Automated glycan assembly (AGA) has emerged as a versatile synthetic platform to rapidly obtain well-defined glycan oligomers.^15^ Following many synthetic and technological advances,^16^ some challenges such as the installation of certain monosaccharides (e.g., α-sialic acid)^15,17^ and linkages (e.g., β-mannosides) remain.^18,19^ While enzymatic installation of challenging monosaccharide residues on glycan backbones prepared by AGA could address some of these limitations, its scope remained bound to simple backbones lacking degenerative residues.^20^ An additional challenge often faced during post-AGA deprotection steps^21^ is the formation of ill-defined aggregates that dramatically decrease overall yields for glycans that are prone to aggregation.^22^

**Figure 1:**
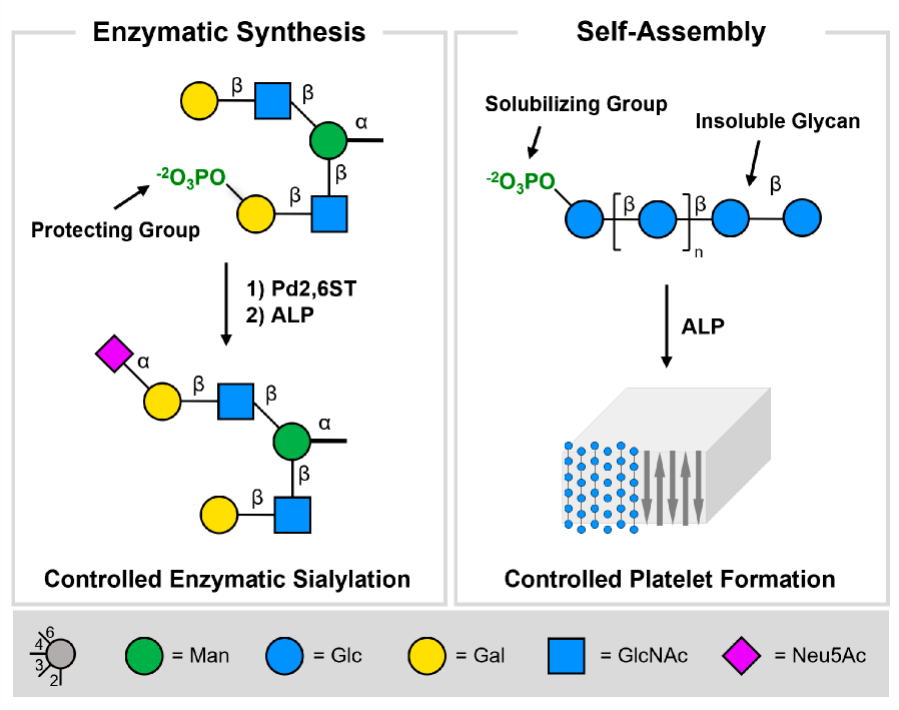
Phosphate monoesters are regarded as assisting groups in glycan synthesis to ensure the site-specific enzymatic sialylation of degenerate galactose residues (left) and as solubilizing groups for the procurement of cellulose chains (right). Traceless removal of the phosphate by ALP reveals the natural glycan and triggers the controlled formation of cellulose platelets.

We hypothesized that phosphate monoesters may serve as cleavable, ionic functional groups to mask specific glycan residues during enzymatic manipulations and prevent premature glycan aggregation by increasing the solubility of the oligomer in the reaction media (Figure 1). The phosphate group can be chemically inserted at a specific position during the AGA process,^23^ is stable during all post-AGA manipulation steps such as protecting group removal,^23^ and can be cleaved enzymatically by alkaline phosphatase (ALP) to reveal the natural structure in a traceless fashion at the end of the synthesis.^7,10^ Here, we demonstrate the utility of phosphates as assisting groups for the synthesis of a complex asymmetrically sialylated N-glycan as well as in the isolation and controlled assembly of cellulose oligosaccharides.

## Results and Discussion

### Site-Specific Enzymatic Modification

Sialic acid is a 3-deoxy-2-ketoaldonic acid commonly present on the nonreducing terminus of glycoproteins and glycolipids, where it is essential for mediating molecular recognition events at the basis of biological processes including infection and immune response.^24,25^ Controlling the reactivity and stereoselectivity of sialic acid building blocks in glycosylations is challenging because the tertiary C-2 anomeric center is sterically hindered and the lack of a hydroxyl group in the C-3 position prevents the use of a neighboring participating group. Enzymatic methods using sialyltransferases, rather than chemical glycosylations, have been utilized to install the desired sialic acid moieties onto a synthetic galactose-containing glycan backbone with regio- and stereoselectivity.^20^ However, in the presence of multiple galactose residues, the sialylation of several residues led to diminished yields and challenging purifications.^20,26,27^

Protecting groups on a synthetic backbone can ensure site-specific enzymatic modifications.^25,28−35^ Azides or *N*-trifluoroacetyl moieties can mask amines, and *O*-acetyl, *O*-methyl, and *O*-tetrahydropyranyl (THP) conceal hydroxyl groups.^28−31,33,36^ Enzymatic installation of fucosyl, sialyl, or C-6 oxidized galactosyl residues can help to direct further enzymatic functionalization.^25,34,35^ While powerful, these blocking groups require specific enzymes,^34^ complicate chemical synthesis for installation and removal (i.e., *O*-acetyls),^33^ or cannot be removed (i.e., *O*-methyl).^31^ Phosphorylation could offer a general and mild alternative to the site-specific enzymatic functionalization of complex glycans. Phosphates can be easily added to a wide variety of glycans at the positions that require protection.^23^ At the end of the synthesis, the native glycan can be quickly revealed by phosphate ester cleavage by treatment with ALP.^7^

We tested the viability of the phosphate blocking approach for the asymmetric sialylation of a diantennary N-glycan pentasaccharide (**8**, Figure 1) containing two degenerate galactose units. The commercially available sialyltransferase (Pd2,6ST) from a bacterial source (*Photobacterium damselae*) was tested for the installation of a α-2,6-sialic acid on a phosphorylated glycan.^25,37^ Bacterial transferases, in contrast to mammalian glycosyl transferases, are readily expressed and isolated and are not inhibited by negatively charged glycans, such as phosphorylated nucleotide byproducts or sulfated glycans.^38−41^ Indeed, a phosphorylated LacNAc disaccharide was sialylated with Pd2,6ST without inhibition of the bacterial transferase (see the SI).

**Figure 2:**
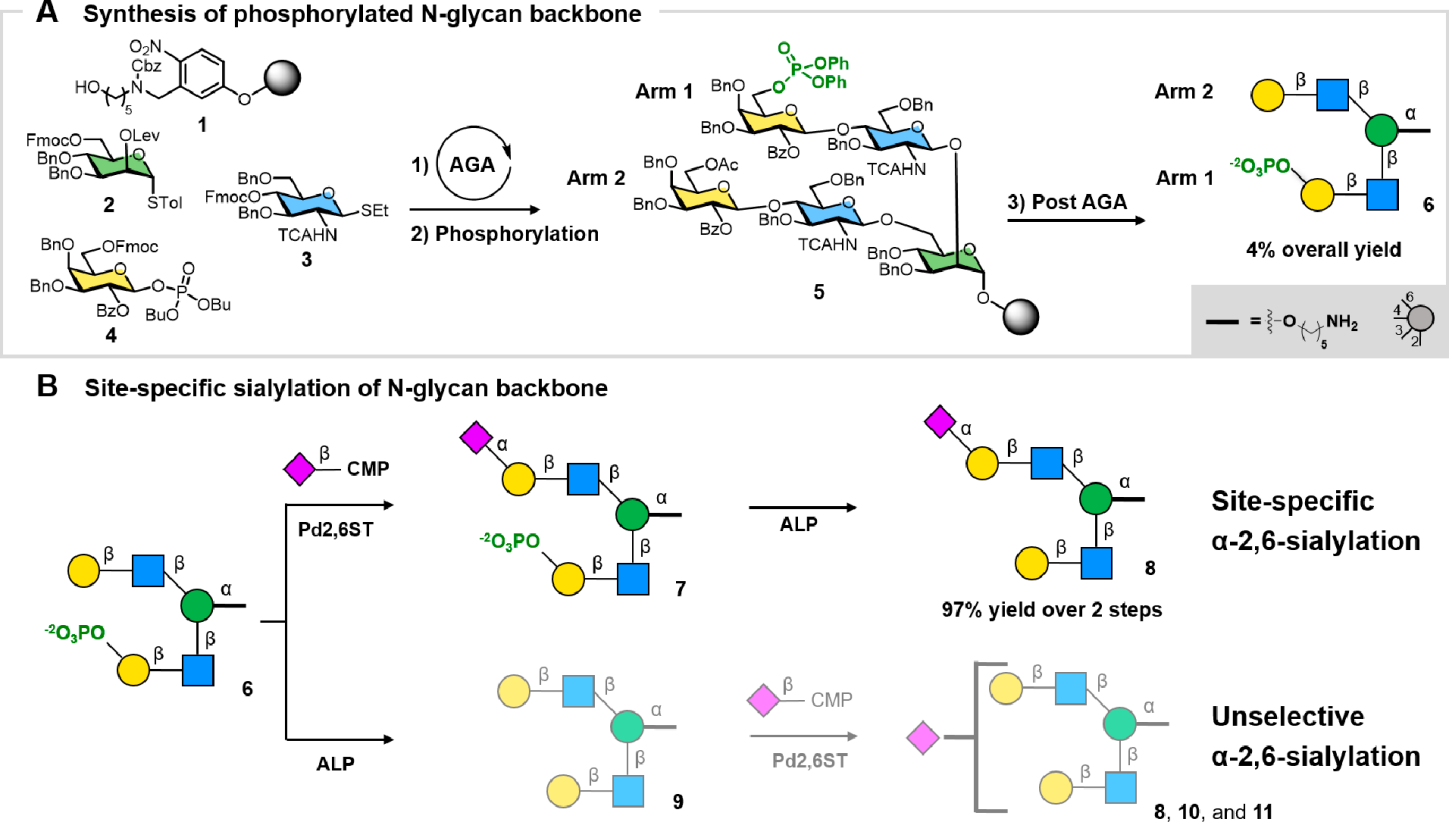
Site-specific sialylation of phosphorylated glycans. (A) Synthesis of monophosphorylated pentasaccharide **6** (see Figure S3a). Post-AGA step including photocleavage, hydrogenolysis, dephenylation, hydrolysis, and purification. (B) Site-specific sialylation of **6** by Pd2,6ST and CMP-sialic acid to construct phosphorylated hexasaccharide **7**, followed by ALP-mediated dephosphorylation to afford monosialylated hexasaccharide **8**. The dephosphorylation prior to sialylation furnished a mixture of sialylated products (see Figures S5 and S6 for HPLC traces).

AGA of a branched N-glycan backbone on photolabile solid support **1** employed building blocks **2**, **3**, and **4** (Figure 2A; see SI for synthetic details). Cycles of glycosylation, deprotection, and capping allowed us to construct the solid-bound pentasaccharide, exposing a free hydroxyl group at the C-6 position of the galactose residue on one of the branches (Arm 1, Figure S3). On-resin phosphorylation gave monophosphorylated compound **5** (Figure 2A). Cleavage from the resin and global deprotection afforded monophosphorylated compound **6** in 4% yield over 16 steps. For enzymatic sialylation (Figure 2B, top), oligosaccharide **6** was incubated with Pd2,6ST and CMP-sialic acid to furnish intermediate **7**. Upon complete sialylation, the sialyltransferase was denatured at 90 °C before ALP was added to cleave the phosphate monoester. Hexasaccharide **8** was isolated in 97% yield (over two steps). Double sialylation was not observed even when excess donor and sialyltransferase were used. When ALP-mediated dephosphorylation preceded sialylation, a mixture of sialylated oligosaccharides was observed (**8**, **10**, and **11**; Figure 2B, bottom), confirming the utility of the phosphate-assisted approach. These results demonstrate the potential of phosphomonoesters as general and mild blocking groups toward the residue-specific enzymatic functionalization of complex glycans.

### Enzyme-Instructed Self-Assembly

Aggregation of partially protected intermediates or hydrophobic, crystalline glycans drastically reduces the yield of chemical glycan synthesis.^22^ This problem is exacerbated by the increasing length of the glycan structures.^42^ Severe aggregation in many common solvents has limited the synthesis of cellulose oligomers (i.e., β-1,4-oligoglucosides) exceeding hexamers in length.^22^ Incorporating chemical modifications such as methylation into the cellulose chain generates “defects” that prevent premature aggregation and grant access to longer oligomers in good yields.^21,43^ These permanent modifications, albeit minimal, affect the aggregation of the resulting structures and make a comparison to natural glycans impossible.^44^ We envisioned that the insertion of an ionic phosphate monoester will prevent undesired aggregation during and after deprotection by increasing oligomer solubility in aqueous media.^8^ Enzymatic dephosphorylation will then afford the native cellulose chains, triggering the formation of well-defined cellulose materials as ideal substrates for studying natural cellulose assemblies.^45^

**Figure 3:**
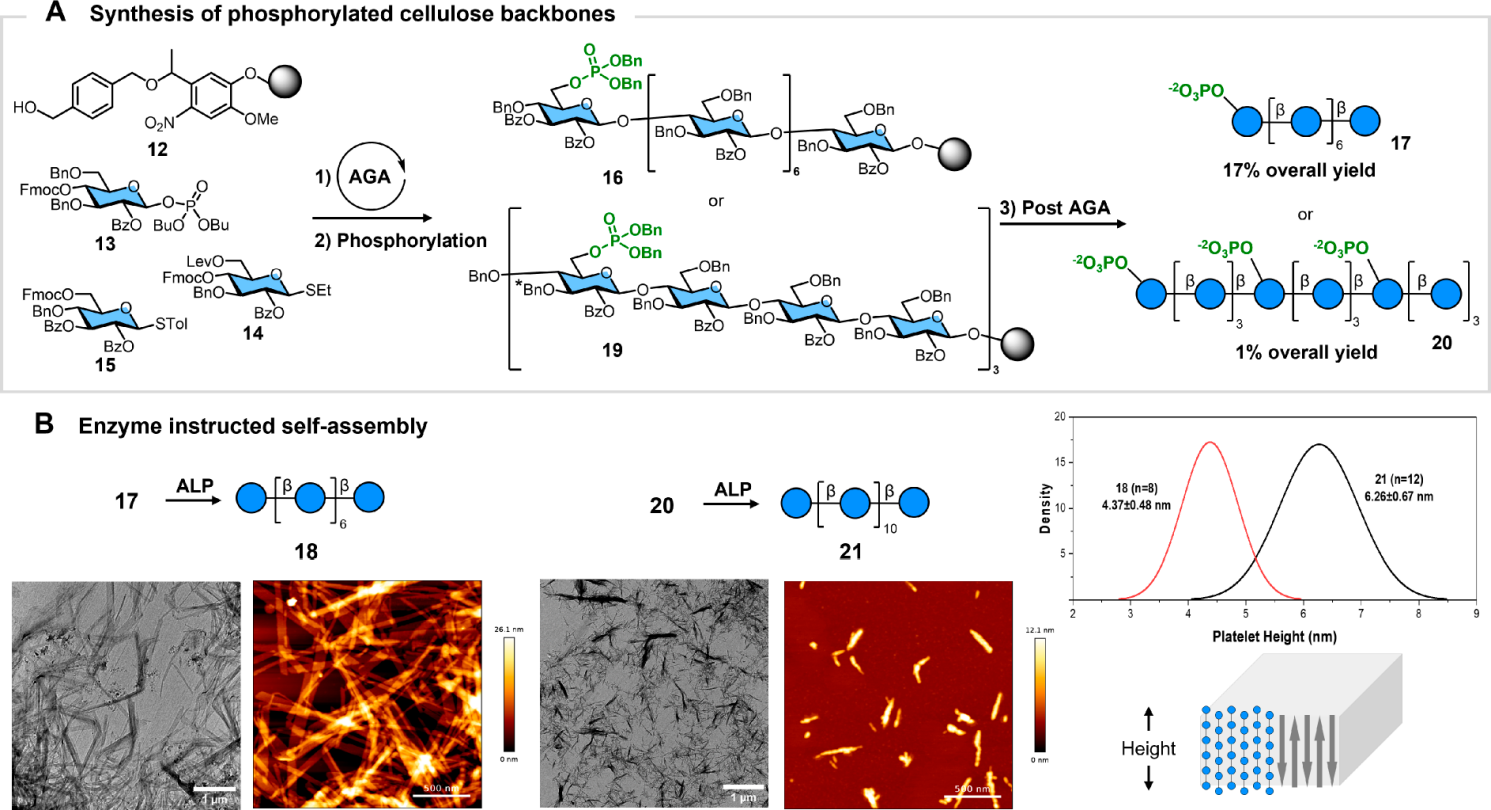
Enzyme-instructed self-assembly of cellulose oligosaccharides. (A) Synthesis of phosphorylated cellulose oligomers (see Figure S3b,c). Post-AGA steps include hydrolysis, photocleavage, hydrogenolysis, and purification. (B) ALP-mediated dephosphorylation of oligosaccharides **17** and **20** triggered the assembly of cellulose platelets with defined height, as shown by TEM and AFM analysis. *C-3 protection on the terminal unit is BzO.

Cellulose octasaccharide **18** was chosen as the first target (Figure 3A) as it was isolated previously only in trace amounts due to its poor solubility in the reaction media.^22^ Phosphorylated octasaccharide **17** (see Figure S3b for synthetic details) proved more soluble (>100 mg/mL) and was isolated in 17% yield over 20 steps. Treatment with ALP afforded the insoluble oligosaccharide **18** that was easily isolated by centrifugation (Figure 3B, 36% yield). A similar approach permitted the synthesis of longer glycan chains such as dodecasaccharide **20** in 1% yield over 29 steps (Figure 3A and Figure S3c). Here, three phosphate groups were added to ensure solubility throughout the synthetic process. Dephosphorylation proceeded smoothly for the terminal phosphate unit, while liberating the internal residues required longer times (Figure S18). The desired 12mer **21** was obtained after incubation with ALP for 72 h (Figure 3B).

TEM and AFM analyses revealed that the resulting precipitates consist of long crystallites much like what had been found for shorter oligomers (Figure 3B).^22^ Upon dephosphorylation, the cellulose oligomers aligned in an antiparallel manner along the platelet thickness. AFM analysis confirmed the formation of platelets with a well-defined height correlating with the oligomer length (Figure 3B). When ALP-triggered dephosphorylation was performed at different temperatures, concentrations, and enzyme amounts, minimal changes in crystallite morphology were observed (see the SI). These results suggest that dephosphorylation by ALP can be used to trigger the assembly of glycans into nanomaterials of defined dimensions as models to understand the natural aggregation of polysaccharides^22^ or for *in situ* biomedical applications.^8,10,11^

## Conclusions

We demonstrated that the incorporation of a phosphate monoester on a glycan backbone enabled the site-specific enzymatic functionalization of an asymmetric N-glycan. Moreover, the ionic nature of the phosphate monoesters was exploited to prevent the uncontrolled aggregation of long, self-assembling cellulose oligomers and facilitate isolation in aqueous media. Chemical phosphorylation on the solid support was reversed by enzymatic dephosphorylation to release the natural glycan and/or trigger the formation of glycan nanomaterials. As in biological processes, the phosphate monoester is a traceless assisting group that is ideally suited to obtain well-defined oligosaccharides and precision self-assembled glycan materials as probes for studying natural glycans.^22^

## Supporting Information Available

Experimental procedures and characterization data for all new compounds and enzymatic reactions (PDF)
Transparent Peer Review report available (PDF)
