# Supplementary material for: Phosphates as Assisting Groups in Glycan Synthesis
Source: ACS Cent Sci. 2023 Dec 20;10(1):138–42. doi: 10.1021/acscentsci.3c00896 (PMC10823511; doi:10.1021/acscentsci.3c00896)
Supplement: Supplementary file 1 — oc3c00896_si_001.pdf [file oc3c00896_si_001.pdf]

Phosphates as assisting groups in glycan synthesis

Eric T. Sletten‡[1], Giulio Fittolani‡[1], Nives Hribernik[1], Marlene C. S. Dal Colle[1,2], Peter H. Seeberger[1,2]*, Martina Delbianco[1]*

[1]*Department of Biomolecular Systems, Max Planck Institute of Colloids and Interfaces, Am Mühlenberg 1, 14476 Potsdam (Germany)*

[2]*Department of Chemistry and Biochemistry, Freie Universität Berlin, Arnimallee 22, 14195 Berlin (Germany)*

‡ *These authors contributed equally*

e-mail: martina.delbianco@mpikg.mpg.de (M.D.) and peter.seeberger@mpikg.mpg.de (P.H.S.)

Supporting Information

Table of Contents

1 General materials and methods S4
2 Building blocks for AGA S5
3 Automated Glycan Assembly S6
3.1 General materials and method S6
3.2 Preparation of stock solutions S6
3.3 Modules for automated synthesis S7
3.4 Post-synthesizer manipulations (Post-AGA) S12
4 Oligosaccharides syntheses S15
4.1 Synthesis of **S4** S16
4.2 Synthesis of **8** S22
4.3 Synthesis of **S5** S28
4.4 Synthesis of **17** S33
4.5 Synthesis of **20** S39
5 Regioselective enzymatic sialylation of N-glycan fragment S46
5.1 Disaccharide phosphate screening S46
5.2 Selective sialylation S47
5.3 Nonselective sialylation S51
6 ALP mediated precipitation of well-defined cellulose chains S53
6.1 Experimental S53
6.1.1 General protocol S53
6.1.2 TEM imaging S53
6.1.3 AFM imaging S53
6.2 Monophosphate octamer S54
6.2.1 Screening the conditional effects on platelet morphology S54
6.2.2 Large scale dephosphorylation S54
6.2.3 Imaging of phosphated octamer **17** -before ALP S56
6.2.4 Imaging comparison S57
6.2.5 Platelet height analysis S59
6.3 Diphosphate hexamer screening S66

6.4 Triphosphate dodecamer S68
6.5 Platelet height comparison data S73
7 References S76

1 General materials and methods

All chemicals used were reagent grade and used as supplied unless otherwise noted. The automated syntheses were performed on a home-built synthesizer developed at the Max Planck Institute of Colloids and Interfaces. Analytical thin-layer chromatography (TLC) was performed on Merck silica gel 60 F254 plates (0.25 mm). Compounds were visualized by UV irradiation or dipping the plate in a staining solution (sugar stain: 10% H_2SO_4 in EtOH; CAM: 48 g/L ammonium molybdate, 60 g/L ceric ammonium molybdate in 6% H_2SO_4 aqueous solution). Flash column chromatography was carried out by using forced flow of the indicated solvent on Fluka Kieselgel 60 M (0.04 – 0.063 mm). Analysis and purification by normal and reverse phase HPLC were performed using the Agilent 1260 series equipped with a multiple wavelength detector (MWD) and an evaporative light scattering detector (ELSD) or a Knauer Azura with a MWD. Products were lyophilized using a Christ Alpha 2-4 LD plus freeze dryer. ^{1}H, ^{13}C, ^{31}P, and HSQC NMR spectra were recorded on a Bruker 400-MR (400 MHz), a Varian 600-MR (600 MHz), or a Bruker Biospin AVANCE700 (700 MHz) spectrometer. Spectra were recorded in $CDCl_3$ by using the solvent residual peak chemical shift as the internal standard ($CDCl_3$: 7.26 ppm ^{1}H, 77.0 ppm ^{13}C) or in D_2O using the solvent as the internal standard in ^{1}H NMR (D_2O: 4.79 ppm ^{1}H). The ^{1}H NMR were acquired without heteroatom decoupling. The ^{13}C and ^{31}P NMR were acquired with hydrogen atom decoupling. ^{1}H NMR integrals of the resonances corresponding to residues at the reducing end are reported as non-integer numbers and the sum of the integrals of α and β anomers is set to 1. High resolution mass spectra were obtained using a 6210 ESI-TOF mass spectrometer (Agilent).

Calf intestine alkaline phosphatase (AP) was purchased from Sigma-Aldrich. α-2,6-Sialyltransferase from *Photobacterium damsela* was purchased from Sigma-Aldrich. CMP-Neu5Ac donor was purchased from Roche.

2 Building blocks for AGA

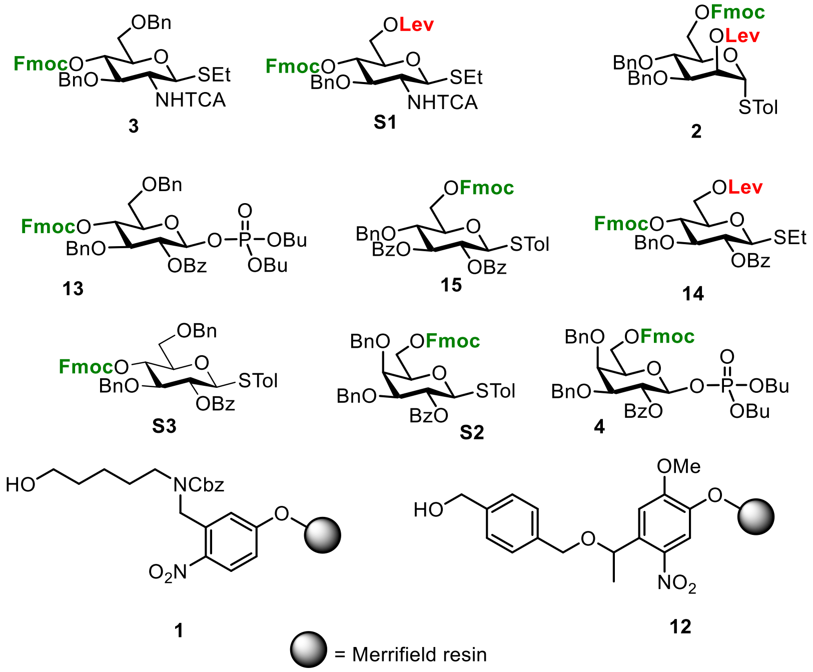

Figure S1. Building blocks and resins utilized for AGA.

All BBs were purchased from GlycoUniverse, except from **4** and **13** that were synthesized according to literature procedures.[1-4] Merrifield resin equipped photocleavable linkers (**1**, loading 0.35 mmol/g and **12** loading 0.34 mmol/g) were prepared according to literature procedures.[5]

3 Automated Glycan Assembly

3.1 General materials and method

The automated syntheses were performed on a home built synthesizer developed at the Max Planck Institute of Colloids and Interfaces. All solvents used were HPLC-grade. The solvents used for the building block, activator, TMSOTf and capping solutions were taken from an anhydrous solvent system (J.C. Meyer) and further dried with molecular sieves (4 Å) for moisture sensitive solutions. The building blocks were co-evaporated three times with toluene and dried for 1 h on high vacuum before use. Oven dried, argon flushed flasks were used to prepare all moisture sensitive solutions. Activator, capping, deprotection, acidic wash, and building block solutions were freshly prepared and kept under argon during the automation run. All yields of products obtained by AGA were calculated on the basis of resin loading. Resin loading was determined following previously established procedures.[6]

3.2 Preparation of stock solutions

- **Building block solution**: Between 5 to 6.5 equiv/glycosylation of building block (depending on the BB, see Module C1 and C2) were dissolved in CH_2Cl_2 (1 mL per glycosylation).
- **NIS/TfOH activator solution**: 1.35 g (6.0 mmol) of recrystallized NIS was dissolved in 40 mL of a 2:1 v/v mixture of anhydrous CH_2Cl_2 and anhydrous dioxane. Then triflic acid (55 µL, 0.6 mmol) was added. The solution was kept at 0 °C for the duration of the automation run.
- **Fmoc deprotection solution A**: A solution of 20% piperidine in DMF (v/v) was prepared.
- **Fmoc deprotection solution B**: A solution of 20% triethylamine in DMF (v/v) was prepared.
- **Lev deprotection solution**: Hydrazine acetate (550 mg, 5.97 mmol) was dissolved in pyridine/AcOH/H_2O (40 mL, v/v, 32:8:2) and sonicated for 10 min.
- **TMSOTf solution**: TMSOTf (0.45 mL, 2.49 mmol) was added to CH_2Cl_2 (40 mL) or for glycosyl phosphate activation; TMSOTf (0.9 mL, 5.0 mmol) was added to CH_2Cl_2 (40 mL).
- **Capping solution**: A solution of 10% acetic anhydride and 2% methanesulfonic acid in CH_2Cl_2 (v/v) was prepared.

3.3 Modules for automated synthesis

Module A: Resin preparation for synthesis (20 min)

All automated syntheses were performed on 0.0135 mmol scale. Resin (**1**, 45 mg or **12**, 45 mg) was placed in the reaction vessel and swollen in CH_2Cl_2 for 20 min at rt prior to synthesis. During this time, all reagent lines needed for the synthesis were washed and primed. After swelling, the resin was washed with DMF, THF, and CH_2Cl_2 (three times each with 2 mL for 25 s).

Module B: Acidic wash with TMSOTf solution (20 min)

The resin was swollen in 2 mL CH_2Cl_2 and the temperature of the reaction vessel was adjusted to -20 °C. Upon reaching the set temperature, TMSOTf solution (1 mL) was added drop wise to the reaction vessel. After bubbling for 3 min, the acidic solution was drained and the resin was washed with 2 mL CH_2Cl_2 for 25 s.

Action	Cycles	Solution	Amount	T (°C)	Incubation time
Cooling	-	-	-	-20	(15 min)*
Deliver	1	CH_2Cl_2	2 mL	-20	-
Deliver	1	TMSOTf solution	1 mL	-20	3 min
Wash	1	CH_2Cl_2	2 mL	-20	25 sec

*Time required to reach the desired temperature.

Module C1: Thioglycoside glycosylation (35 - 55 min)

The building block solution (6.5 equiv. of BB in 1 mL of CH_2Cl_2 per glycosylation) was delivered to the reaction vessel. After the set temperature was reached, the reaction was started by slow addition of the NIS/TfOH activator solution (1.0 mL). After completion of the reaction, the solution was drained and the resin was washed with CH_2Cl_2, CH_2Cl_2:dioxane (1:2, 3 mL for 20 s) and CH_2Cl_2 (two times, each with 2 mL for 25 s). The temperature of the reaction vessel was increased to 25 °C for the next module.

Action	Cycles	Solution	Amount	T (°C)	Incubation time
Cooling	-	-	-	T_1	-
Deliver	1	BB solution	1 mL	T_1	-
Deliver	1	NIS/TfOH activator solution	1 mL	T_1	-
Reaction time	1			T_1<br>to T_2	t_1<br>t_2
Wash	1	CH_2Cl_2	2 mL	T_2	5 sec
Wash	1	CH_2Cl_2: dioxane (1:2)	2 mL	T_2	20 sec
Heating	-	-	-	25	-
Wash	2	CH_2Cl_2	2 mL	> 0	25 sec

Module C2: Glycosyl phosphate glycosylation (45 min)

The building block solution (5 equiv. of BB in 1 mL of CH_2Cl_2 per glycosylation) was delivered to the reaction vessel. After the set temperature was reached, the reaction was started by drop wise addition of the TMSOTf solution (1.0 mL, same equiv). After completion of the reaction, the solution was drained and the resin washed with CH_2Cl_2 (six times, each with 2 mL for 25 s). The temperature of the reaction vessel was increased to 25 °C for the next module.

Action	Cycles	Solution	Amount	T (°C)	Incubation time
Cooling	-	-	-	T_1	-
Deliver	1	BB solution	1 mL	T_1	-
Deliver	1	TMSOTf solution	1 mL	T_1	-
Reaction time	1			T_1<br>to T_2	t_1<br>t_2
Wash	1	CH_2Cl_2	2 mL	T_2	5 sec
Heating	-	-	-	25	-
Wash	6	CH_2Cl_2	2 mL	> 0	25 sec

Module D: Capping (30 min)

The resin was washed with DMF (two times with 2 mL for 25 s) and the temperature of the reaction vessel was adjusted to 25 °C. Pyridine solution (10% in DMF) was delivered (2 mL) into the reaction vessel. After 1 min, the reaction solution was drained and the resin washed with CH_2Cl_2 (three times with 3 mL for 25 s). Capping solution (4 mL) was delivered into the reaction vessel. After 20 min, the reaction solution was drained and the resin washed with CH_2Cl_2 (three times with 3 mL for 25 s).

Action	Cycles	Solution	Amount	T (°C)	Incubation time
Heating	-	-	-	25	(5 min)*
Wash	2	DMF	2 mL	25	25 sec
Deliver	1	10% pyridine in DMF	2 mL	25	1 min
Wash	3	CH_2Cl_2	2 mL	25	25 sec
Deliver	1	capping solution	4 mL	25	20 min
Wash	3	CH_2Cl_2	2 mL	25	25 sec

*Time required to reach the desired temperature.

Module E1: Fmoc deprotection - piperidine (9 min)

The resin was washed with DMF (three times with 2 mL for 25 s) and the temperature of the reaction vessel was adjusted to 25 °C. Fmoc deprotection solution A (2 mL) was delivered to the reaction vessel. After 5 min, the reaction solution was drained and the resin washed with DMF (three times with 3 mL for 25 s) and CH_2Cl_2 (five times each with 2 mL for 25 s). The temperature of the reaction vessel was decreased to -20 °C for the next module.

Action	Cycles	Solution	Amount	T (°C)	Incubation time
Wash	3	DMF	2 mL	25	25 sec
Deliver	1	Fmoc depr. solution	2 mL	25	5 min
Wash	1	DMF	2 mL		
Cooling	-	-	-	-20	-
Wash	3	DMF	2 mL	< 25	25 sec
Wash	5	CH_2Cl_2	2 mL	< 25	25 sec

Module E2: Fmoc deprotection - triethylamine (27 min)

The resin was washed with DMF (three times with 2 mL for 25 s) and the temperature of the reaction vessel was adjusted to 25 °C. Fmoc deprotection solution B (2 mL) was delivered to the reaction vessel. After 5 min, the reaction solution was drained and the resin washed with DMF (three times with 3 mL for 25 s) and CH_2Cl_2 (five times each with 2 mL for 25 s). The entire cycle is repeated three times. The temperature of the reaction vessel was decreased to -20 °C for the next module.

Action	Cycles	Solution	Amount	T (°C)	Incubation time
Wash	3	DMF	2 mL	25	25 sec
Deliver	1	Fmoc depr. solution	2 mL	25	5 min
Wash	1	DMF	2 mL		
Cooling	-	-	-	-20	-
Wash	3	DMF	2 mL	< 25	25 sec
Wash	5	CH_2Cl_2	2 mL	< 25	25 sec

Module E3: Lev deprotection (65 min)

The resin was washed with CH_2Cl_2 (three times with 2 mL for 25 s). CH_2Cl_2 (1.3 mL) was delivered to the reaction vessel and the temperature of the reaction vessel was adjusted to 25 °C. Lev deprotection solution (2 mL) was delivered to the reaction vessel that was kept under pulsed Ar bubbling for 30 min. This procedure was repeated twice. The reaction solution was drained and the resin washed with DMF (three times with 3 mL for 25 s) and CH_2Cl_2 (five times each with 2 mL for 25 s).

Action	Cycles	Solution	Amount	T (°C)	Incubation time
Wash	3	DMF	2 mL	25	25 sec
Deliver	2	Lev depr. solution	2 mL	25	30 min
Wash	1	DMF	2 mL		
Cooling	-	-	-	-20	-
Wash	3	DMF	2 mL	< 25	25 sec
Wash	5	CH_2Cl_2	2 mL	< 25	25 sec

3.4 Post-synthesizer manipulations (Post-AGA)

Module F: On-resin phosphorylation - diphenyl phosphoryl chloride

The resin was suspended in 3 mL of a 5% v/v diphenyl phosphoryl chloride solution (anhydrous CH_2Cl_2/pyridine, 9:1) inside of a fritted syringe. The reaction was shaken for 16 h at rt, after which time the resin was repeatedly washed with DMF (2 x 4 mL), MeOH (2 x 4 mL) and CH_2Cl_2 (2 x 4 mL).

Module G: On-resin phosphorylation - dibenzyl *N,N*-diisopropylphosphoramidite

Two sealed vials were prepared with an argon atmosphere. Vial 1: Dibenzyl N,N-diisopropylphosphoramidite (20 equiv., 0.32 mmol, 110 microliter) was added followed by 2 mL anhydrous CH_2Cl_2. Vial 2: 5-Benzylthio-1H-tetrazole (BTT, 30 equiv., 0.48 mmol, 92 mg) was added and dissolved in 2 mL anhydrous CH_2Cl_2 and 0.5 mL anhydrous ACN. The resin was then first suspended in the contents of Vial 1, by withdrawing the solution into the fritted syringe which contains the resin. Following, the contents of Vial 2 were then also immediately withdrawn into the syringe. The syringe was capped and the reaction was shaken for 1.5 h at room temperature. Upon completion, the reaction solution was removed and the resin was washed repeatedly with CH_2Cl_2.

The resin was then subsequently subjected to oxidation by a mixture of pyridine (2.6 mL), I_2 (0.154 mmol, 40 mg, 22 equiv.), and H_2O (1.4 mL). The resin was suspended in the reaction solution mixture inside of a fritted syringe and gently shaken at room temperature for 2 h. Upon completion, the reaction solution was removed and the resin was washed repeatedly with CH_2Cl_2.

Module H: On-resin hydrolysis

The resin was suspended in a solution THF:MeOH (4:1, 5 mL) and 1 M (aq) LiOH (150 µL) was added. The mixture was gently shaken at rt. After microcleavage (see Module I1) indicated the complete hydrolysis of all ester groups, the resin was repeatedly washed with MeOH (5 x 4 mL) and CH_2Cl_2 (5 x 4 mL). The reaction time is variable and it is indicated for each synthesis.

Module I: Cleavage from solid support

The oligosaccharides were cleaved from the solid support using a continuous-flow photoreactor as described previously.[7] A 15% MeOH in CH_2Cl_2 solvent system was used.

Module I1: Micro-cleavage from solid support

Trace amount of resin (around 20 beads) was dispersed in CH_2Cl_2 (0.1 mL) and irradiated with a UV lamp (6 watt, 356 nm) for 20 min. ACN was then added to the resin and the resulting solution analyzed by MS-Q-TOF or MALDI.

Module J: Off-resin hydrolysis

Crude oligosaccharide was dissolved in 6 mL of THF:H_2O (1:5) and 0.25 M KOH (0.25 mL) was added. The vial was sealed with a septum and the reaction stirred overnight. Upon completion, as determined by MALDI, the reaction was directly loaded onto a size exclusion column.

Module K: Hydrogenolysis[a]

The crude compound was dissolved in solvent (see below for specific conditions). Pd/C (three times the weight of the starting material) was added and the reaction was stirred in a vial equipped with a H_2 balloon. The reaction progress was monitored to avoid undesired side products formation. Upon completion, the reaction was filtered by a PTFE syringe filter and washed with *t*-BuOH and H_2O. The filtrate was concentrated *in vacuo.*

[a]Reaction times and solvent are indicated for each synthesis. THF must be inhibitor free if used as solvent.

Module L: Dephenylation

Crude oligosaccharide was dissolved in 2 mL of AcOH:H_2O (1:1) and PtO_2 (twice the weight of the crude product) was added. The vial was sealed with a septum and equipped with a H_2 balloon. The reaction was stirred overnight. Upon completion, as determined by MALDI, the reaction was passed through a PTFE syringe filter and washed with water. The reaction mixture was then dried by rotary evaporation.

Module M: Purification/Analysis

The purification of the crudes was conducted using a C_{18} silica column or reverse phase HPLC (Agilent 1200 Series or Knauer Azura, **M_{6P}**). The pure compounds were analyzed using analytical HPLC (Agilent 1200 Series)

- **Method M_{1P}:** Sephadex® LH-20 column with H_20:MeOH (1:1) as eluent, isocratic.
- **Method M_{2P}:** (Synergi Hydro RP18 column, Phenomenex, 250 x 10 mm) flow rate of 4.0 mL/min with H_2O (0.1% formic acid) and ACN as eluents [isocratic (5 min), linear gradient to 30%

ACN (30 min), linear gradient to 100% ACN (5 min), isocratic 100% ACN (5 min)]. ELSD Detector: 50 °C.

- **Method M_{3P}:** (Hypercarb column, 150 x 10 mm) flow rate of 3.5 mL/min with H_2O (0.1% formic acid) and ACN as eluents [isocratic (5 min), linear gradient to 30% ACN (30 min), linear gradient to 100% ACN (5 min), isocratic 100% ACN (5 min)]. ELSD Detector: 50 °C.

- **Method M_{4P}:** Manual reverse phase C_{18} silica gel column chromatography: H_2O (10 mL), 0.1% MeOH (10 mL), 1% MeOH (10 mL), 3% MeOH (10 mL), 6% MeOH (10 mL). The phosphorylated compound is eluted with just H_2O.

- **Method M_{5P}:** (Synergi Hydro RP18 column, Phenomenex, 250 x 10 mm) flow rate of 4.0 mL/min with H_2O (0.1% formic acid) and ACN as eluents [isocratic (5 min), linear gradient to 15% ACN (30 min), linear gradient to 100% ACN (5 min), isocratic 100% ACN (5 min)]. ELSD Detector: 50 °C.

- **Method M_{6P}:** (YMC Hydrosphere C18 column, S-5µm, 12nm, 150 x 10 mm) flow rate of 2.0 mL/min with H_2O (0.1% formic acid) and ACN as eluents [isocratic (5 min), linear gradient to 30% ACN (30 min), linear gradient to 100% ACN (5 min), isocratic 100% ACN (5 min)]. Monitored at 214 nm.

- **Method M_{1A}:** (Hypercarb column, 150 x 4.60 mm) flow rate of 0.7 mL / min with H_2O (0.1% formic acid) and ACN as eluents [isocratic (5 min), linear gradient to 30% ACN (30 min), linear gradient to 100% ACN (5 min), isocratic 100% ACN (5 min)]. ELSD Detector: 50 °C.

- **Method M_{2A}:** (Synergi Hydro RP18 column, Phenomenex, 250 x 4.6 mm), flow rate of 1.0 mL/min with H_2O (0.1% formic acid) and ACN as eluents [isocratic (5 min), linear gradient to 30% ACN (30 min), linear gradient to 100% ACN (5 min), isocratic 100% ACN (5 min)].

- **Method M_{3A}:** (Synergi Hydro RP18 column, Phenomenex, 250 x 4.6 mm), flow rate of 1.0 mL/min with H_2O (0.1% formic acid) and ACN as eluents [isocratic (5 min), linear gradient to 15% ACN (30 min), linear gradient to 100% ACN (5 min), isocratic 100% ACN (5 min)].

4 Oligosaccharides syntheses

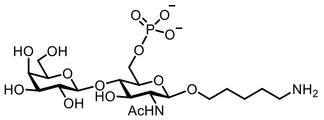

S4

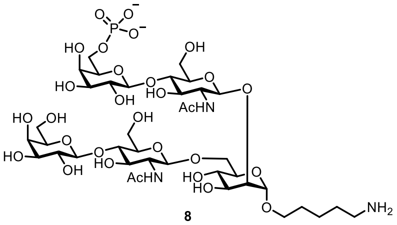

8

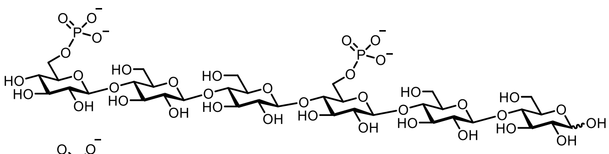

S5

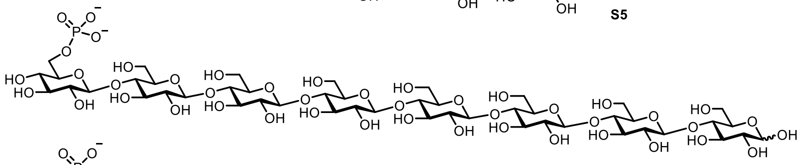

17

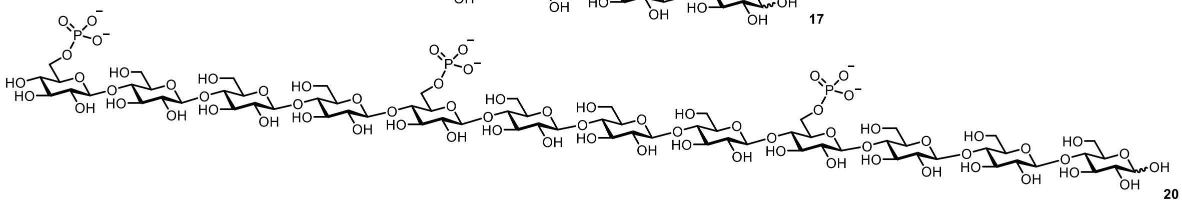

20

Figure S2. Synthesized phosphorylated glycans.

4.1 Synthesis of S4

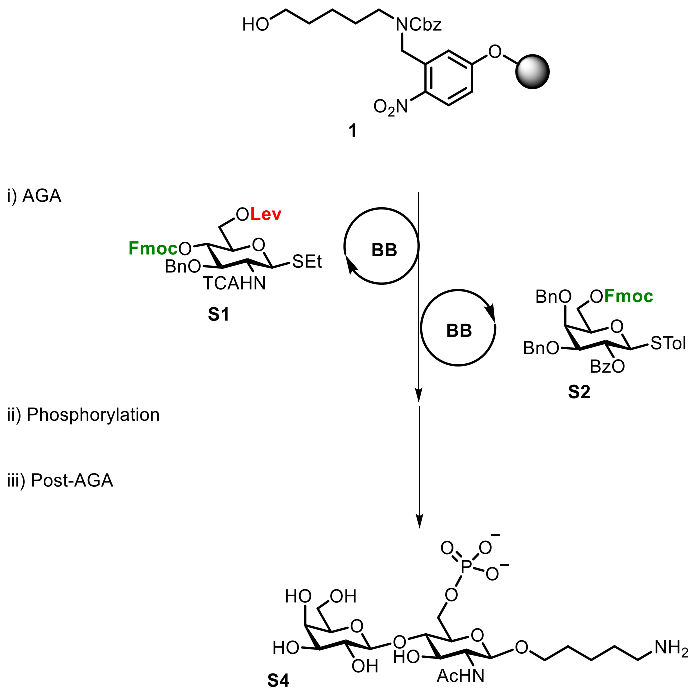

Step		Modules	Notes
AGA		**A**	
	**BB S1**	**B, C1, D, E1**	**C1:** (-25 °C for 15 min, -10 °C for 20 min)
	**BB S2**	**B, 2xC1, E1, D, E3**	**C1:** (-30 °C for 15 min, -30 °C for 20 min)
Phosphorylation		**F**	
Post-AGA	**Photocleavage**	**I**	
	**Hydrogenolysis**	**K**	**K:** 16 h<br>1:1:1:0.25 EtOAc/*t*-BuOH/THF/water (3.25 mL)
	**Dephenylation**	**L**	**L:** 16 h
	**Hydrolysis**	**J**	**J:** 16 h
	**Purification**	**M_{1P}, M_{2P}**	

Compound **S4** was obtained as a white solid (0.4 mg, 5% overall yield).

Analytical data for **S4**:

^{1}H NMR (400 MHz, D_2O) δ 4.54 (d, *J* = 7.9 Hz, 1H), 4.49 (d, *J* = 8.1 Hz, 1H), 4.17 – 3.99 (m, 2H), 3.90 – 3.80 (m, 2H), 3.78 – 3.55 (m, 9H), 3.44 (dd, *J* = 10.1, 7.9 Hz, 1H), 2.93 (t, *J* = 7.6 Hz, 2H), 1.97 (s, 3H), 1.59 (m 4H), 1.36 (m, 2H).

^{13}C NMR (176 MHz, D_2O) δ 174.4, 102.6, 101.1, 77.8, 75.2, 72.5, 72.4, 71.1, 70.1, 68.6, 61.0, 55.1, 39.2, 28.0, 26.2, 22.1, 21.9.

^{31}P NMR (162 MHz, D_2O) δ 3.0.

HRMS (QToF): Calcd for $C_{19}H_{36}N_2O_{14}P$ $[M]^-$ 547.1910; found 574.1896.

^1H NMR of S4 (400 MHz, D_2O)

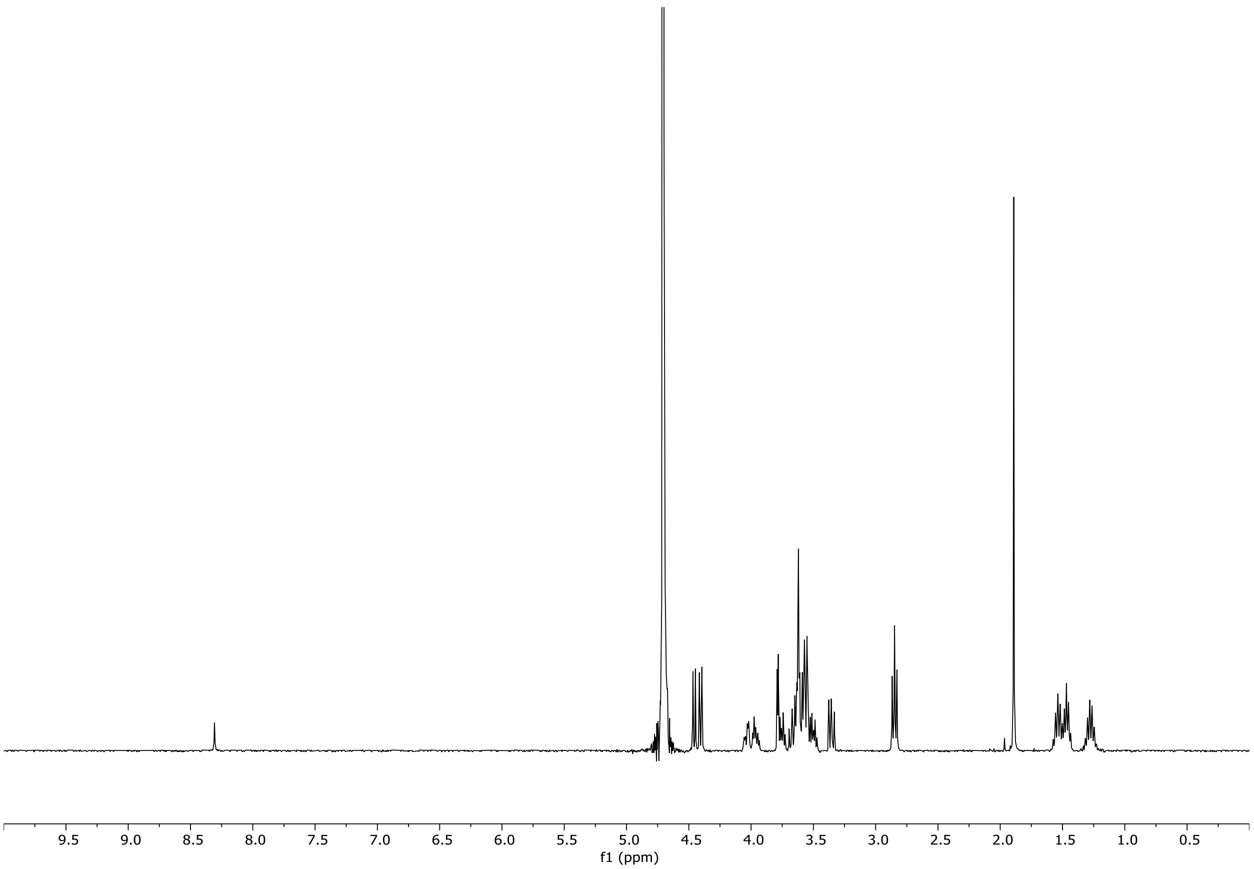

^{31}P NMR of S4 (162 MHz, D_2O)

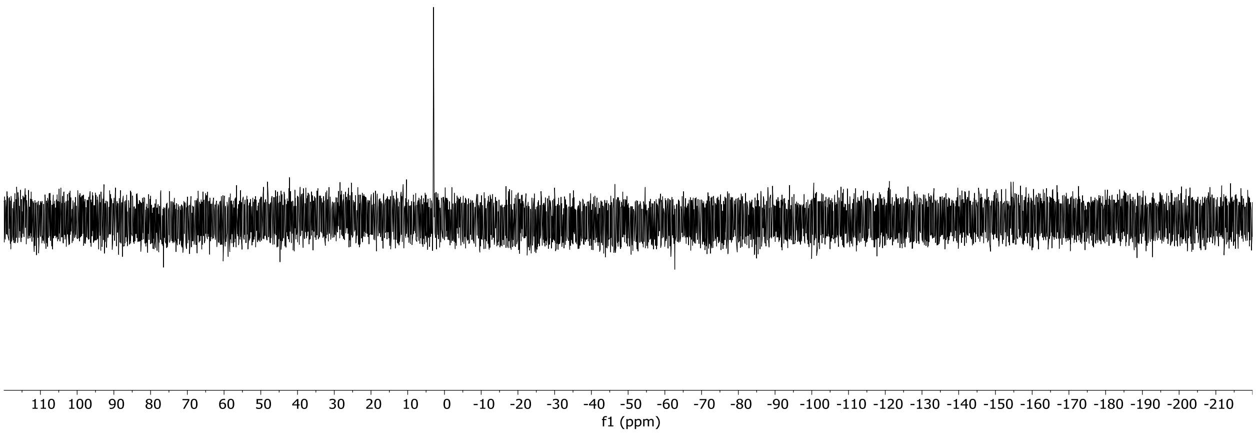

HSQC NMR of S4 (D_2O)

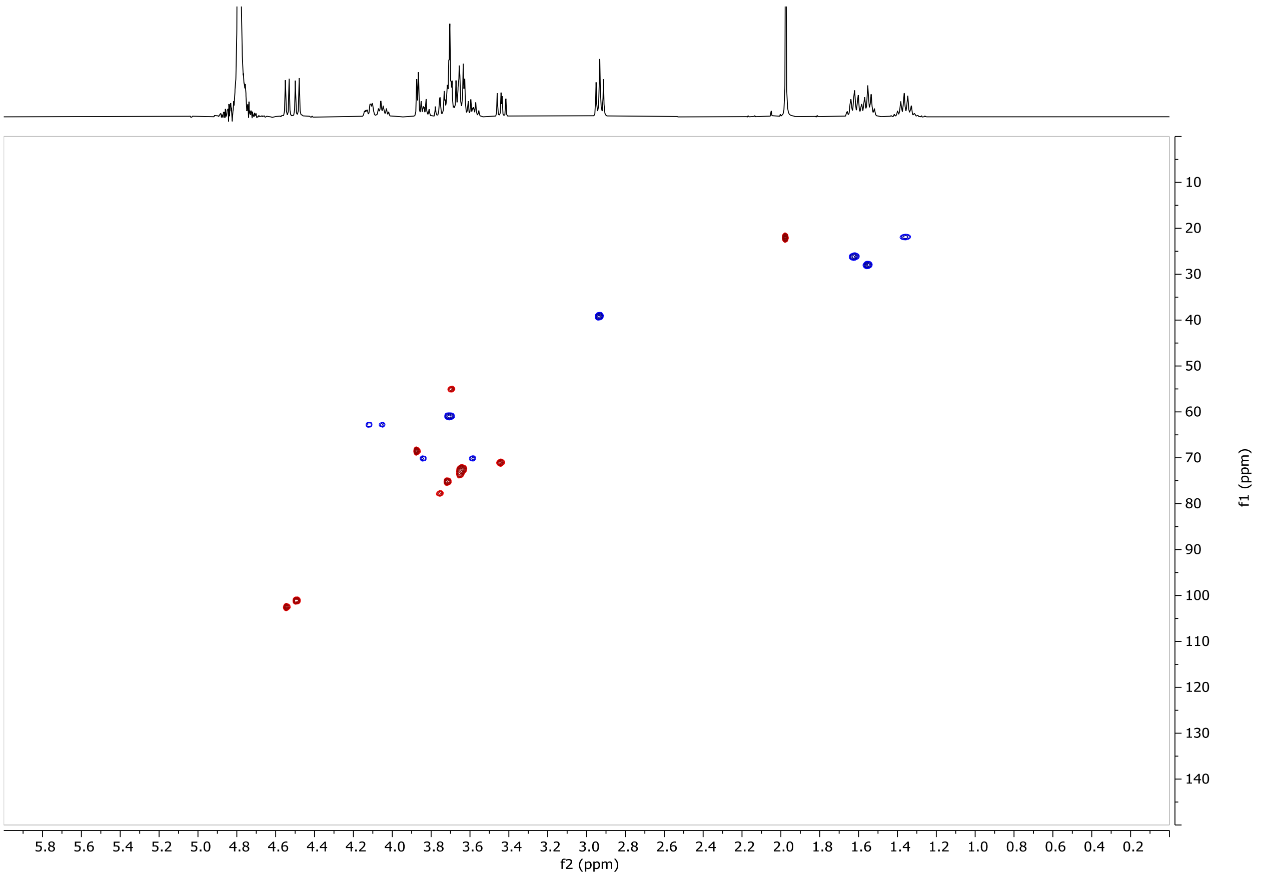

RP-HPLC of S4 (ELSD trace, Method M_{1A}, t_R= 19.21 min)

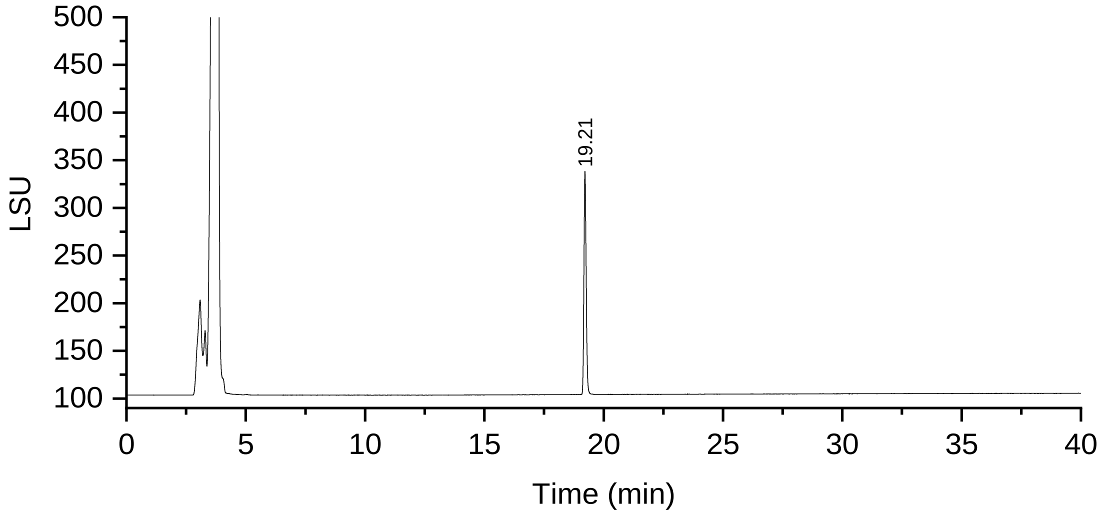

4.2 Synthesis of 8

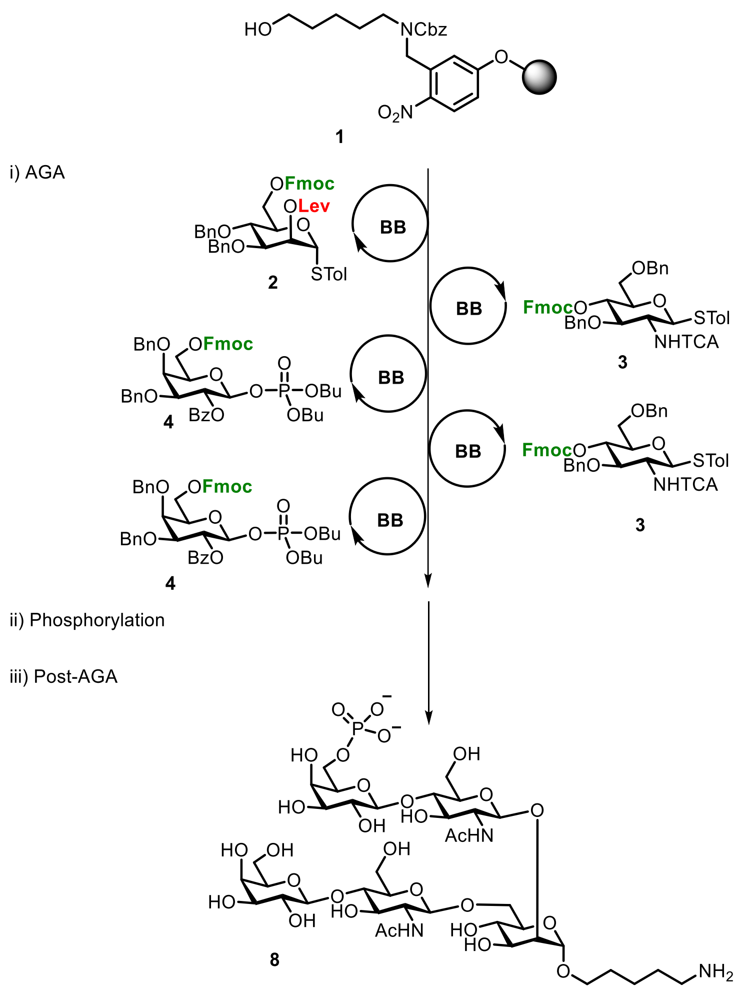

Step		Modules	Notes
AGA		**A**	
	**BB 2**	**B, C1, D, E2**	**C1:** (-20 °C for 5 min, 0 °C for 20 min)
	**BB 3**	**B, C1, D, E2**	**C1:** (-20 °C for 5 min, 0 °C for 20 min)
	**BB 4**	**B, 2xC2, E2, D, E3**	**C2:** (-30 °C for 5 min, -10 °C for 20 min)
	**BB 3**	**B, C1, D, E2**	**C1:** (-20 °C for 5 min, 0 °C for 20 min)
	**BB 4**	**B, 2xC2, D, E2**	**C2:** (-30 °C for 5 min, -10 °C for 20 min)
Phosphorylation		**F**	
Post-AGA	**Photocleavage**	**I**	
	**Hydrogenolysis**	**K**	**K:** 5x w/w Pd/C, 24 h high pressure 1:1:1:.25 EtOAc/*t*-BuOH/THF/water (3.25 mL)
	**Dephenylation**	**L**	**L:** 16 h
	**Hydrolysis**	**J**	**J:** 16 h
	**Purification**	**M_{1P}, M_{3P}**	

Compound **8** was obtained as a white solid (0.75 mg, 4% overall yield).

Analytical data for **8**:

^{1}H NMR (700 MHz, D_2O) 4.52 (d, *J* = 8.3 Hz, 1H), 4.49 (d, *J* = 8.4 Hz, 1H), 4.46 (d, *J* = 7.9 Hz, 1H), 4.43 (d, *J* = 7.8 Hz, 1H), 4.20 (d, *J* = 10.5 Hz, 1H), 3.99 (s, 2H), 3.97 – 3.93 (m, 2H), 3.88 (d, *J* = 3.4 Hz, 1H), 3.85 – 3.76 (m, 5H), 3.75 – 3.70 (m, 5H), 3.69 – 3.60 (m, 9H), 3.58 – 3.44 (m, 6H), 3.38 (t, *J* = 9.8 Hz, 1H), 2.97 (t, *J* = 7.6 Hz, 2H), 2.01 (s, 3H), 1.97 (s, 3H), 1.69 – 1.55 (m, 4H), 1.48 – 1.34 (m, 2H).

^{13}C NMR (176 MHz, D_2O) δ 102.9, 102.7, 101.4, 99.5, 96.4, 79.2, 78.4, 76.5, 75.2, 74.7, 74.2, 74.0, 72.3, 72.1, 71.8, 71.0, 70.2, 69.5, 68.4, 67.9, 67.3, 67.1, 67.1, 62.1, 61.0, 60.8, 60.0, 60.0, 54.8, 54.8, 39.2, 27.9, 26.5, 22.6, 22.6, 22.4, 22.1

^{31}P NMR (162 MHz, D_2O) δ 4.2.

HRMS (QToF): Calcd for $C_{39}H_{69}N_3O_{29}P$ $[M]^-$ 1074.3700; found 1074.3760.

^{1}H NMR of 8 (700 MHz, D_2O)

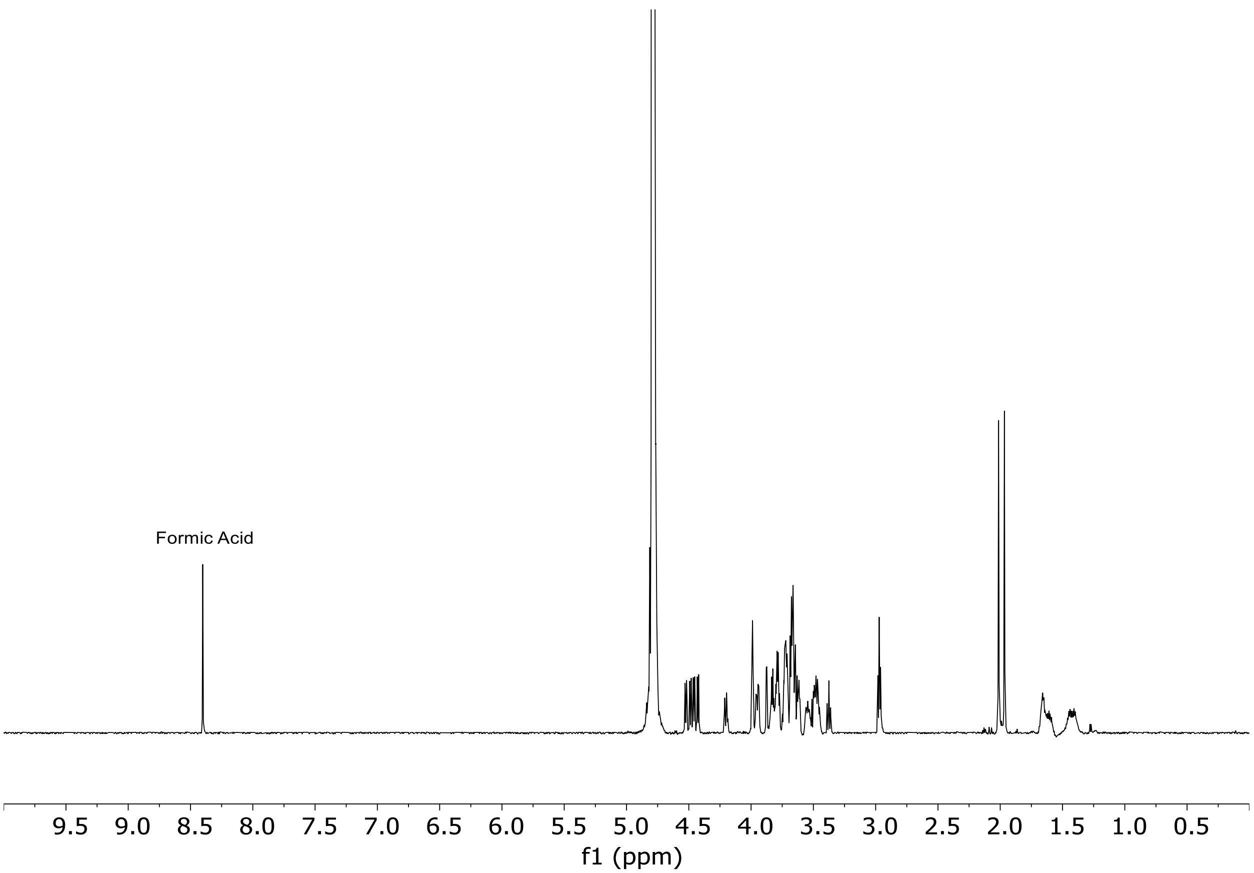

^{31}P NMR 8 (162 MHz, D_2O)

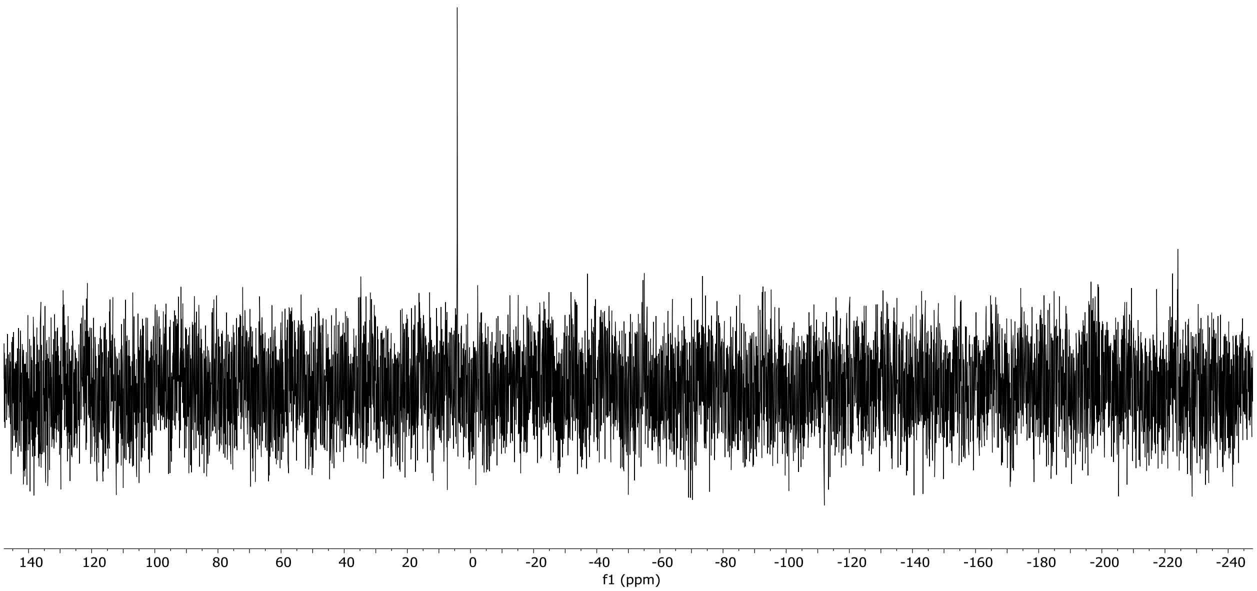

HSQC NMR of 8 (D_2O)

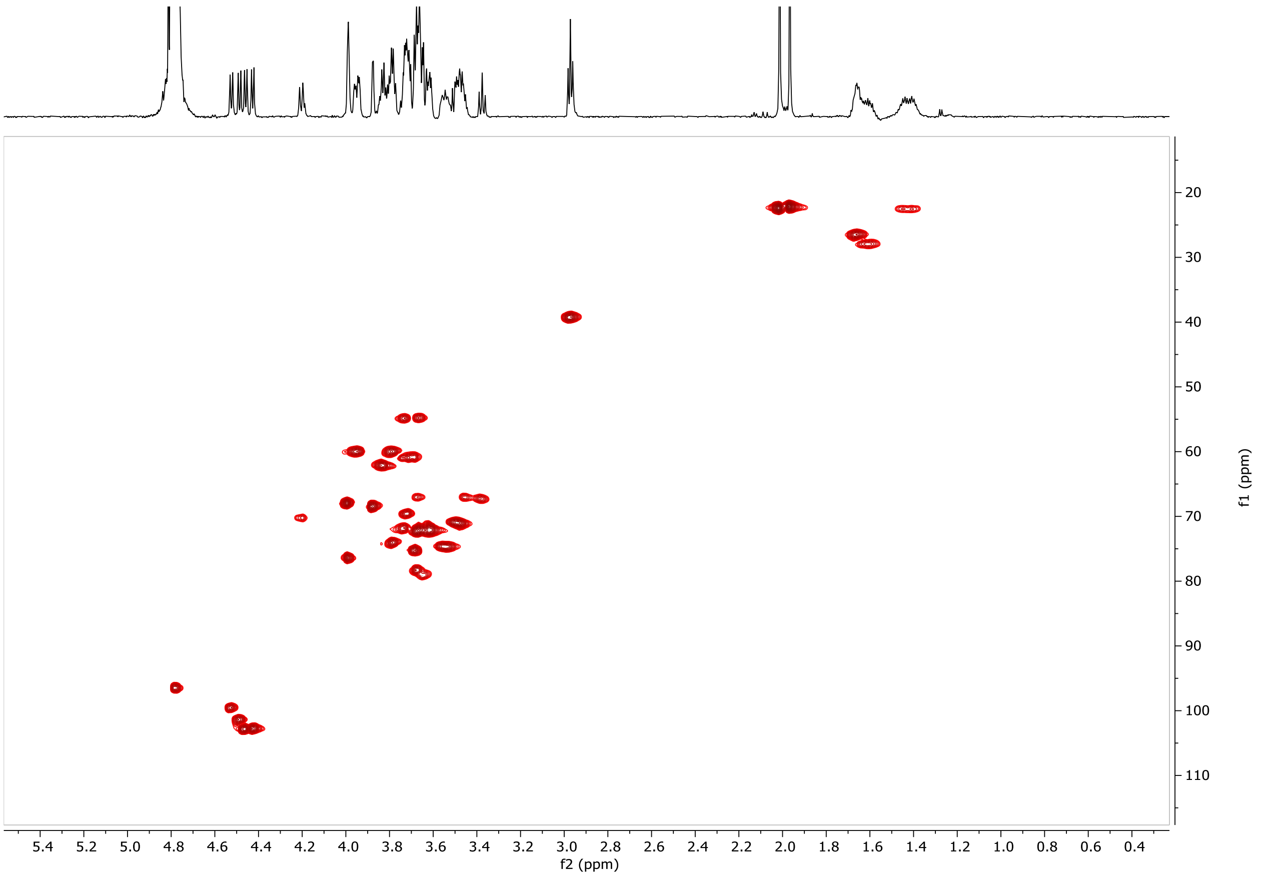

RP-HPLC of 8 (ELSD trace, Method M_{1A}, t_R= 21.78 min)

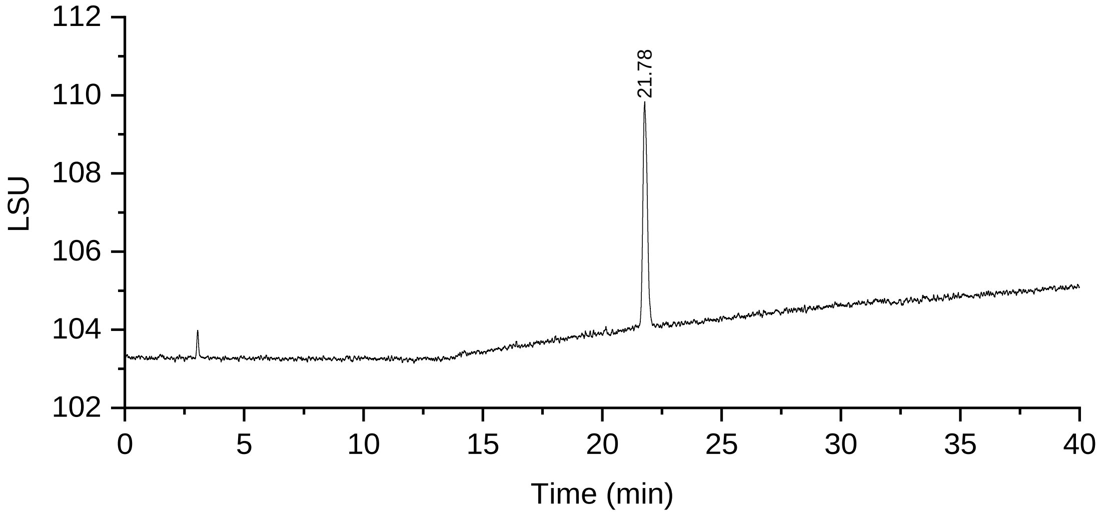

4.3 Synthesis of S5

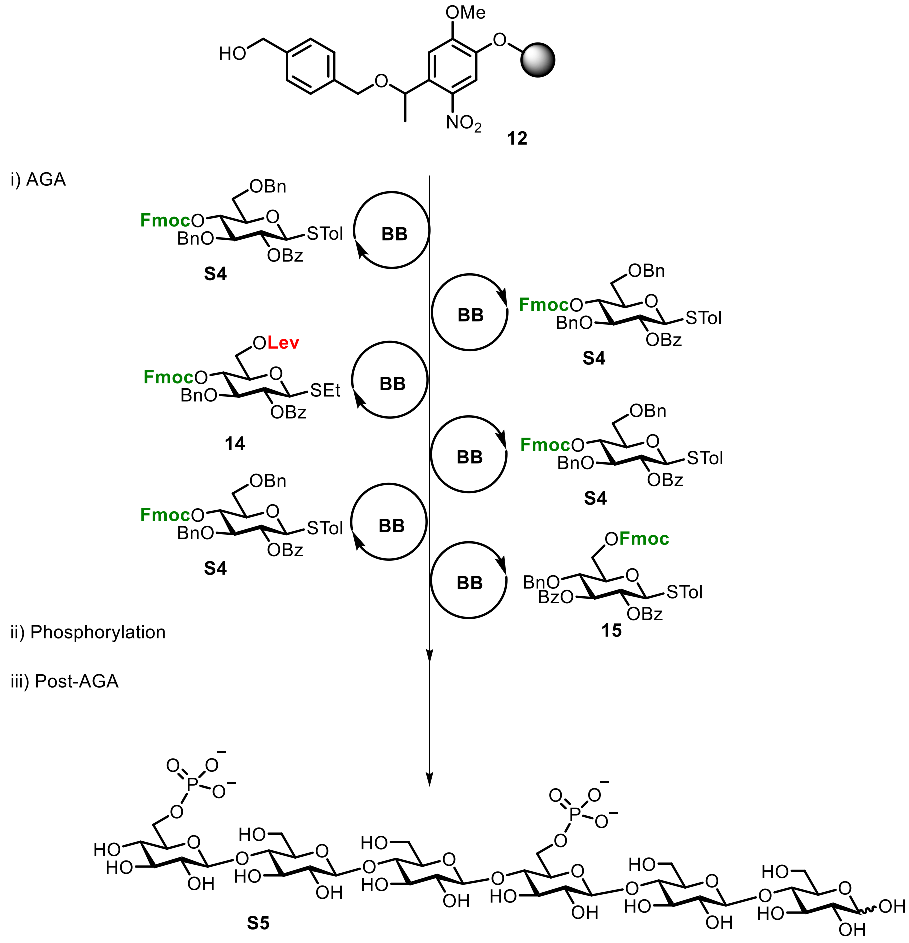

Step		Modules	Notes
AGA		**A**	
	**BB S4**	**B, C1, D, E2**	**C1:** (-20 °C for 5 min, 0 °C for 20 min)
	**BB S4**	**B, C1, D, E2**	**C1:** (-20 °C for 5 min, 0 °C for 20 min)
	**BB 14**	**B, C1, D, E2**	**C1:** (-20 °C for 5 min, 0 °C for 20 min)
	**BB S4**	**B, 2XC1, D, E2**	**C1:** (-20 °C for 5 min, 0 °C for 20 min)
	**BB S4**	**B, C1, D, E2**	**C1:** (-20 °C for 5 min, 0 °C for 20 min)
	**BB 15**	**B, C1, D, E2, E3**	**C1:** (-20 °C for 5 min, 0 °C for 20 min)
Phosphorylation		**G**	
Post-AGA	**Hydrolysis**	**H**	**H:** 4 days
	**Photocleavage**	**I**	
	**Hydrogenolysis**	**K**	**K:** 16 h<br>2:1 *t*-BuOH:water (3 mL)
	**Purification**	**M_{2P}**	

Compound **S5** was obtained as a white solid (1.84 mg, 13% yield).

Analytical data for **S5**:

^{1}H NMR (400 MHz, D_2O) δ 5.16 (d, *J* = 3.8 Hz, 0.4H), 4.60 (d, *J* = 8.0 Hz, 0.6H), 4.56 (d, *J* = 8.0 Hz, 1H), 4.51 – 4.43 (m, 4H), 4.20 – 4.03 (m, 3H), 4.02 – 3.86 (m, 5H), 3.84 – 3.66 (m, 7H), 3.59 (t, *J* = 8.8 Hz, 14H), 3.48 – 3.41 (m, 2H), 3.36 – 3.19 (m, 5H).

^{13}C NMR (101 MHz, D_2O) δ 102.4, 102.2, 95.6, 91.8, 78.5, 77.6, 75.0, 74.0, 73.7, 73.4, 72.7, 71.1, 71.0, 69.8, 68.9, 63.6, 63.4, 63.1, 59.5, 59.5, 59.5, 59.4

^{31}P NMR (162 MHz, D_2O) δ 0.5, 0.2.

HRMS (QToF): Calcd for $C_{36}H_{63}O_{37}P_2$ $[M]^-$ 1149.2529; found 1149.2408.

^{1}H NMR of S5 (400 MHz, D_2O)

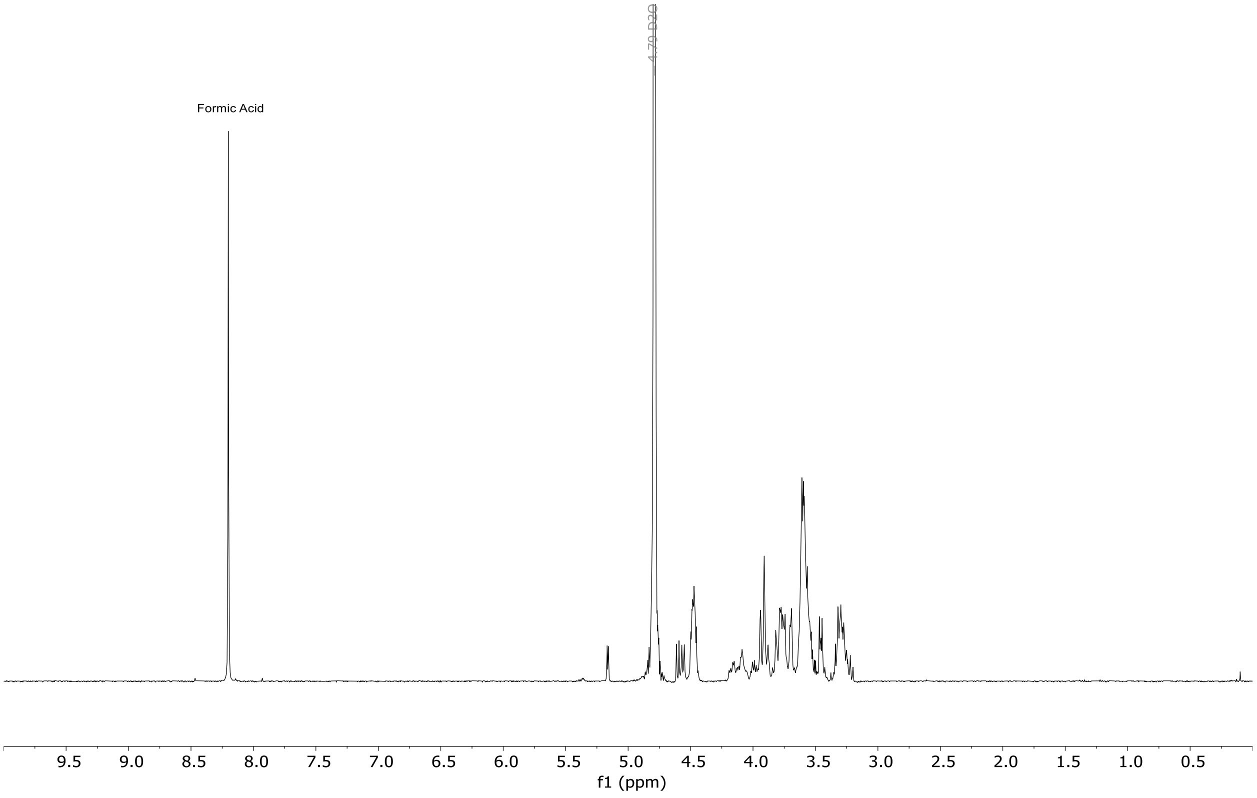

^{31}P NMR of S5 (162 MHz, D_2O)

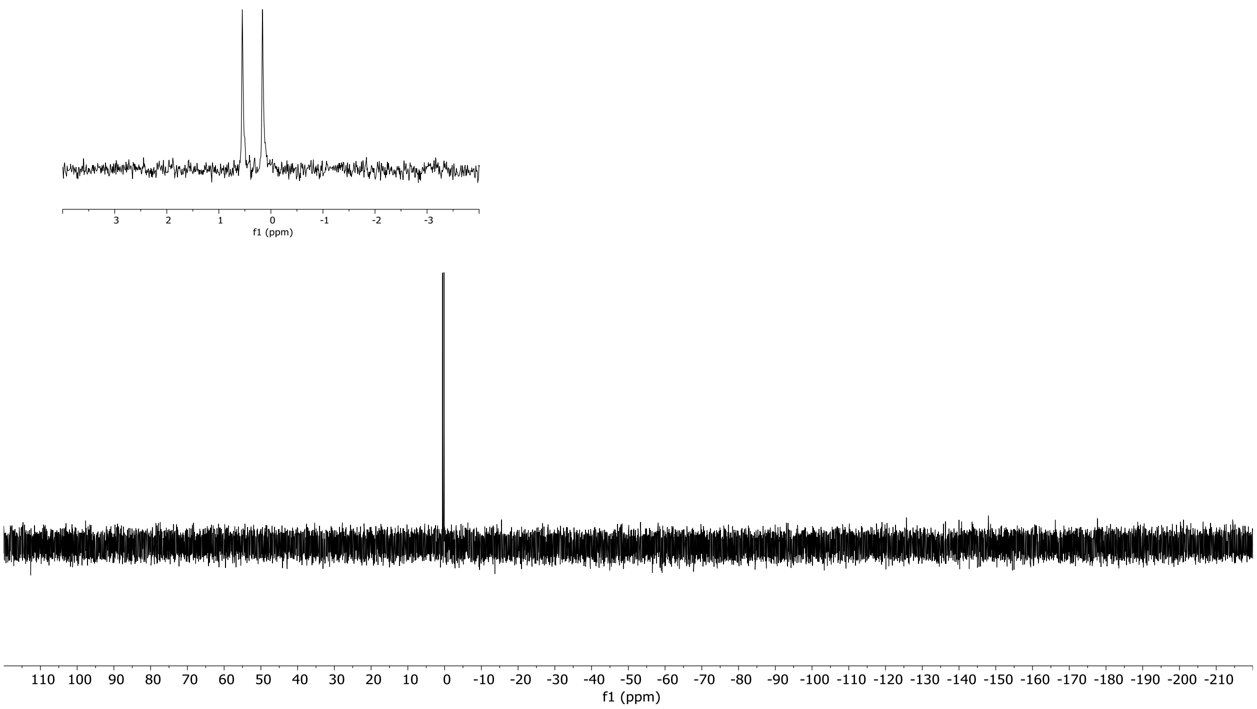

HSQC NMR of S5 (D_2O)

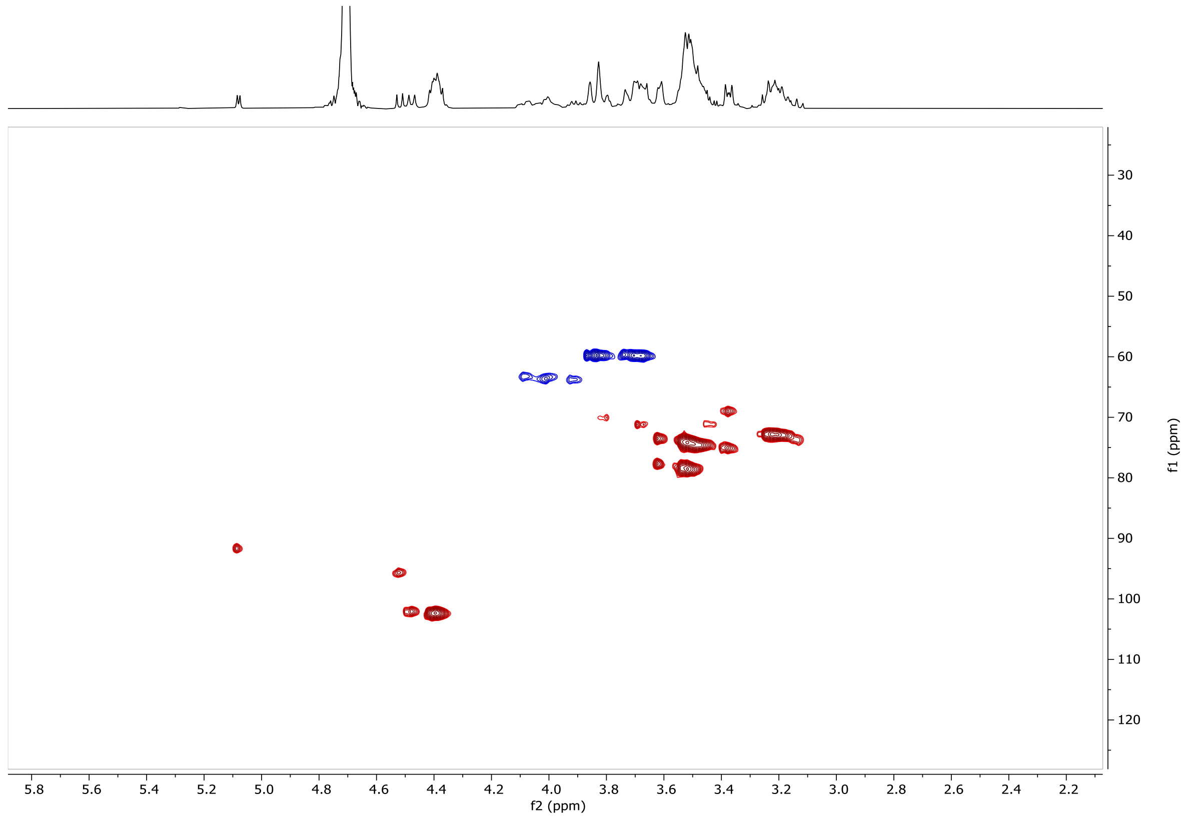

RP-HPLC of S5 (ELSD trace, Method M_{2A}, t_R= 14.46 min)

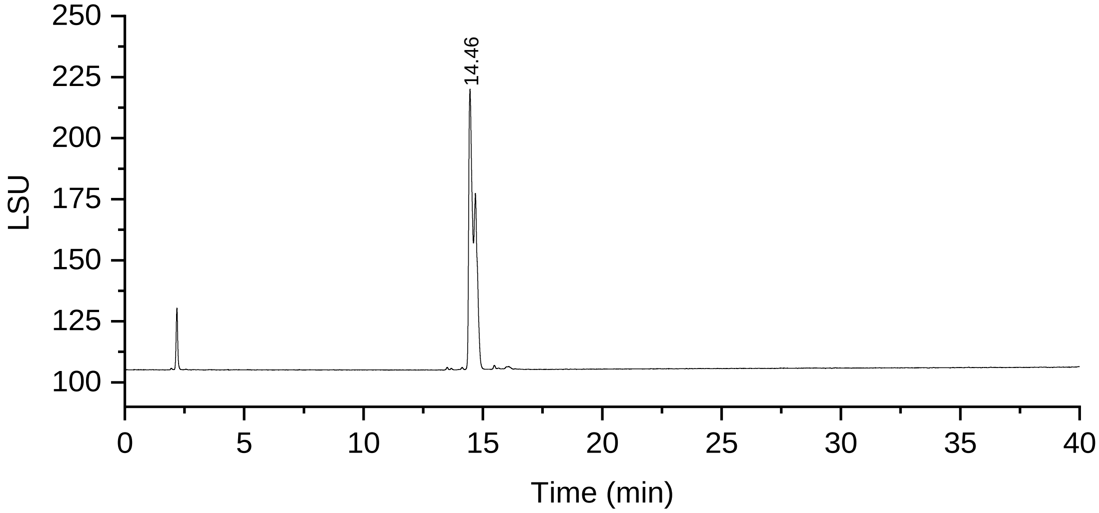

The two peaks correspond to the α and β anomers at the reducing end of the glycan.

4.4 Synthesis of 17

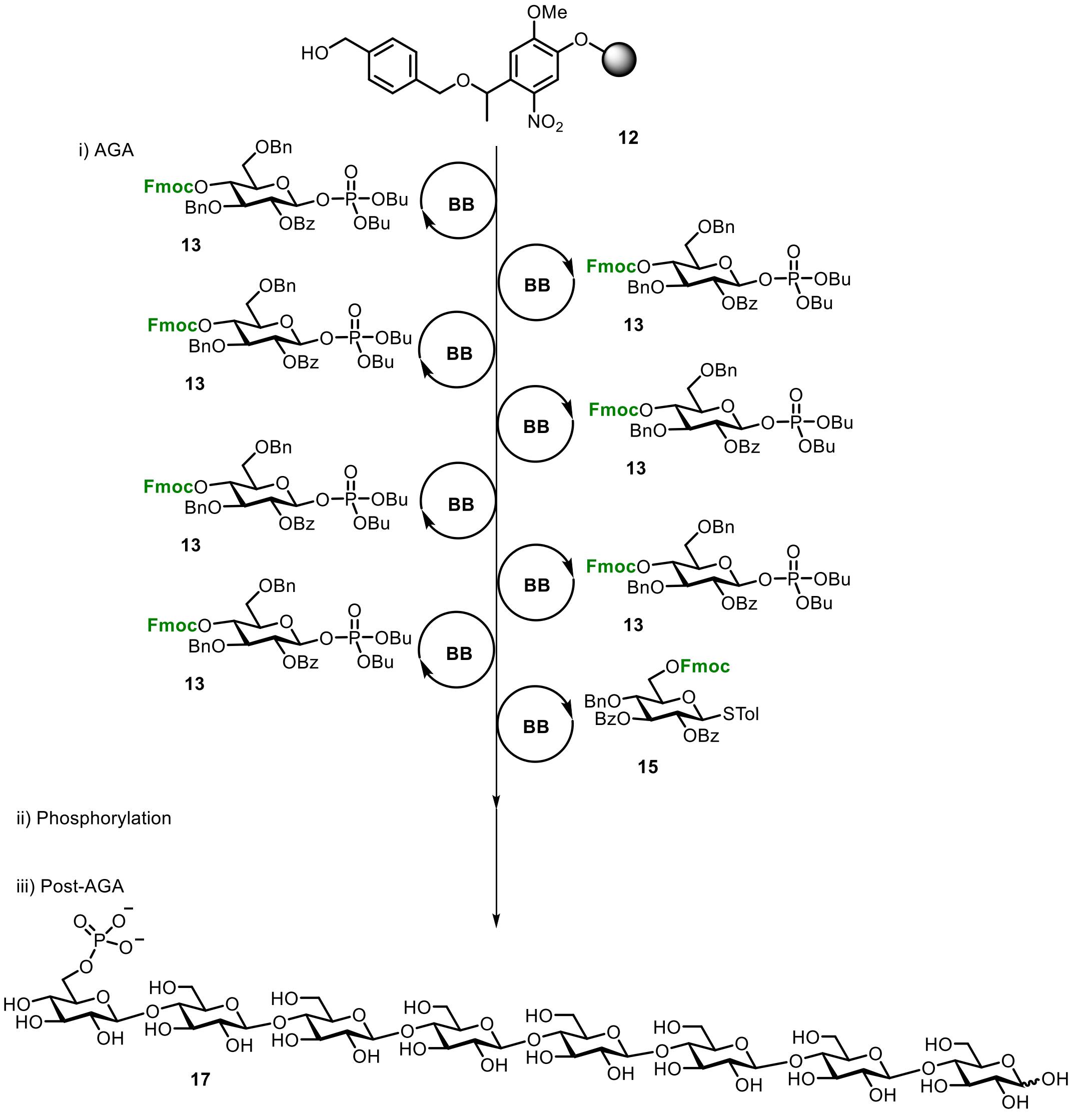

Step		Modules	Notes
AGA		**A**	
	**BB 13**	**B, C2, D, E1**	**C2:** (-30 °C for 5 min, -10 °C for 40 min)
	**BB 13**	**B, C2, D, E1**	**C2:** (-30 °C for 5 min, -10 °C for 40 min)
	**BB 13**	**B, C2, D, E1**	**C2:** (-30 °C for 5 min, -10 °C for 40 min)
	**BB 13**	**B, C2, D, E1**	**C2:** (-30 °C for 5 min, -10 °C for 40 min)
	**BB 13**	**B, C2, D, E1**	**C2:** (-30 °C for 5 min, -10 °C for 40 min)
	**BB 13**	**B, C2, D, E1**	**C2:** (-30 °C for 5 min, -10 °C for 40 min)
	**BB 13**	**B, C2, D, E1**	**C2:** (-30 °C for 5 min, -10 °C for 40 min)
	**BB 15**	**B, 2XC1, D, E1**	**C1:** (-20 °C for 5 min, 0 °C for 20 min)
Phosphorylation		**G**	
Post-AGA	**Hydrolysis**	**H**	**H:** 7 days
	**Photocleavage**	**I**	
	**Hydrogenolysis**	**K**	**K:** 16 h<br>3:1:1 *t*-BuOH:EtOAc:H_2O (5 mL)
	**Purification**	**M_{4P}**	

Compound **17** was obtained as a white solid (3.8 mg, 17% yield).

Analytical data for **17**:

^{1}H NMR (400 MHz, D_2O) δ 5.17 (d, *J* = 3.7 Hz, 0.4H), 4.61 (d, *J* = 8.0 Hz, 0.6H), 4.51 – 4.44 (m, 7H), 3.99 – 3.87 (m, 9H), 3.84 – 3.72 (m, 8H), 3.67 – 3.43 (m, 25H), 3.34 – 3.20 (m, 8H).

^{13}C NMR (101 MHz, D_2O) δ 102.2, 95.6, 91.8, 78.2, 75.0, 74.5, 74.0, 74.0, 73.7, 72.9, 71.3, 68.9, 68.7, 68.7, 62.6, 59.8, 59.7, 59.7

^{31}P NMR (162 MHz, D_2O) δ 4.3.

HRMS (QToF): Calcd for $C_{48}H_{82}O_{44}P$ $[M]^-$ 1393.3922; found 1393.3804.

^{1}H NMR of 17 (400 MHz, D_2O)

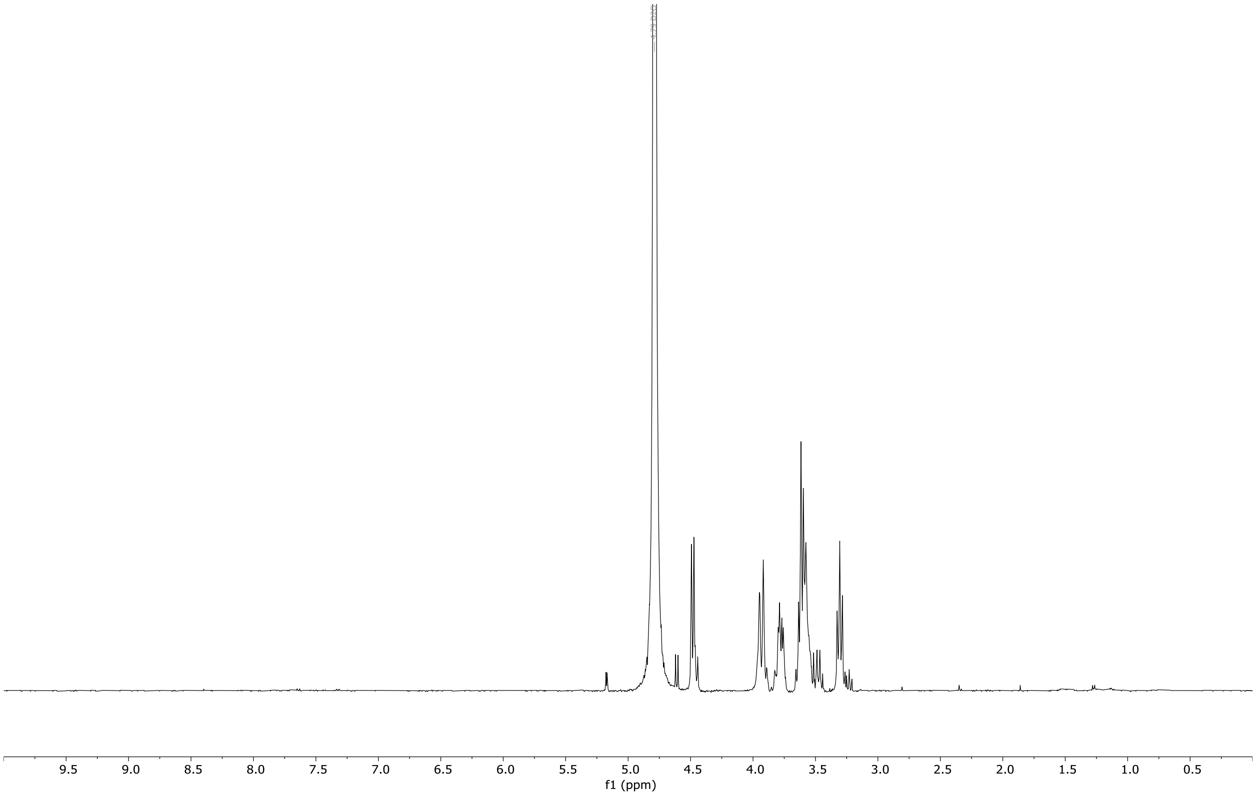

^{31}P NMR of 17 (162 MHz, D_2O)

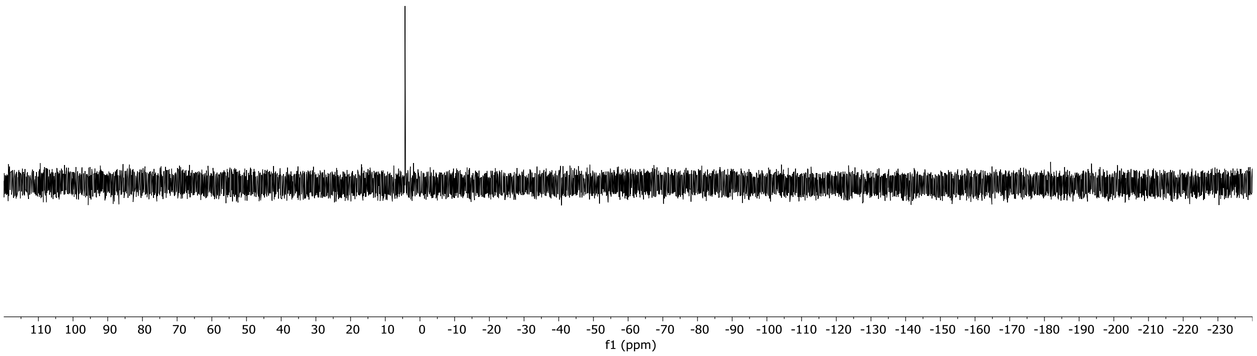

HSQC NMR of 17 (D_2O)

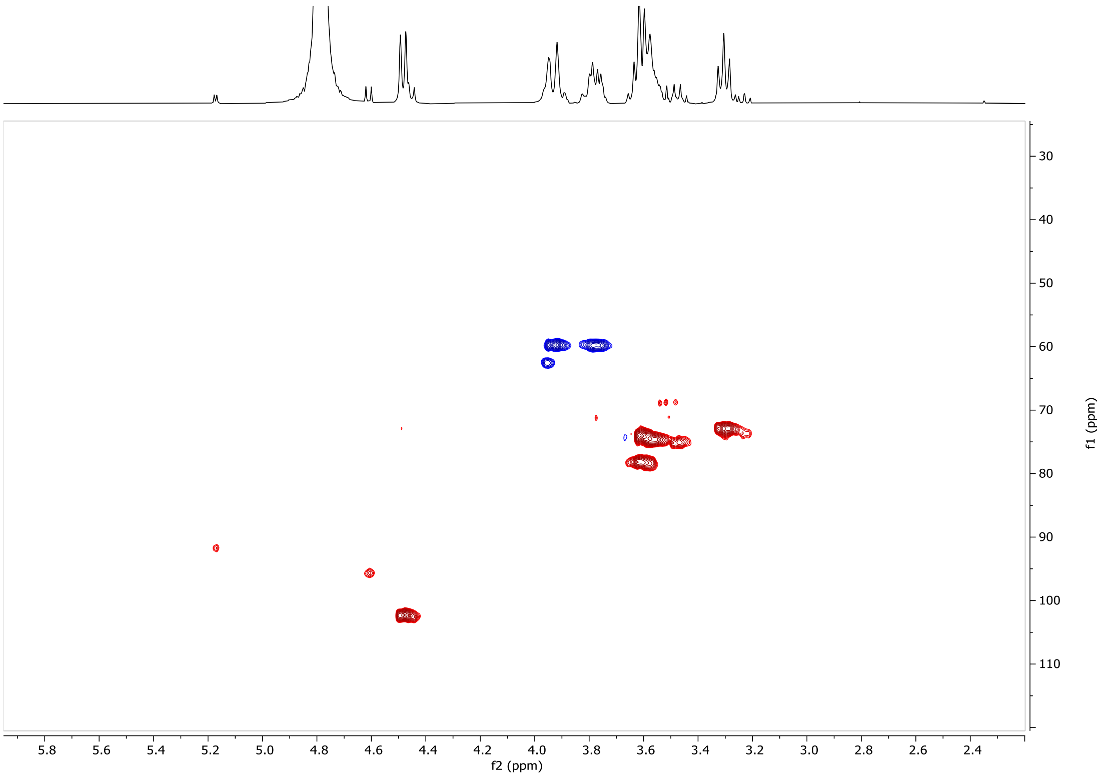

RP-HPLC of 17 (ELSD trace, Method M_{1A}, t_R= 18.58 min)

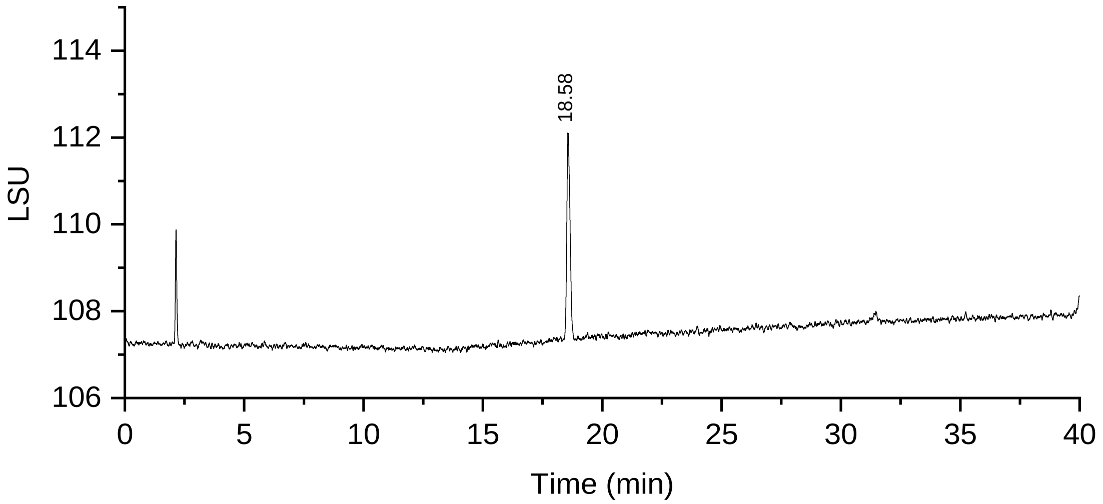

4.5 Synthesis of 20

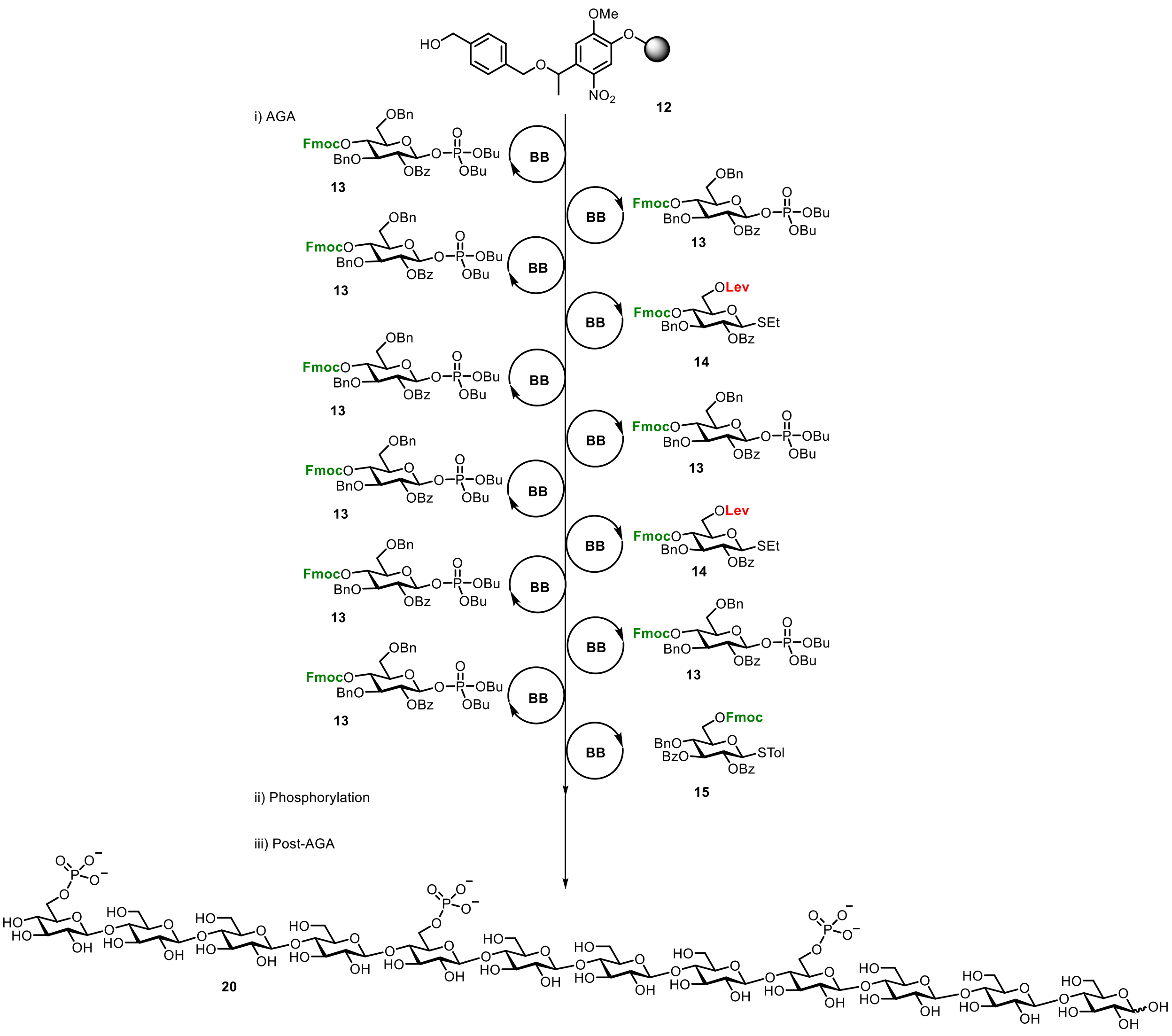

Step		Modules	Notes
AGA		**A**	
	**BB 13**	**B, C2, D, E2**	**C2:** (-30 °C for 5 min, -10 °C for 40 min)
	**BB 13**	**B, C2, D, E2**	**C2:** (-30 °C for 5 min, -10 °C for 40 min)
	**BB 13**	**B, C2, D, E2**	**C2:** (-30 °C for 5 min, -10 °C for 40 min)
	**BB 14**	**B, 2XC1, D, E2**	**C1:** (-20 °C for 5 min, 0 °C for 20 min)
	**BB 13**	**B, C2, D, E2**	**C2:** (-30 °C for 5 min, -10 °C for 40 min)
	**BB 13**	**B, C2, D, E2**	**C2:** (-30 °C for 5 min, -10 °C for 40 min)
	**BB 13**	**B, C2, D, E2**	**C2:** (-30 °C for 5 min, -10 °C for 40 min)
	**BB 14**	**B, 2XC1, D, E2**	**C1:** (-20 °C for 5 min, 0 °C for 20 min)
	**BB 13**	**B, C2, D, E2**	**C2:** (-30 °C for 5 min, -10 °C for 40 min)
	**BB 13**	**B, C2, D, E2**	**C2:** (-30 °C for 5 min, -10 °C for 40 min)
	**BB 13**	**B, C2, D, E2**	**C2:** (-30 °C for 5 min, -10 °C for 40 min)
	**BB 15**	**B, 2XC1, D, E2, E3**	**C1:** (-20 °C for 5 min, 0 °C for 20 min)
Phosphorylation		**G**	2x
Post-AGA	**Hydrolysis**	**H**	**H:** 8 days
	**Photocleavage**	**I**	
	**Hydrogenolysis**	**K**	**K:** 16 h<br>3:1:1 *t*-BuOH:EtOAc:water 1 drop AcOH
	**Purification**	**M_{5P}**	

Compound **20** was obtained as a white solid (0.5 mg, 1% overall yield).

Analytical data for **20**:

^{1}H NMR (400 MHz, D_2O) δ 5.17 (d, *J* = 3.8 Hz, 0.4H), 4.67 (t, *J* = 8.0 Hz, 2H), 4.61 (d, *J* = 8.0 Hz, 0.6H), 4.50 – 4.46 (m, 8H), 4.45 (d, *J* = 8.0 Hz, 1H), 4.08 – 4.01 (m, 2H), 3.98 – 3.90 (m, 12H), 3.78 (d, *J* = 11.3 Hz, 9H), 3.71 (d, *J* = 7.8 Hz, 2H), 3.68 – 3.52 (m, 33H), 3.47 (t, *J* = 9.2 Hz, 2H), 3.34 – 3.27 (m, 10H), 3.24 – 3.20 (m, 2H).

^{13}C NMR (101 MHz, D_2O) 102.2, 102.2, 95.6, 91.8, 78.4, 78.4, 75.2, 74.7, 74.2, 74.0, 73.4, 72.9, 71.0, 70.6, 68.9, 62.4, 62.1, 59.8, 59.8.

^{31}P NMR (162 MHz, D_2O) δ 4.4, 3.6.

HRMS (QToF): Calcd for $C_{72}H_{123}O_{70}P_3$ $[M]^{-2}$ 1100.2644; found 1100.3181.

^{1}H NMR of 20 (400 MHz, D_2O)

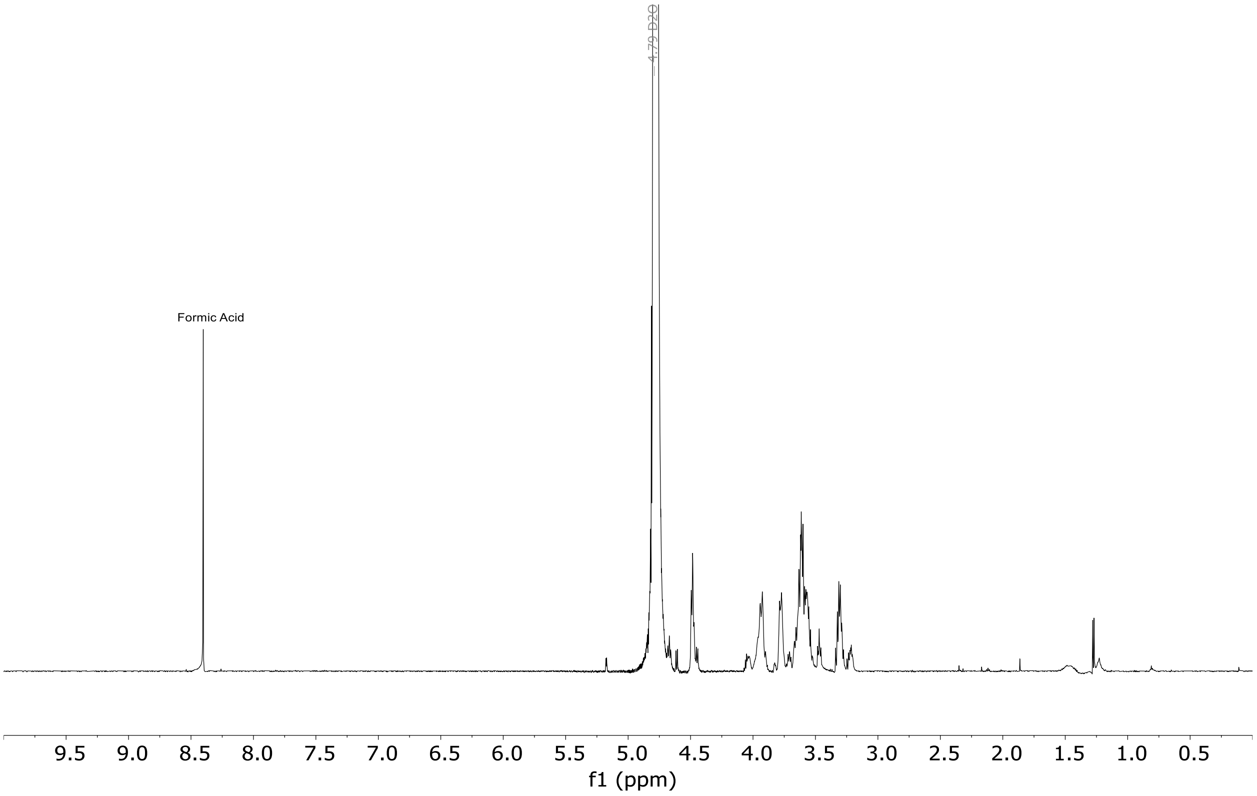

^{31}P NMR of 20 (162 MHz, D_2O)

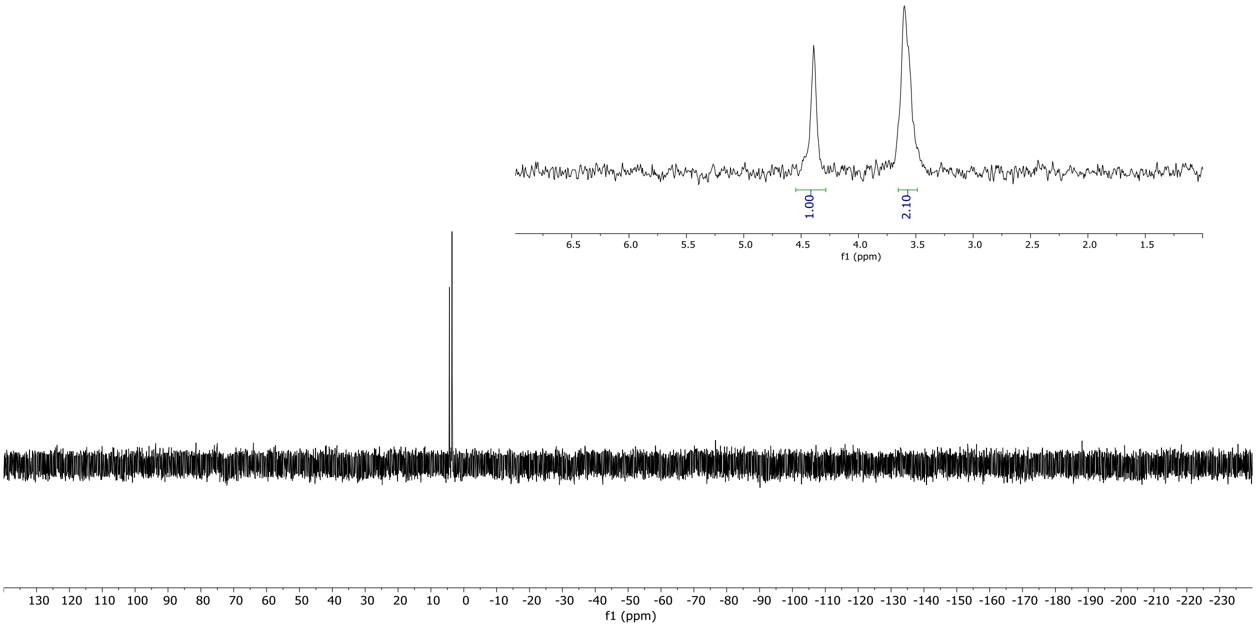

HSQC NMR of 20 (D_2O)

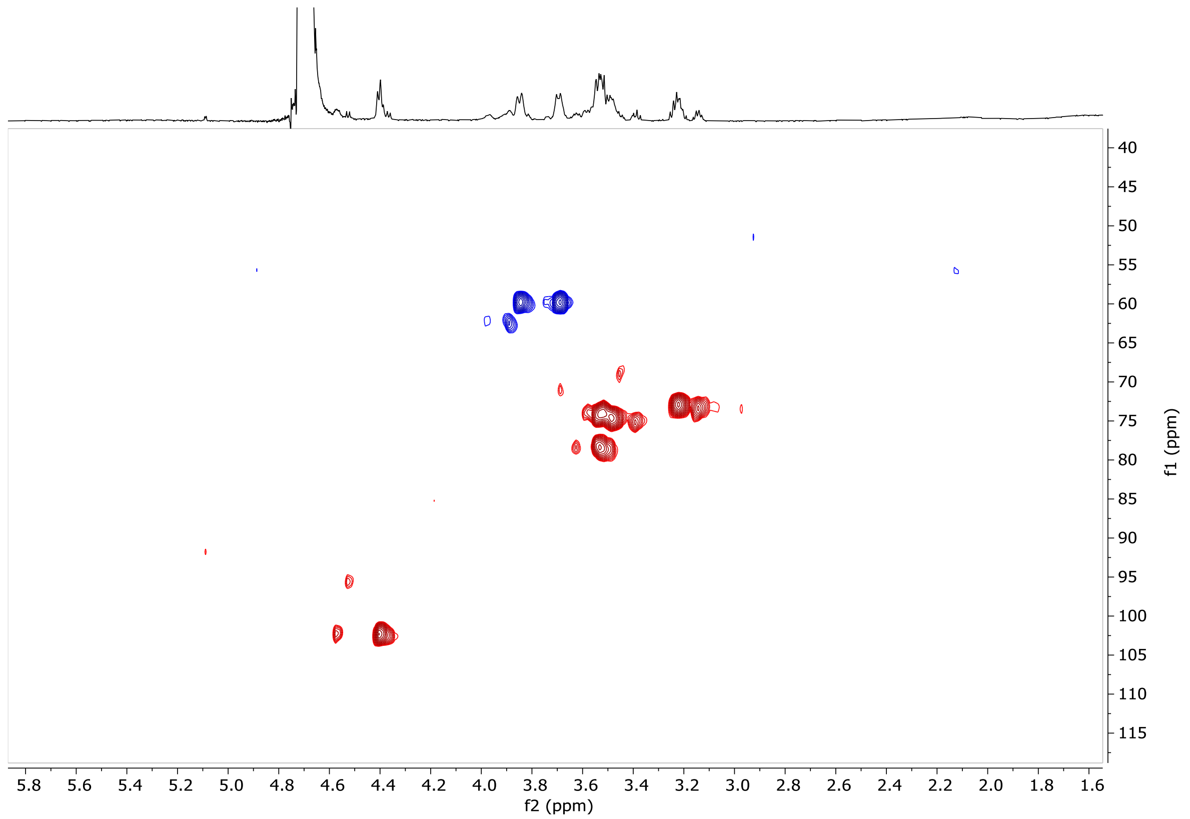

RP-HPLC of 20 (ELSD trace, Method M_{3A}, t_R= 18.01 min)

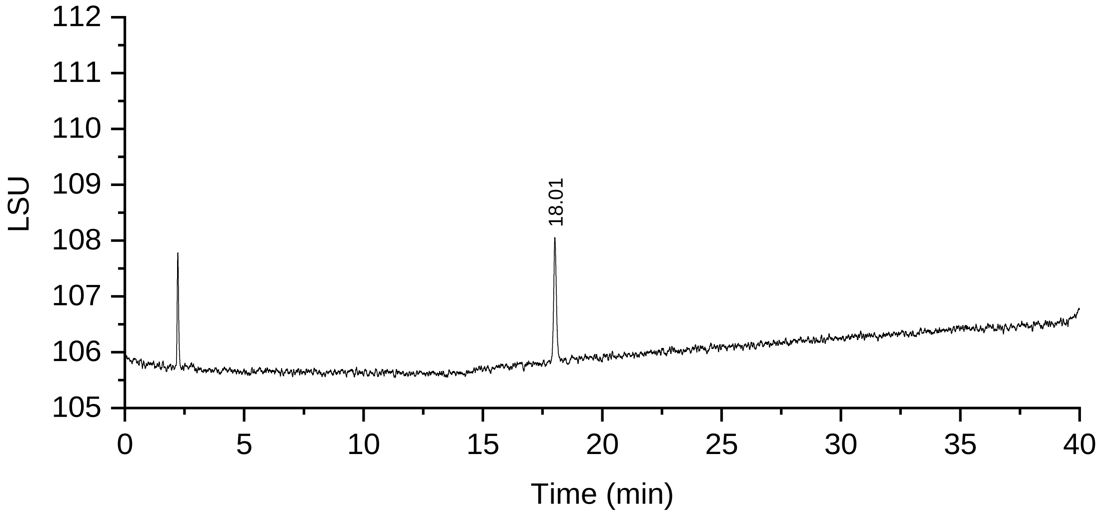

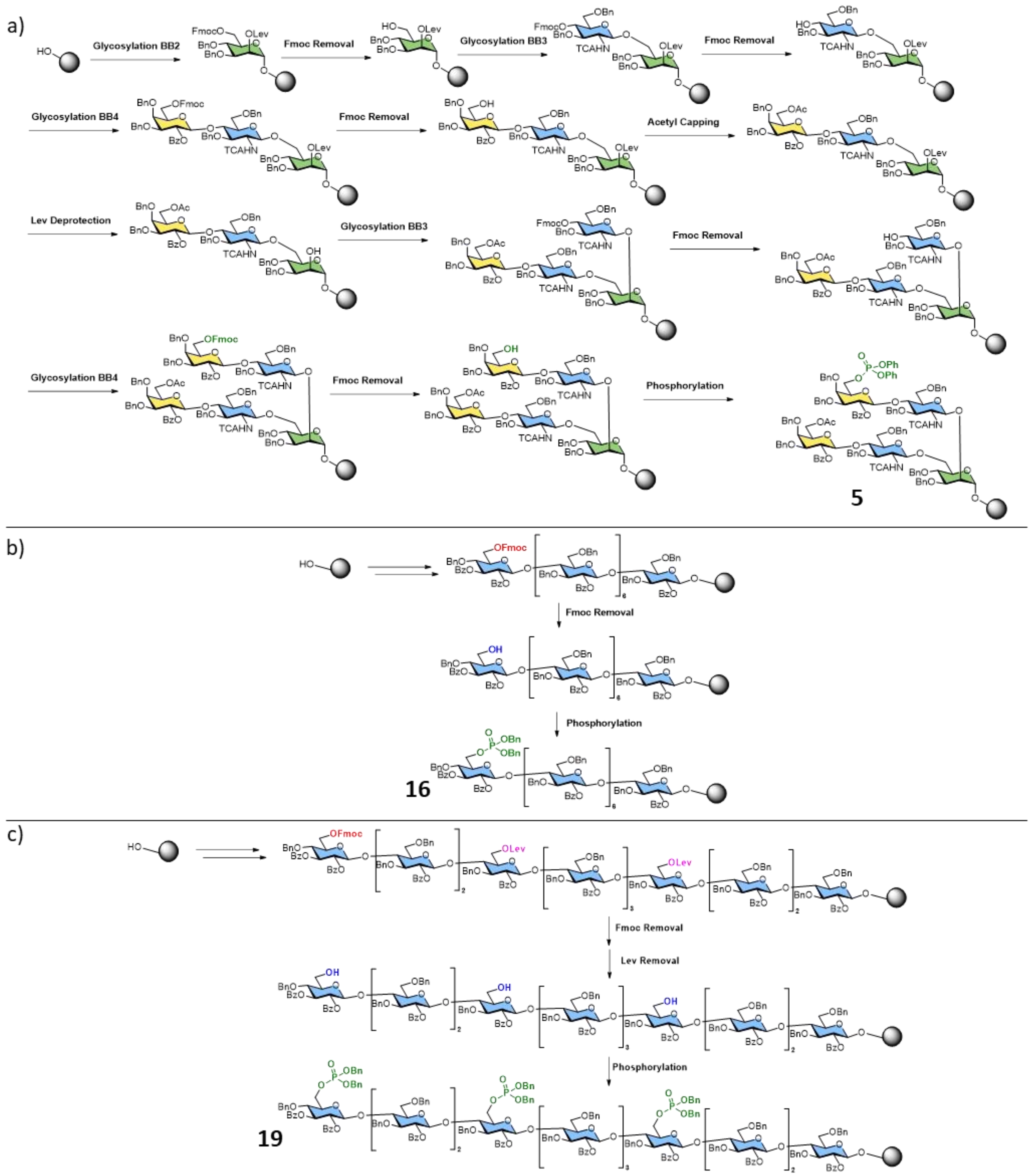

Figure S3. Full synthetic route to on-resin phosphorylated glycans.

5 Regioselective enzymatic sialylation of N-glycan fragment

5.1 Disaccharide phosphate screening

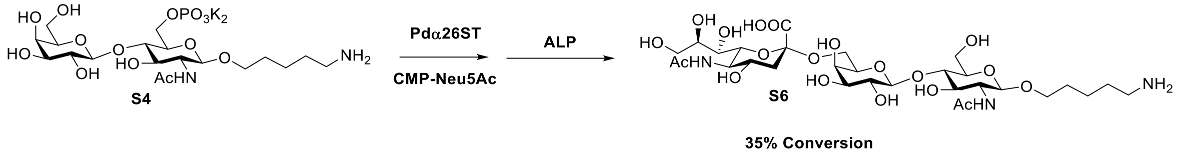

Phosphorylated acceptor **S4** (0.5 µL, 50 mM stock solution), CMP-Neu5Ac donor (1 µL, 50 mM stock solution, 2 equiv), Pdα26ST (2 µL, 10 mU/µL ca.), 5xTRIS buffer (5 µL, 0.5 M, pH 8.0), and H_2O (16.5 µL) were added into a 0.5 mL Eppendorf tube and placed in a 37 °C incubator.

Final conditions: 1 mM of acceptor, 2 mM donor, ca. 800 mU/µmol Pdα26ST, TRIS 100 mM, 25 µL total volume, 0.025 µmol scale.

After 24 h, the reaction was terminated by thermal denaturation at 90 °C for 5 min. ALP (1 µL, 20 U/µL ca.) was then added and the tube placed in an incubator at 37 °C for 24 h. The reaction progress was monitored by reverse phase HPLC (Method $\mathbf{M_{2A}}$).

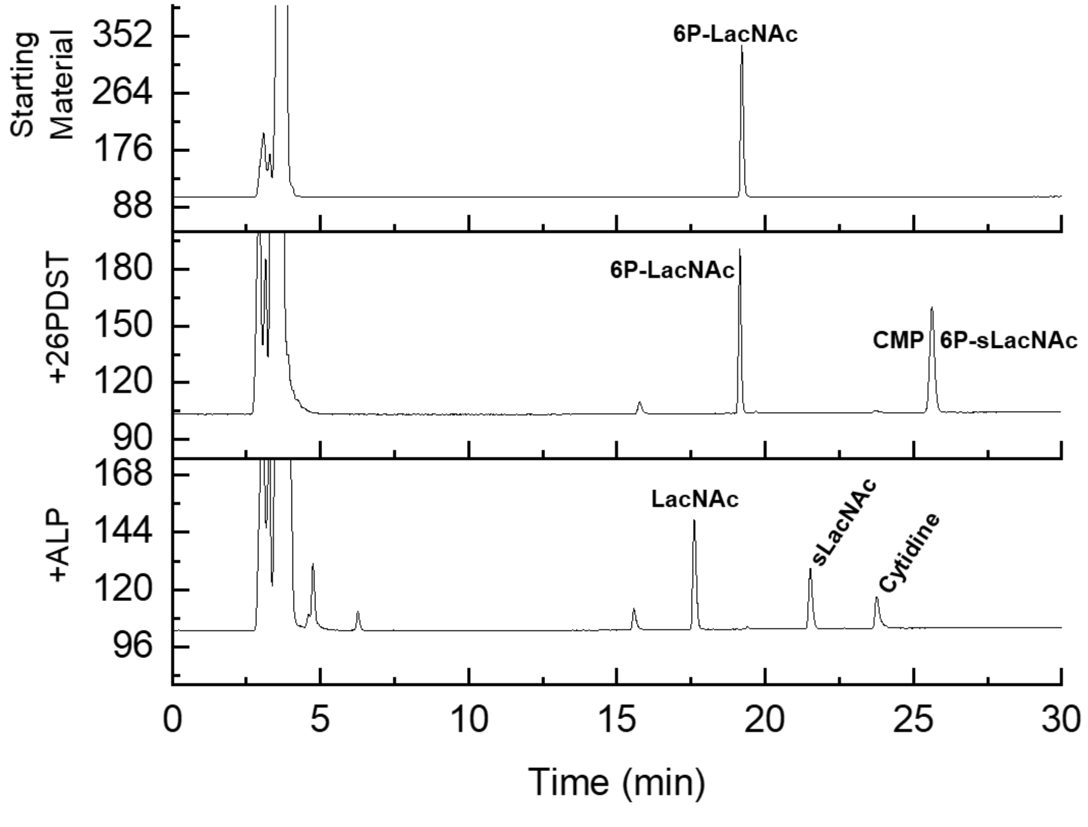

Figure S4. ELSD reverse-phase HPLC traces monitoring the steps of the one-pot sialylation/dephosphorylation of acceptor **S4**. Peaks were determined by inline mass spectrometer.

5.2 Selective sialylation

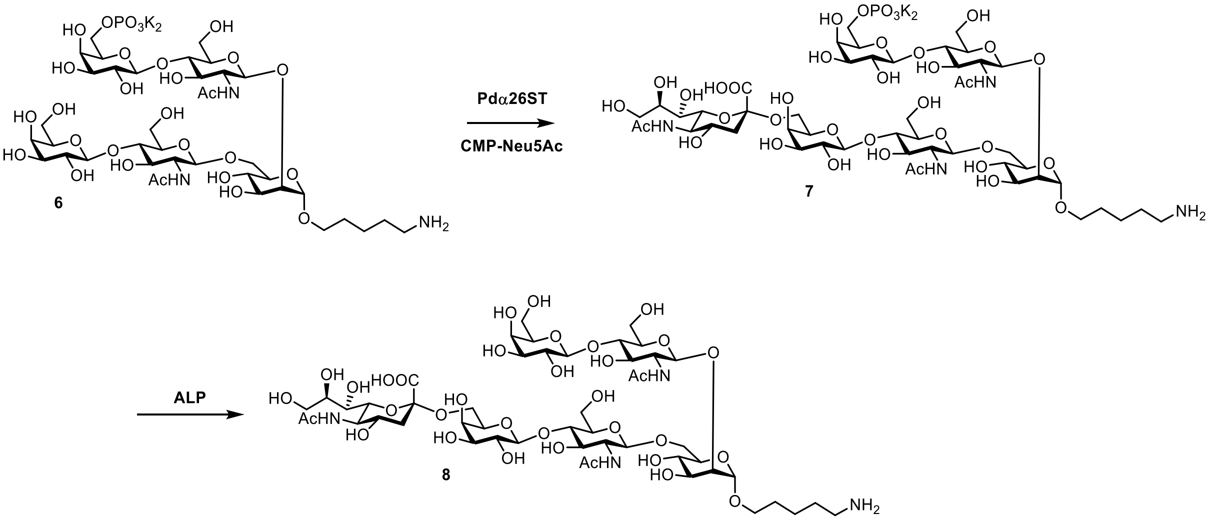

Phosphorylated acceptor **6** (15 µL, 25 mM stock solution, 0.430 mg, 0.375 µmol), CMP-Neu5Ac donor (15 µL, 50 mM stock solution, 2 equiv.), Pdα26ST (30 µL, 10 mU/µL ca.), 5x TRIS buffer (75 µL, 0.5 M, pH 8.0), and H_2O (240 µL) were added into a 1.5 mL Eppendorf tube and placed in an incubator at 37 °C.

Final conditions: 1 mM of acceptor, 2 mM donor, ca. 800 mU/µmol Pdα26ST, 375 µL total volume, 0.375 µmol scale, TRIS 100 mM.

The reaction progress was monitored by RP-HPLC (Method $\mathbf{M_{1A}}$) indicating that a second addition of Pdα26ST (22.5 µL) and CMP-Neu5Ac (15 µL) was needed to drive reaction to completion. Upon completion, the reaction was terminated by thermal denaturation at 90 °C for 15 min. ALP (15 µL, 20 U/µL ca.) was then added and the tube placed in a 37 °C incubator for 24 h. The reaction progress was monitored by reverse phase HPLC (Method $\mathbf{M_{1A}}$).

The reaction crude was centrifuged and the supernatant was directly purified using prep RP-HPLC ($\mathbf{M_{6P}}$). Pure N-glycan **8** was isolated as a white powder (0.470 mg, 97% yield).

Analytical data for **8**:

^{1}H NMR (700 MHz, D_2O) δ 4.52 (dd, *J* = 17.9, 8.0 Hz, 2H), 4.41 (dd, *J* = 17.9, 7.8 Hz, 2H), 4.21 (d, *J* = 9.9 Hz, 1H), 3.99 (s, 1H), 3.94 (t, *J* = 10.2 Hz, 2H), 3.88 (d, *J* = 3.4 Hz, 2H), 3.87 – 3.81 (m, 1H), 3.79 (dd, *J* = 10.3, 5.6 Hz, 2H), 3.76 – 3.72 (m, 4H), 3.71 (d, *J* = 3.9 Hz, 1H), 3.69 – 3.64 (m, 13H), 3.63 – 3.58 (m, 4H), 3.52 – 3.46 (m, 8H), 3.38 (t, *J* = 9.8 Hz, 1H), 2.98 (t, *J* = 7.5 Hz, 2H), 2.62 (dd, *J* = 12.4, 4.6 Hz, 1H), 2.02 (s, 3H), 2.00 (s, 3H), 1.98 (s, 3H), 1.68 – 1.60 (m, 4H), 1.48 – 1.37 (m, 2H).

^{13}C NMR (176 MHz, D_2O) δ 103.2, 102.9, 101.1, 99.3, 96.6, 80.5, 78.2, 76.3, 75.2, 75.0, 74.8, 74.4, 73.9, 73.7, 73.5, 72.1, 72.1, 72.1, 71.5, 71.5, 70.6, 70.0, 69.7, 69.5, 68.4, 68.2, 68.2, 67.4, 67.3, 67.3, 67.3, 63.2, 63.2, 62.6, 62.6, 61.0, 60.0, 60.0, 55.0, 54.7, 51.8, 40.2, 40.2, 39.4, 28.1, 26.6, 22.6, 22.6, 22.4, 22.1

HRMS (QToF): Calcd for $C_{50}H_{85}N_4O_{34}$ $[M]^-$ 1285.5051; found 1285.4999.

^{1}H NMR of 8 (700 MHz, D_2O)

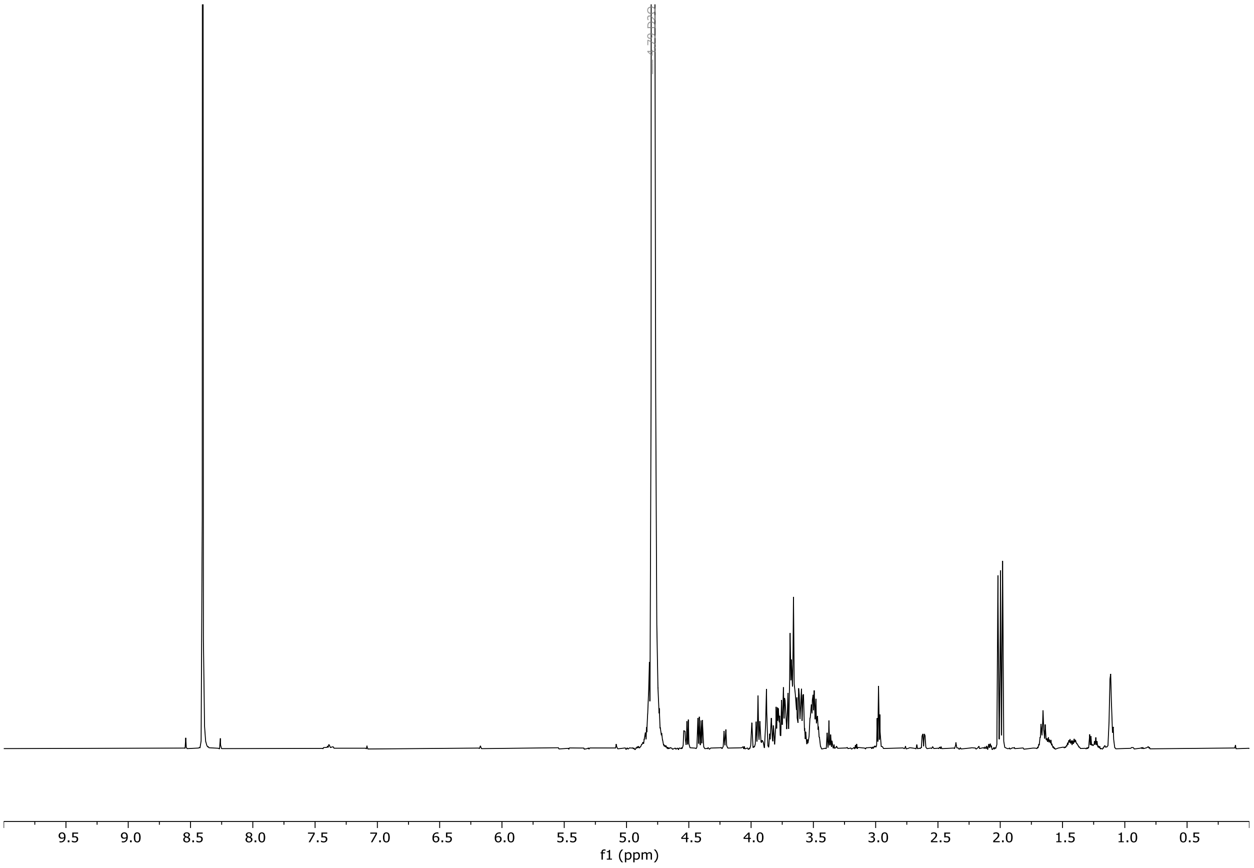

HSQC NMR of 8 (D_2O)

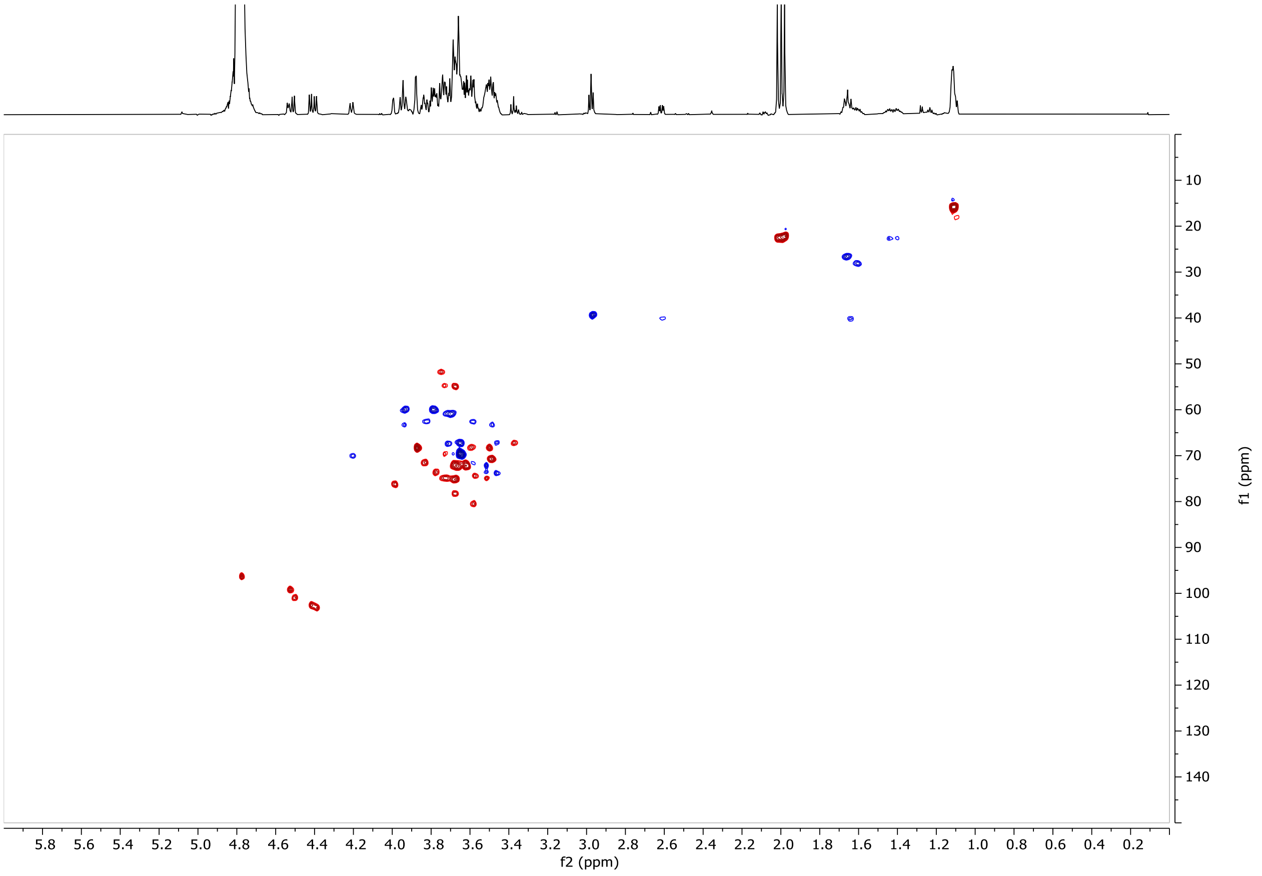

RP-HPLC of 8 (ELSD trace, Method M_{1A}, t_R= 24.83 min)

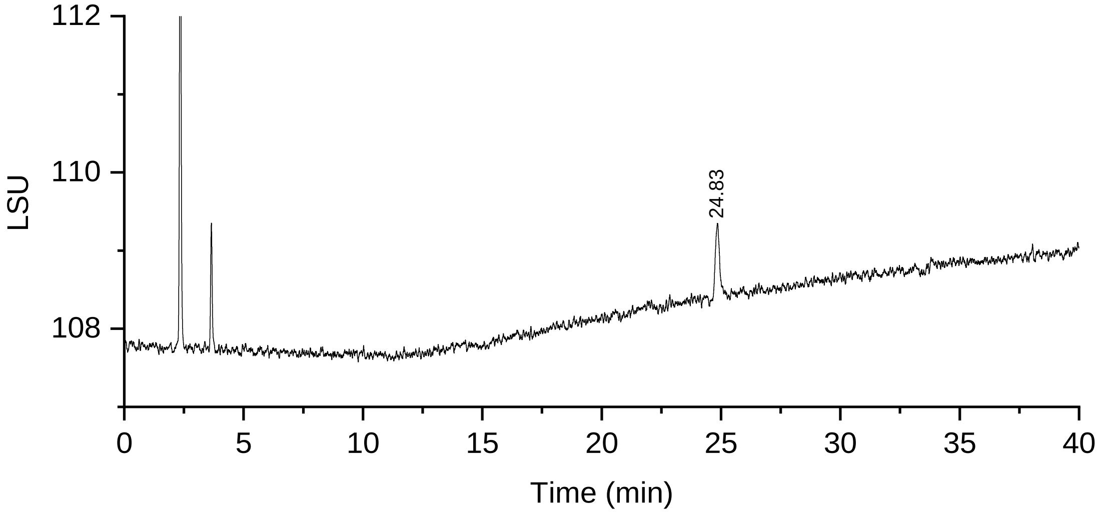

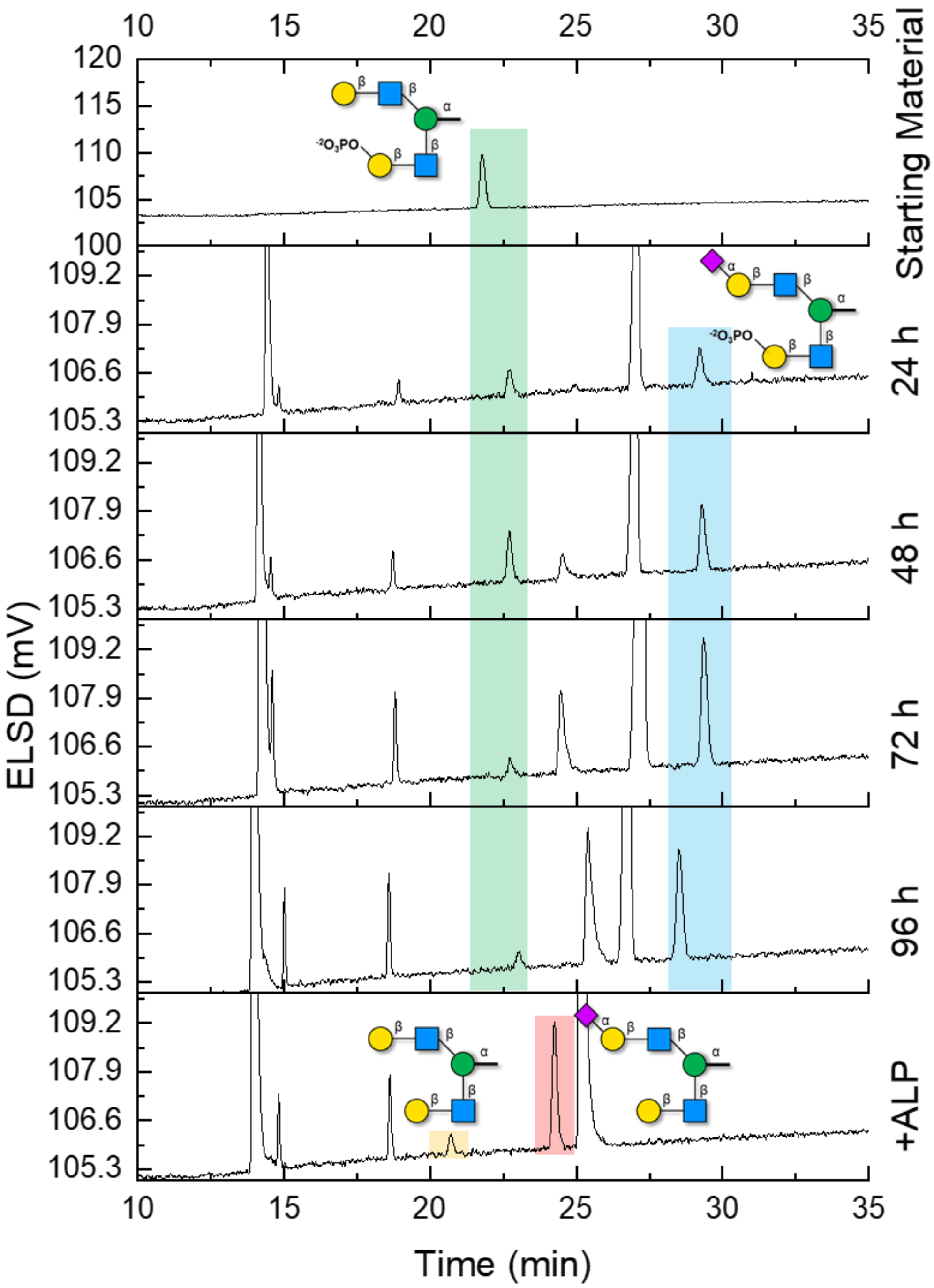

Figure S5. ELSD reverse-phase HPLC traces monitoring the steps of the one-pot sialylation/dephosphorylation of acceptor **6** for the synthesis of monosialylated **8**. Peaks were determined by inline mass spectrometer.

5.3 Nonselective sialylation

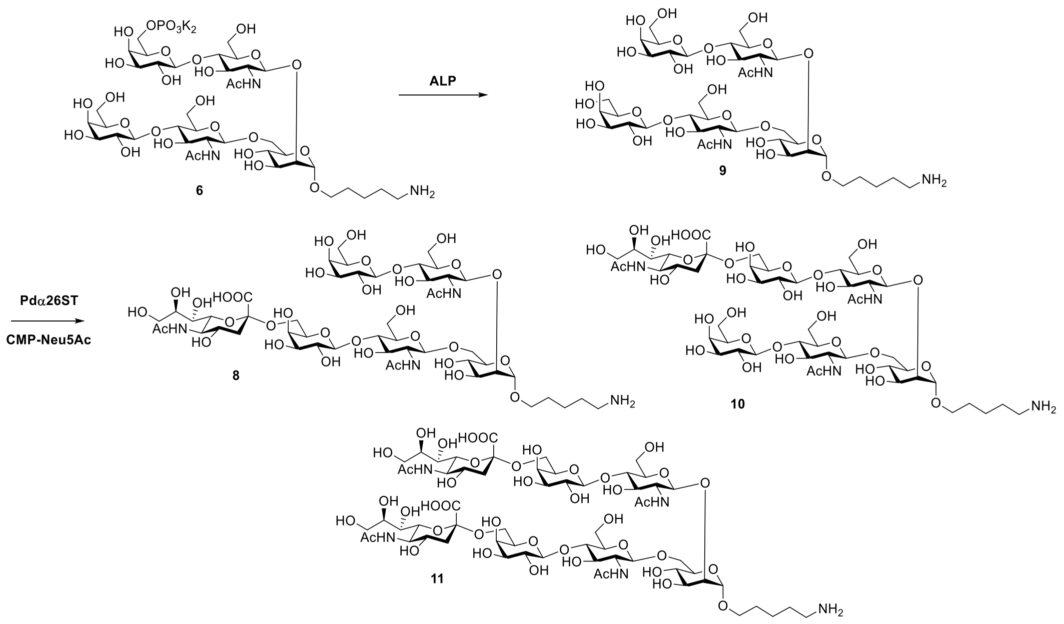

Phosphorylated acceptor **6** (1 µL, 25 mM stock solution), ALP (0.5 µL, 20 U/µL ca.), 5X TRIS buffer (5 µL, 0.5 M, pH 8.0), and MilliQ water (18.5 µL) were added into a 0.5 mL PCR tube and incubated at 37 °C for 24 h. Dephosphorylation was confirmed by RP-HPLC (Method $\mathbf{M_{1A}}$) and the ALP was denatured by exposing the tube to 90 °C for 3 min. Upon completion, Pdα26ST (2 µL, 10 mU/µL ca.) and CMP-Neu5Ac donor (2 µL, 50 mM stock solution, 4 equiv.) were added to the tube and the reaction was incubated at 37 °C. the reaction progress was monitored by RP-HPLC (Method $\mathbf{M_{1A}}$) and compared to that of the selective method (Supplementary Figure S3).

Final conditions: 1 mM of acceptor, 2 mM donor, ca. 800 mU/µmol Pdα26ST, 400 U/µmol ALP, 25 µL total volume, 0.025 µmol scale, TRIS 100 mM

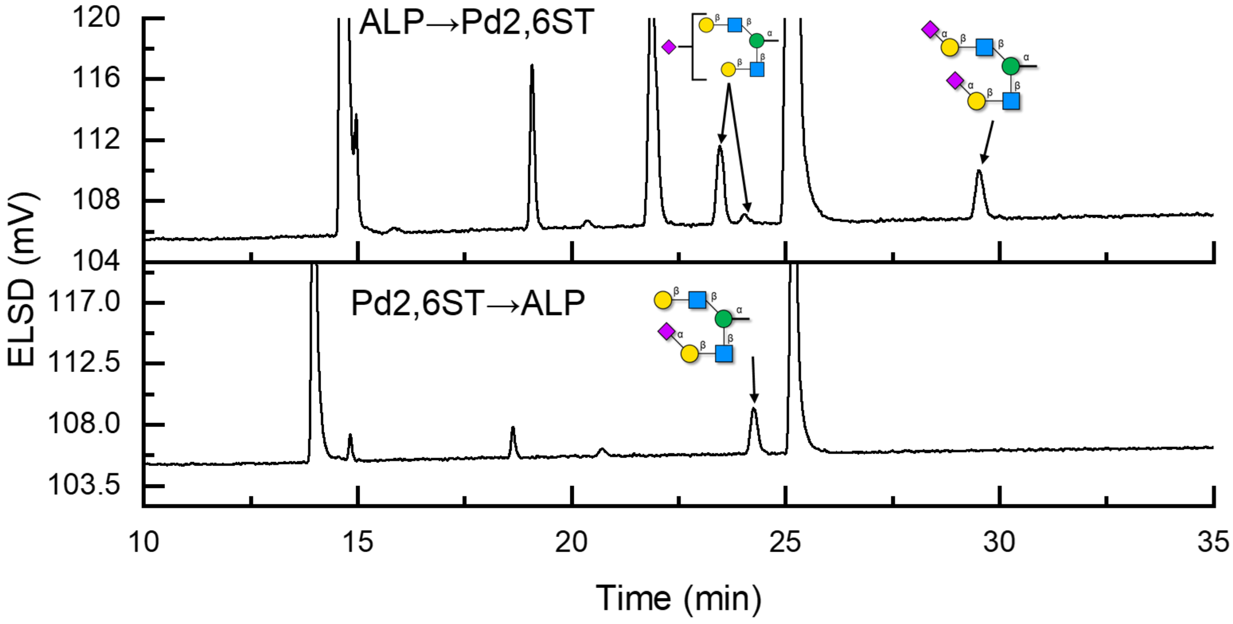

`

Figure S6. Comparison of ELSD reverse-phase HPLC traces monitoring of the one-pot sialylation/dephosphorylation protocol to the inversed protocol. Peaks were determined by inline mass spectrometer.

6 ALP mediated precipitation of well-defined cellulose chains

6.1 Experimental

6.1.1 General protocol

Phosphorylated cellulose, ALP (20 U/µL), 10X DEA buffer were added to a 0.5 mL PCR tube and diluted to the desired concentration by addition of MilliQ water. The reaction was then incubated at the desired temperature for 24 h and monitored by RP-HPLC and through visual inspection of precipitate formation. Upon formation of precipitate, the reaction mixture was diluted with 40 µL of MilliQ water and centrifuged. The supernatant was removed leaving ~5 µL and the precipitate was resuspended in 40 µL of MilliQ water. The centrifugation and washing was repeated three times in total. Upon washing, the precipitate was resuspended in 40 µL of MilliQ water and used for further analysis. These samples were than further diluted 10x or 100x for imaging.

6.1.2 TEM imaging

Transmission electron microscopy was conducted using a JEOL JEM F200 (Jeol, Japan) (S)TEM equipped with a field emission gun and a TVIPS TemCam-F216 (2k x 2k) camera. The microscope was operated at 80 kV; a condenser aperture with a diameter of 200 µm was used.

For specimen preparation, 5 µL of aqueous suspension of samples were deposited on glow-discharged carbon-coated copper grids (Plano GmbH, Germany). After approximately one minute, excess liquid was carefully blotted away with filter paper and the specimen was allowed to dry for 20 minutes.

6.1.3 AFM imaging

Atomic force microscopy was performed with a JPK NanoWizard 4 AFM in tapping mode (AC mode) using Arrow NCR tip (42 N/m, 285 kHz, Nano World). Drops of aqueous suspensions were deposited on freshly cleaved mica and dried at room temperature and analyzed the same day. AFM images were collected with 1024 x 1024 or 512 x 512 pixels/frame and analyzed with the JPK Data Processing software.

6.2 **Monophosphate octamer**

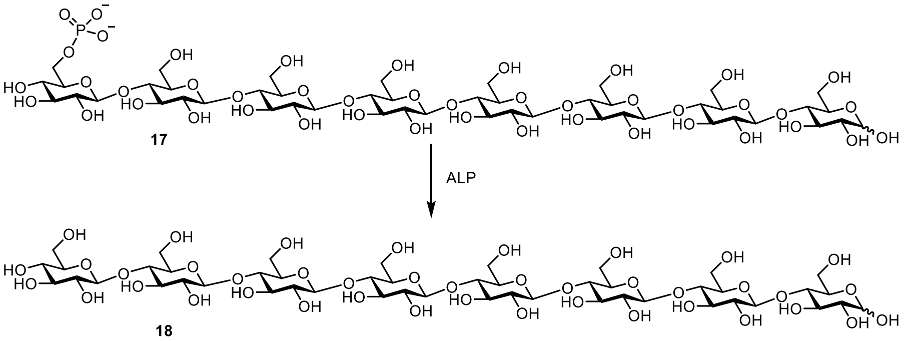

17

18

6.2.1 Screening the conditional effects on platelet morphology

Reactions were set up following the general ALP dephosphorylation procedure (50 nmol substrate) at varied substrate concentrations and enzyme amounts at 37 °C or otherwise defined temperature. The reaction progress was monitored via visual inspection for precipitation at 24 h. The reaction at 4 °C was allowed to react for 48 h.

6.2.2 Large scale dephosphorylation

The reaction was set up following the general ALP deposphorylation conditions (substrate = 0.5 mg, 358 nmol, 2 mM; scale = 180 µL, enzyme = 1600 U/µmol) at 37 °C. The reaction progress was monitored via visual inspection for precipitation at 24 h. Upon completion, the centrifugation/wash procedure was followed and the sample was lyophilized to dryness resulting in **X** as a white solid (0.17 mg, 36% yield).

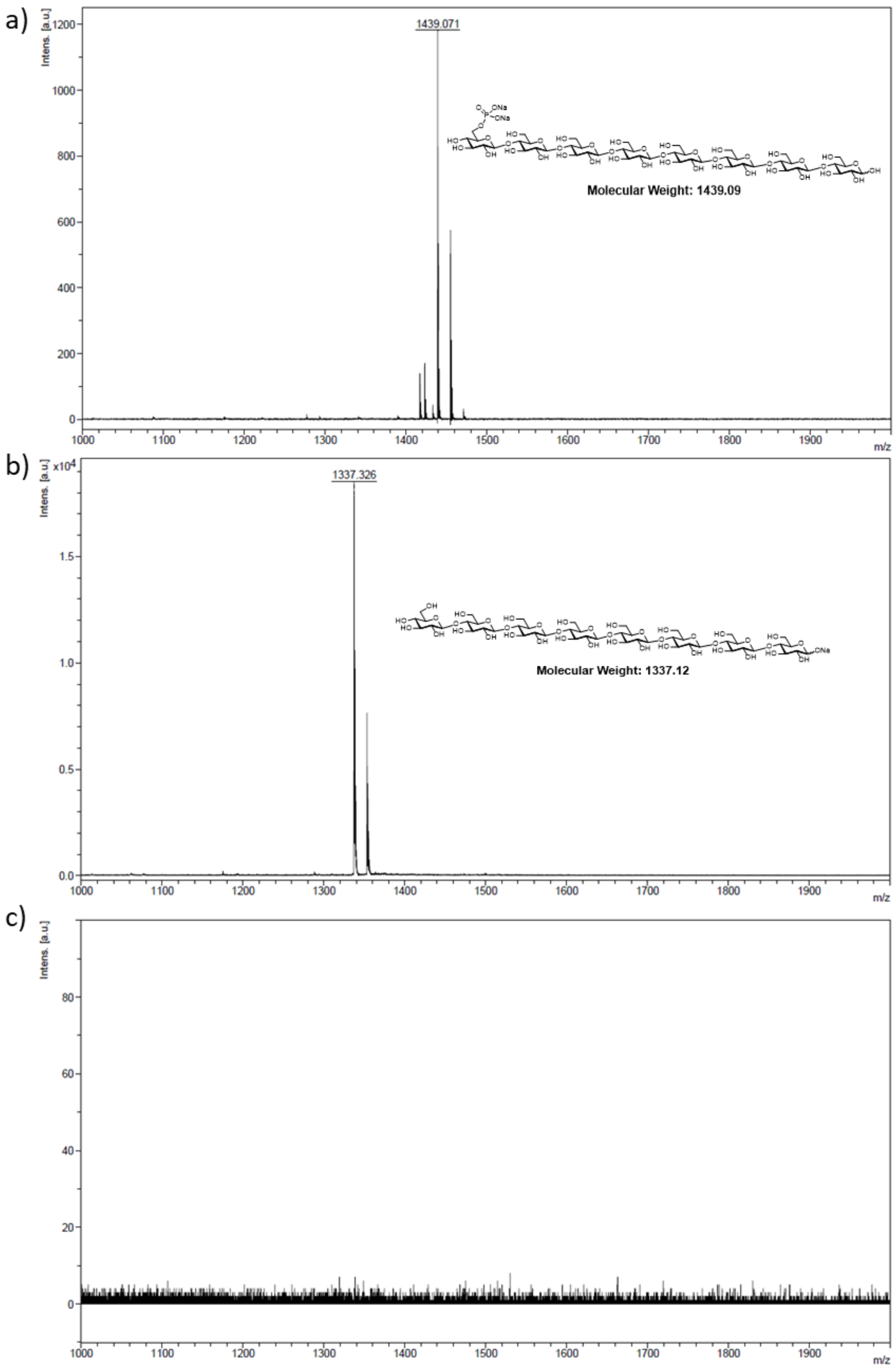

Figure S7. MALDI-ToF of cellulose octamer **17** before (a-postive mode) and after (**18**, b-positive mode, c-negative mode) exposure to ALP.

6.2.3 Imaging of phosphated octamer **17** -before ALP

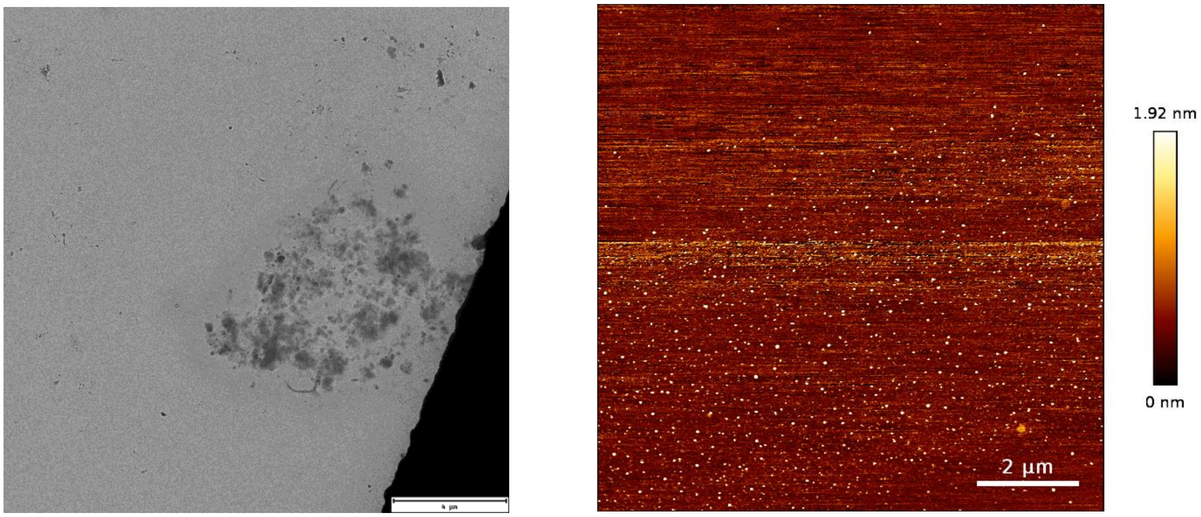

Figure S8. Representative AFM and TEM images of phosphorylated cellulose octamer **17** prior to treatment with ALP.

6.2.4 Imaging comparison

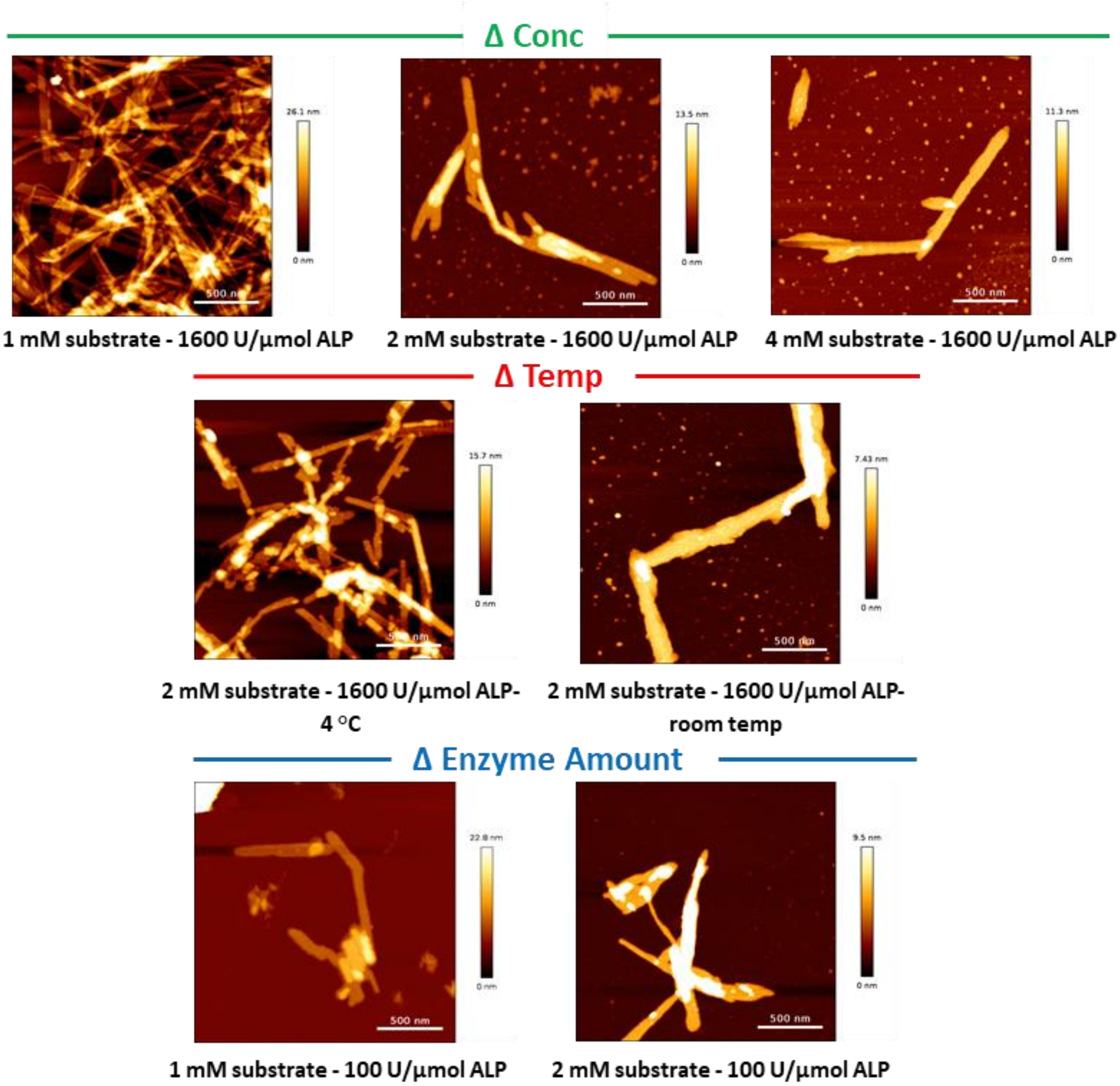

Figure S9. Representative AFM images of cellulose octamer obtained as a precipitate after dephosphorylation of the monophosphate octamer under different reaction conditions (substrate concentration, reaction temperature, amount of enzyme). Sample diluted 10x and drop casted on freshly cleaved mica.

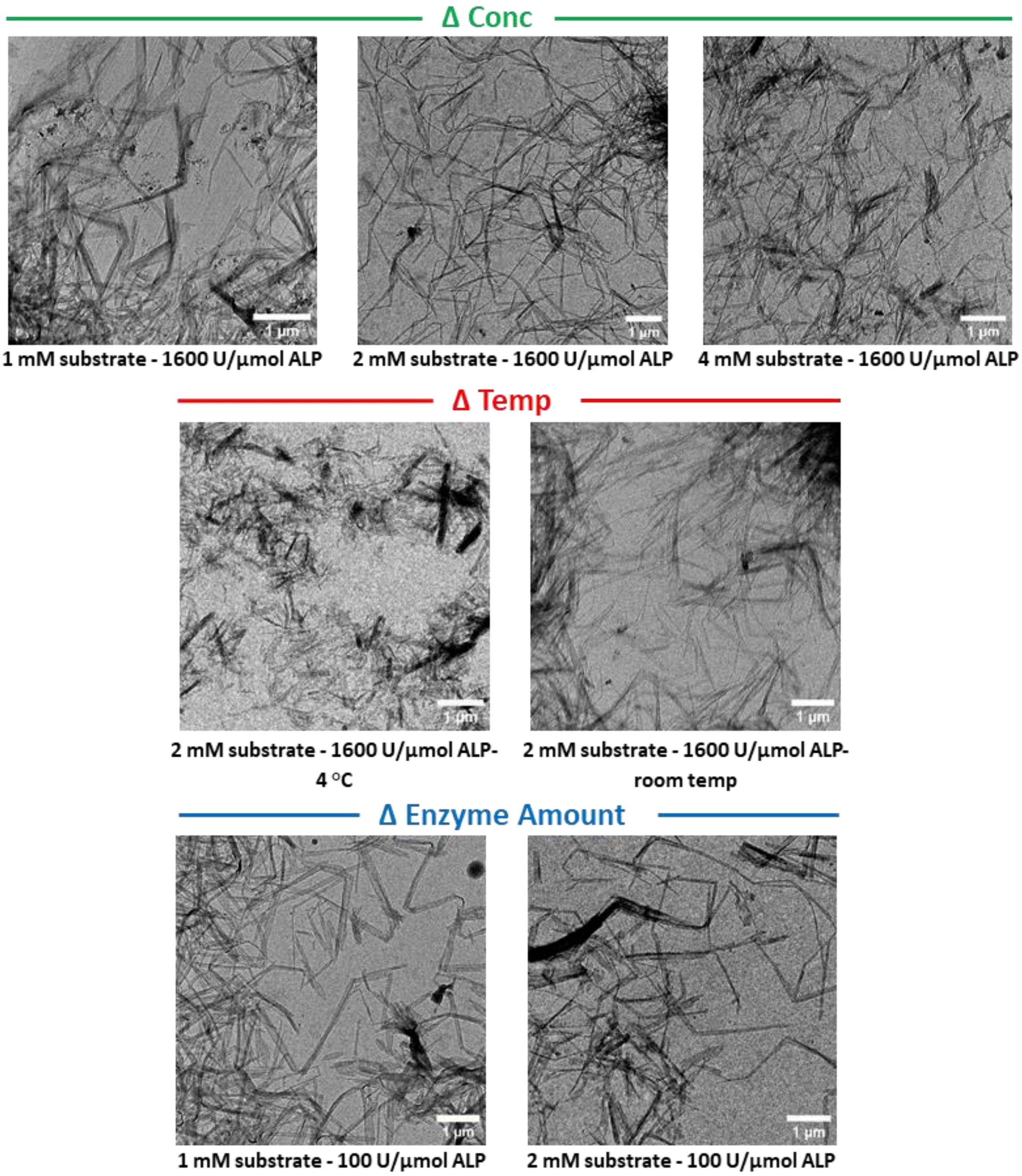

Figure S10. Representative TEM images of cellulose octamer obtained as a precipitate after dephosphorylation of the monophosphate octamer under different reaction conditions (substrate concentration, reaction temperature, amount of enzyme). Sample diluted 10x and drop casted on freshly glow discharged TEM copper grid.

6.2.5 Platelet height analysis

6.2.5.1 1 mM substrate - 1600 U/µmol ALP

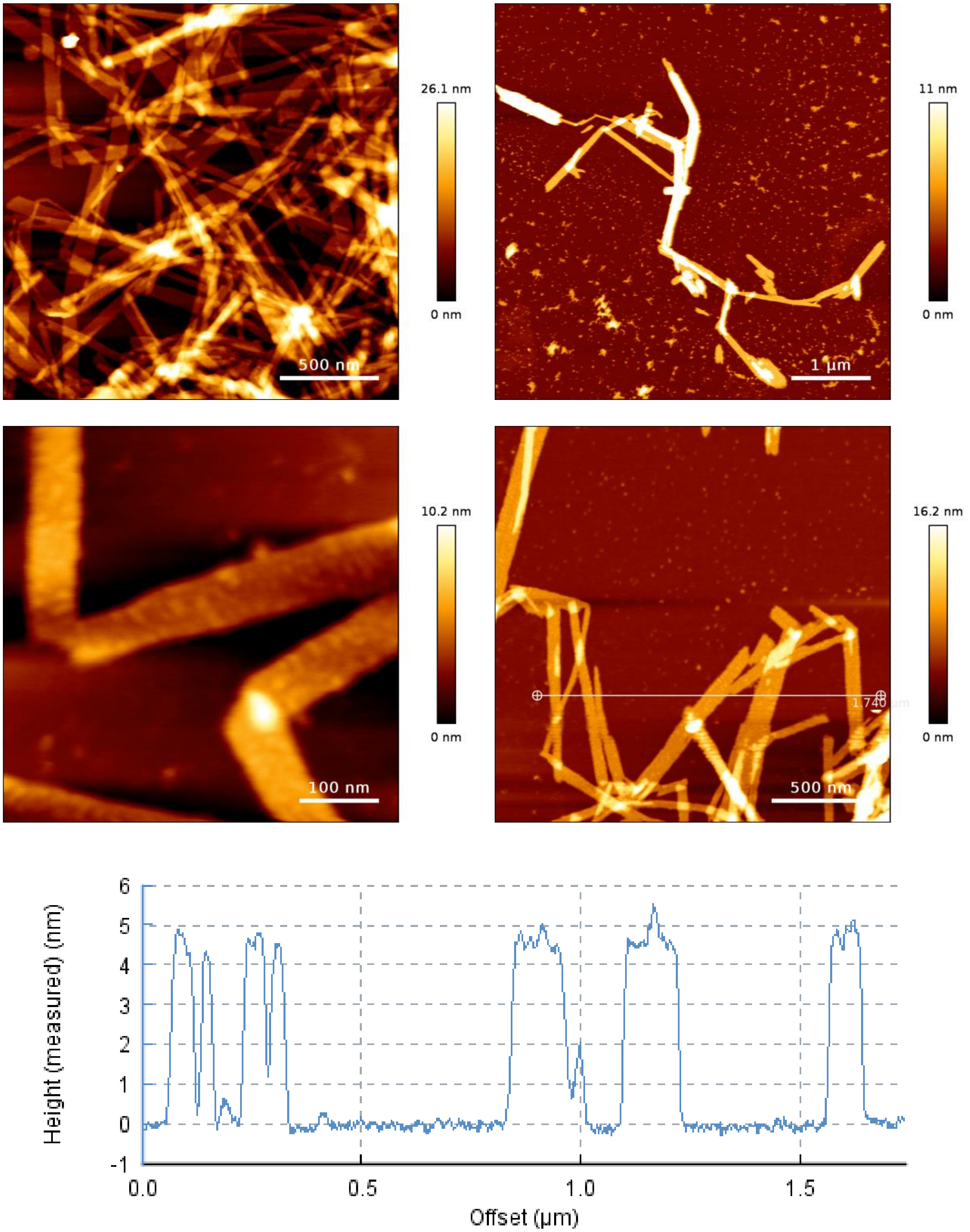

Figure S11. Representative AFM images and height image of cellulose octamer obtained after dephosphorylation of the monophosphate octamer (1 mM), using 1600 U/µmol ALP, temperature 37 ˚C.

6.2.5.2 2 mM substrate - 1600 U/µmol ALP

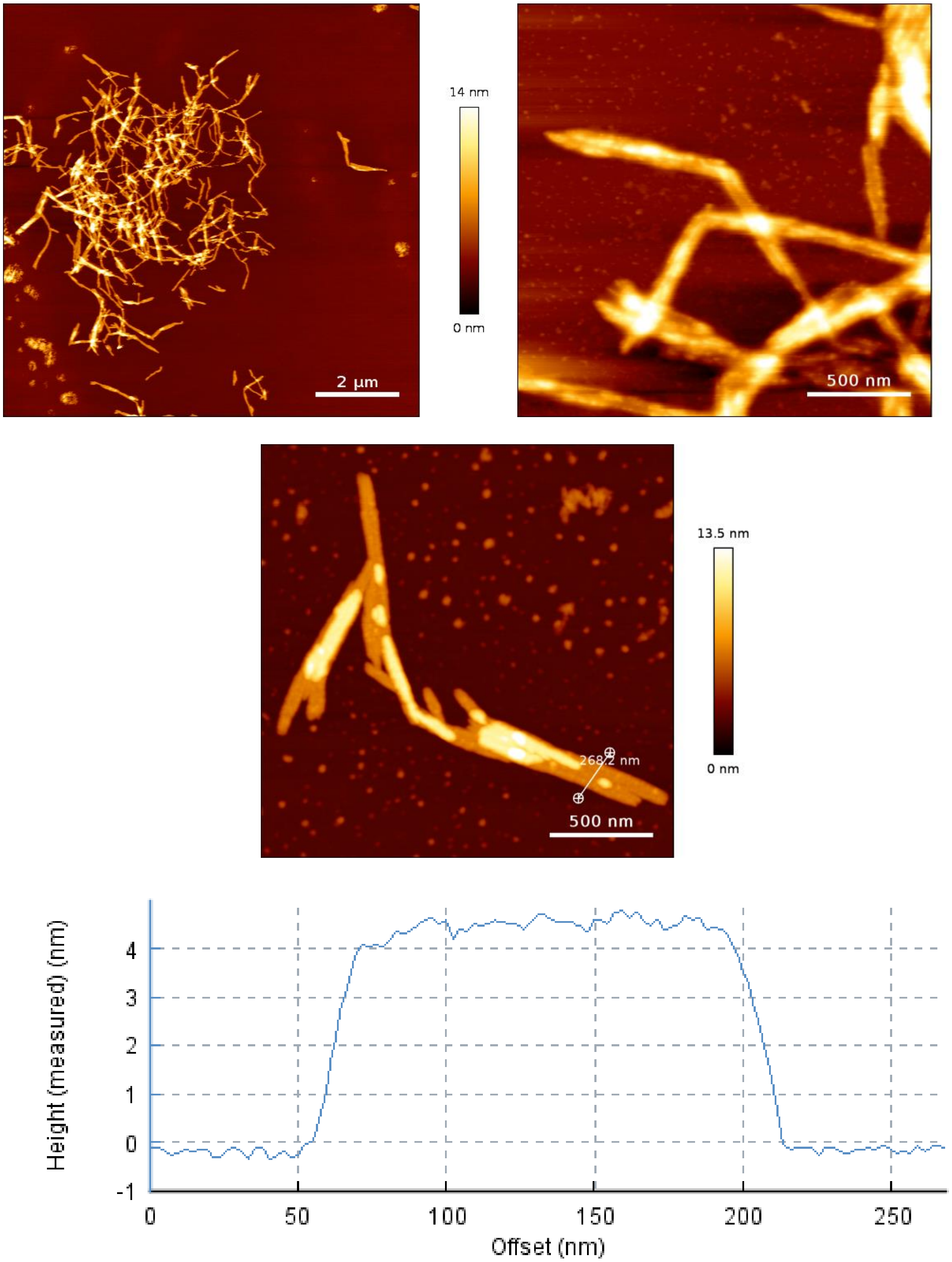

Figure S12. Representative AFM images and height image of cellulose octamer obtained after dephosphorylation of the monophosphate octamer (2 mM), using 1600 U/µmol ALP, temperature 37 ˚C.

6.2.5.3 4 mM substrate - 1600 U/µmol ALP

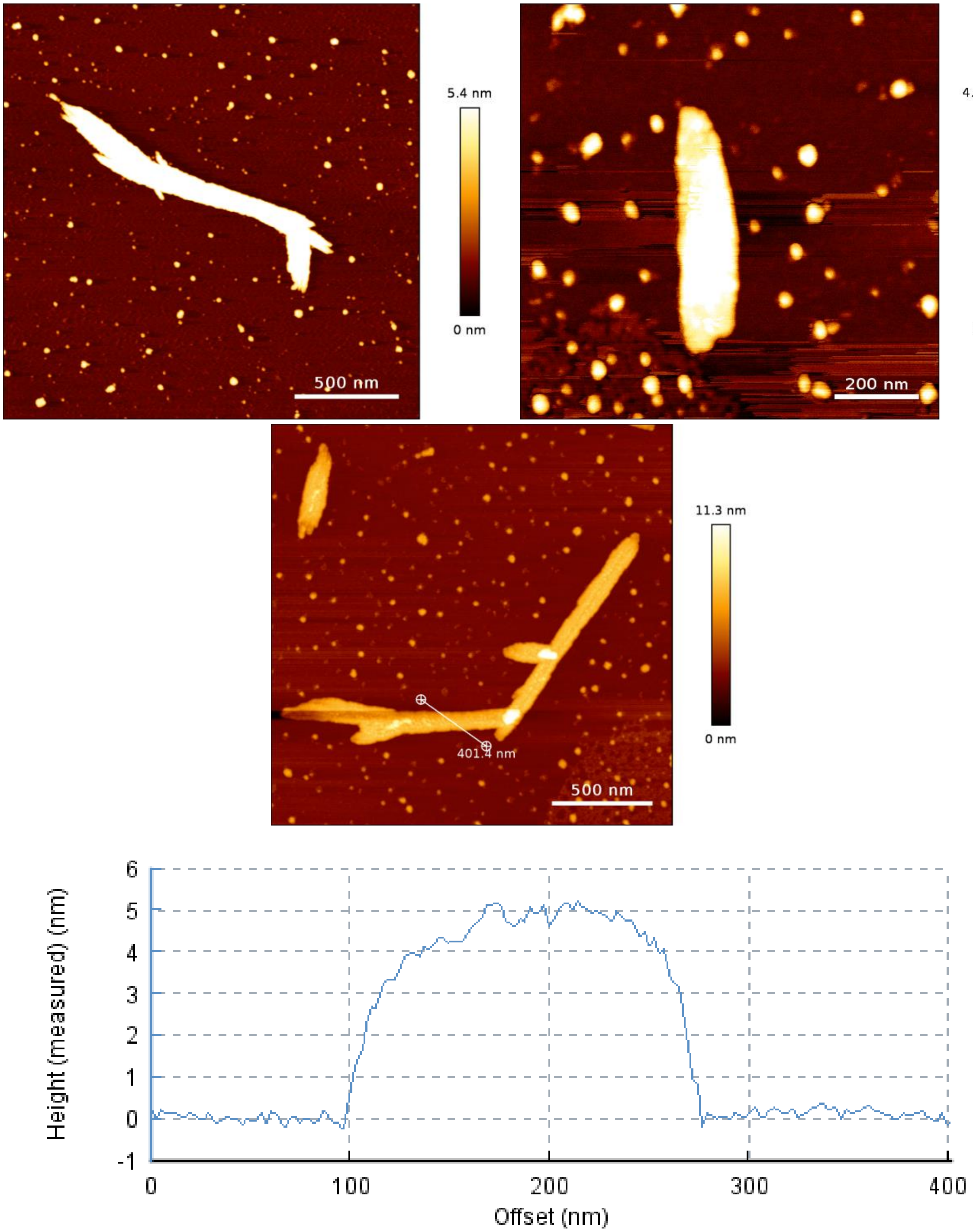

Figure S13. Representative AFM images and height image of cellulose octamer obtained after dephosphorylation of the monophosphate octamer (4 mM), using 1600 U/µmol ALP, temperature 37 ˚C.

6.2.5.4 2 mM substrate - 1600 U/µmol ALP- 4 °C

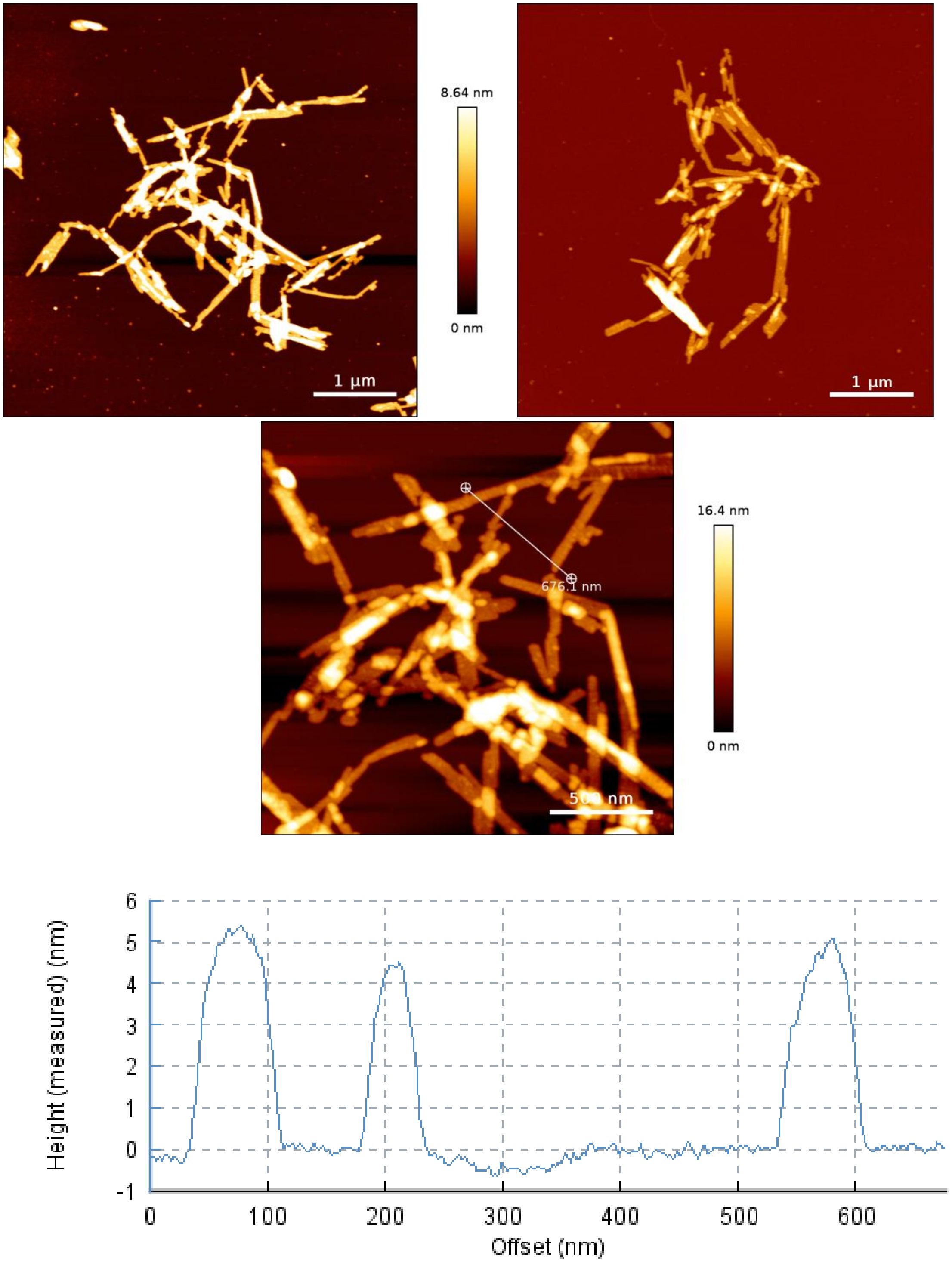

Figure S14. Representative AFM images and height image of cellulose octamer obtained after dephosphorylation of the monophosphate octamer (2 mM), using 1600 U/µmol ALP, temperature 4 ˚C.

6.2.5.5 2 mM substrate - 1600 U/µmol ALP- room temp

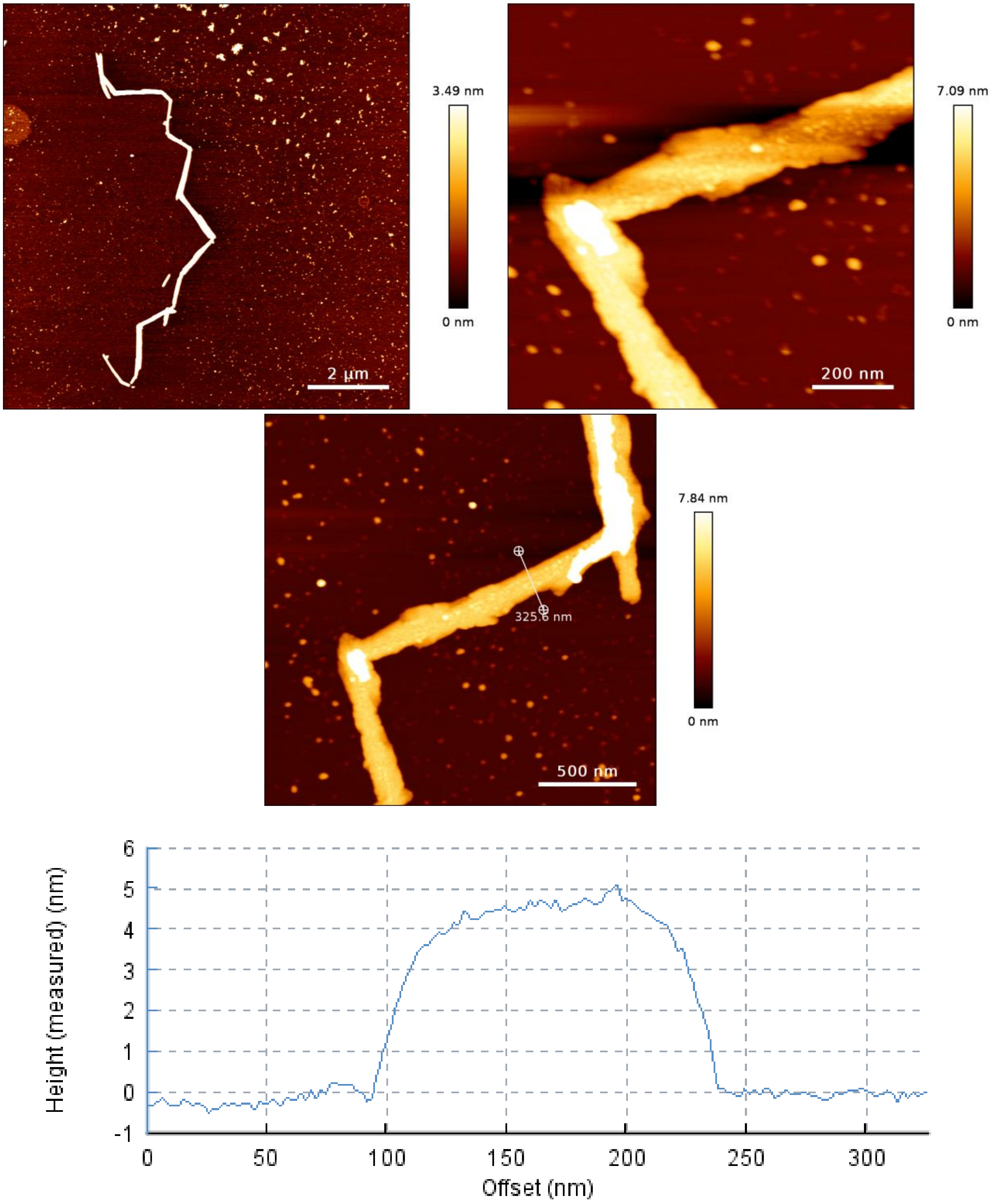

Figure S15. Representative AFM images and height image of cellulose octamer obtained after dephosphorylation of the monophosphate octamer (2 mM), using 1600 U/µmol ALP, room temperature.

6.2.5.6 1 mM substrate - 100 U/µmol ALP

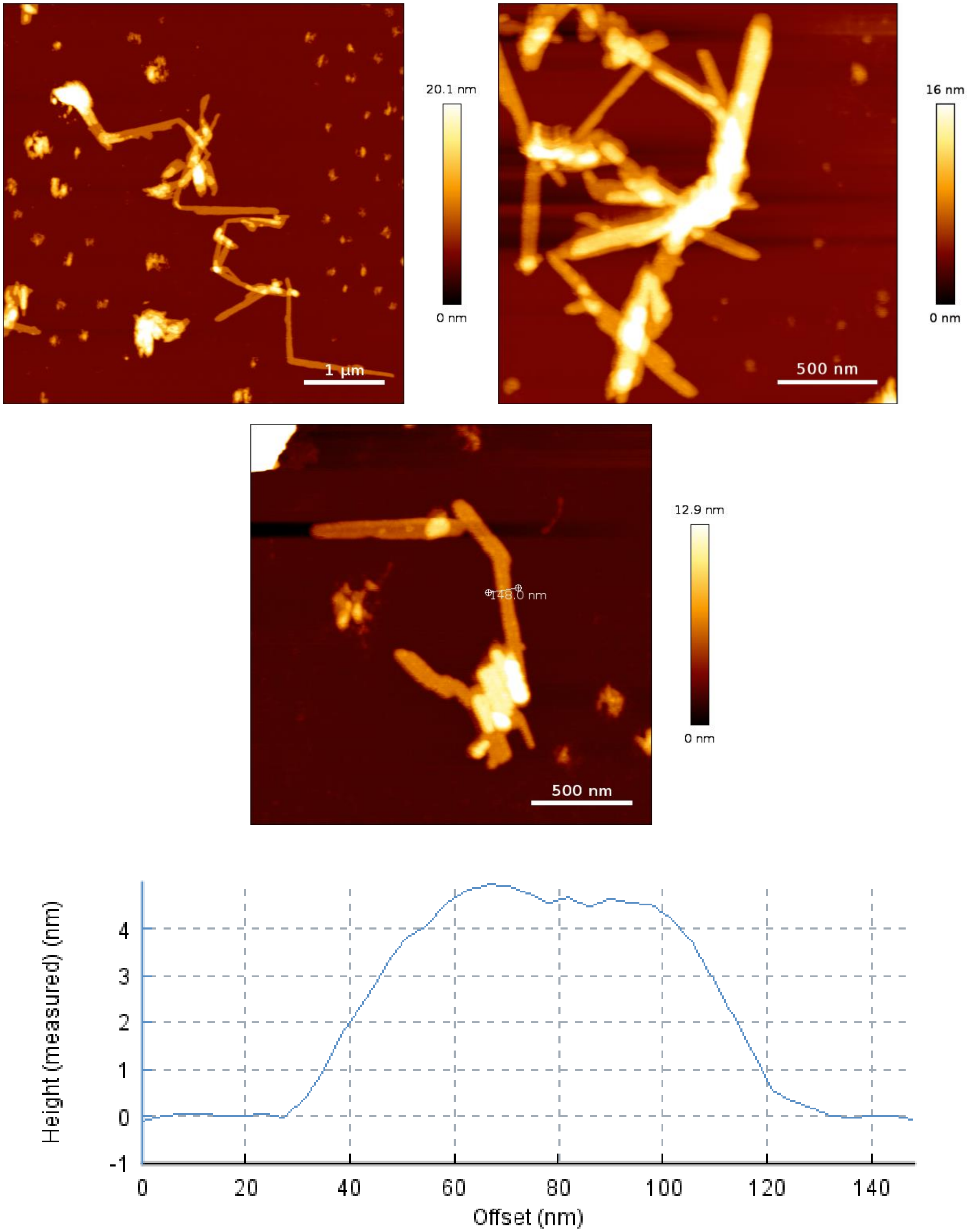

Figure S16. Representative AFM images and height image of cellulose octamer obtained after dephosphorylation of the monophosphate octamer (1 mM), using 100 U/µmol ALP, temperature 37 ˚C.

6.2.5.7 2 mM substrate - 100 U/µmol ALP

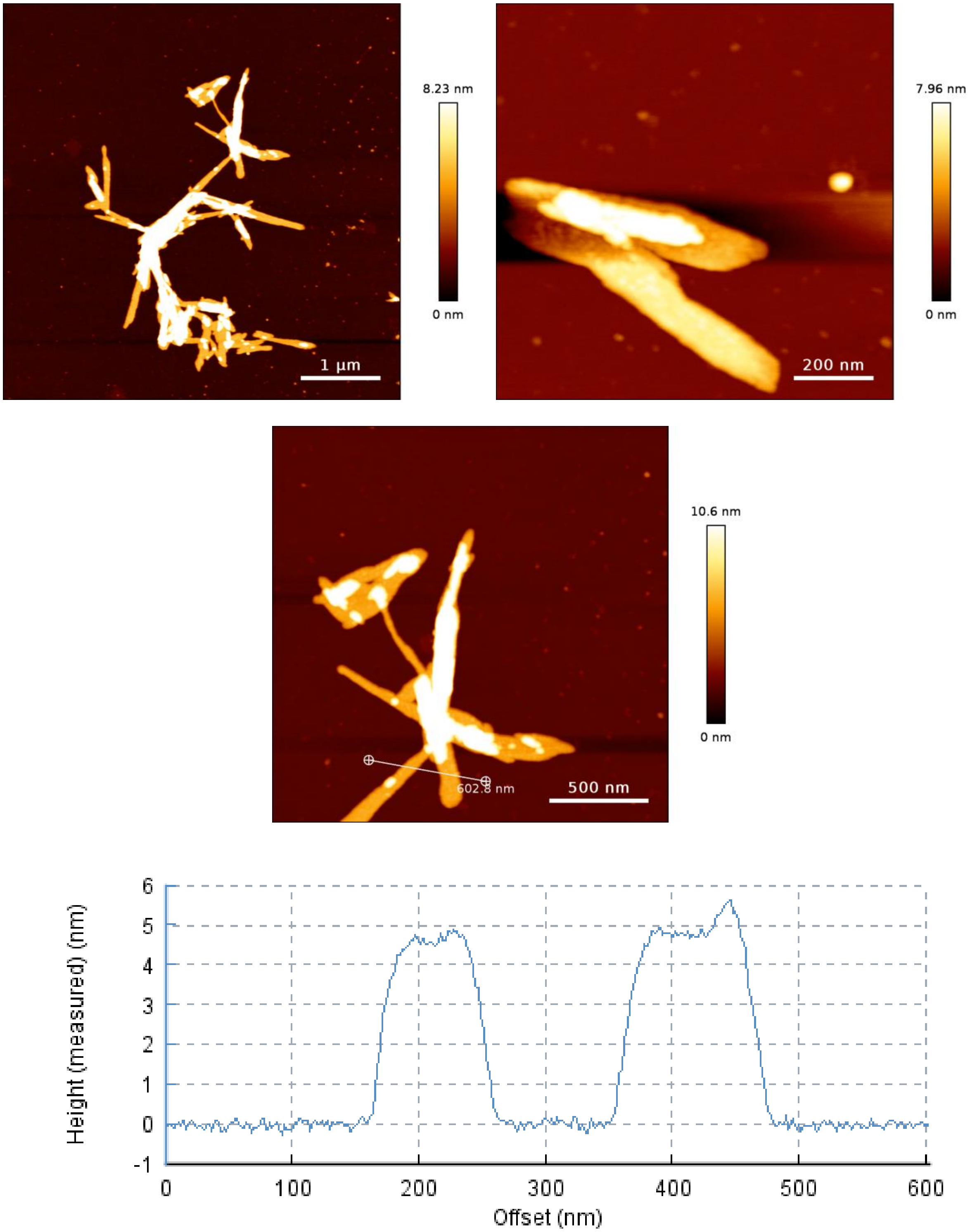

Figure S17. Representative AFM images and height image of cellulose octamer obtained after dephosphorylation of the monophosphate octamer (2 mM), using 1600 U/µmol ALP, temperature 37 ˚C.

6.3 Diphosphate hexamer screening

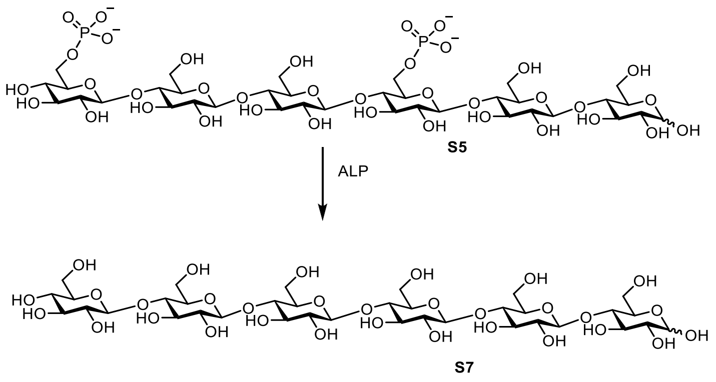

Reactions were set up following the general ALP deposphorylation procedure at 25 µL scale (25 nmol substrate, 50 mM stock solution) at 37 °C. Reaction progress was monitored by RP-HPLC (Method $\mathbf{M_{3A}}$).

*10 mM reactions were performed on 10 µL scale with 50 nmol substrate (100 mM stock solution).

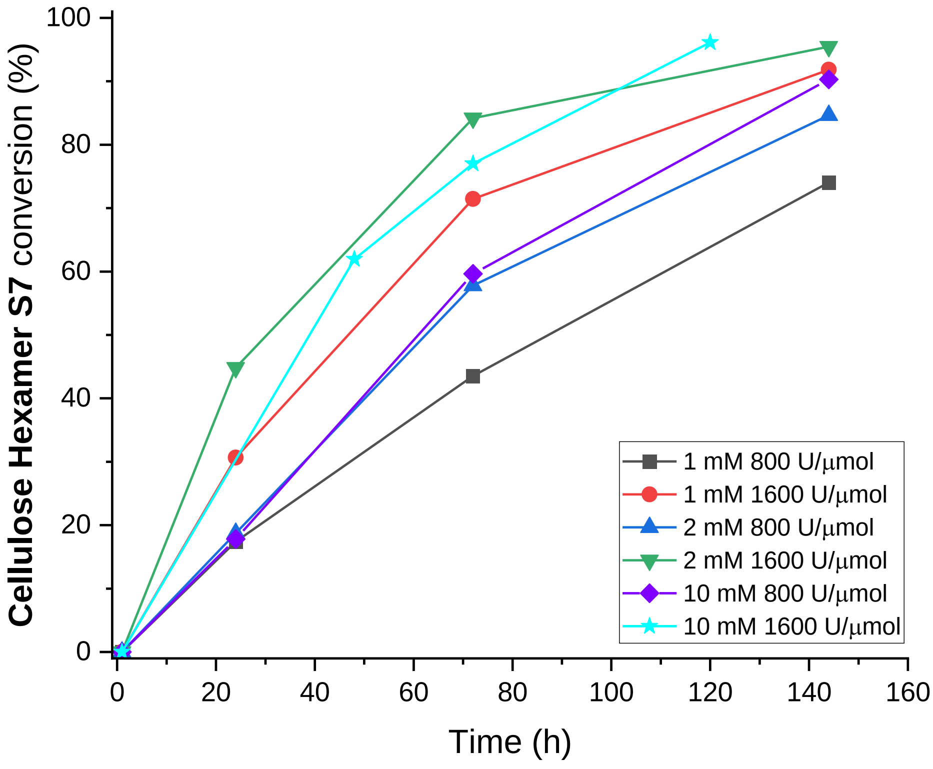

Figure S18. Optimization conditions for phosphate removal of **S5.**

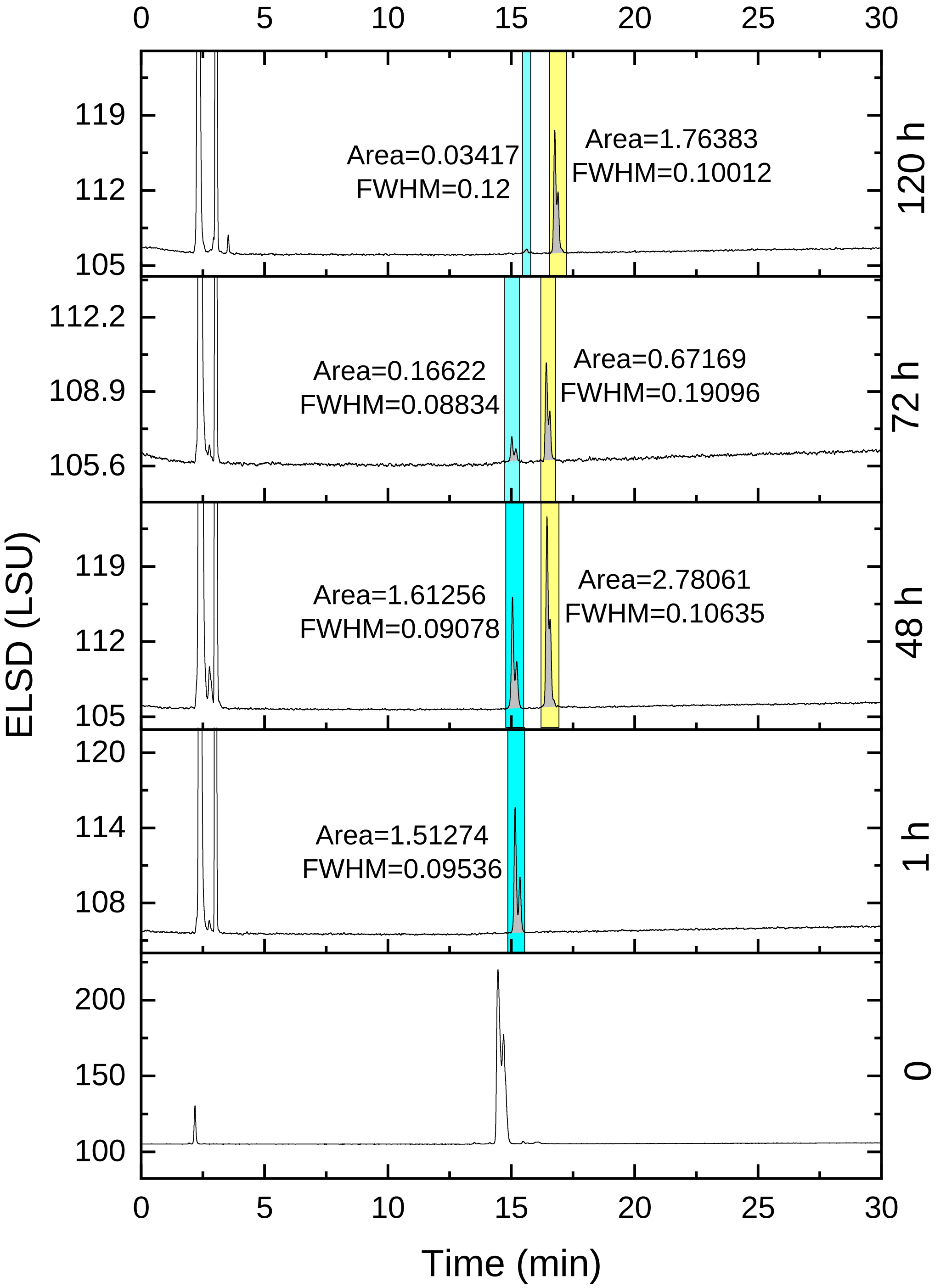

Figure S19. Representative RP-HPLC ELSD trace for monitoring the optimization for phosphate removed in **S5.**

6.4 Triphosphate dodecamer

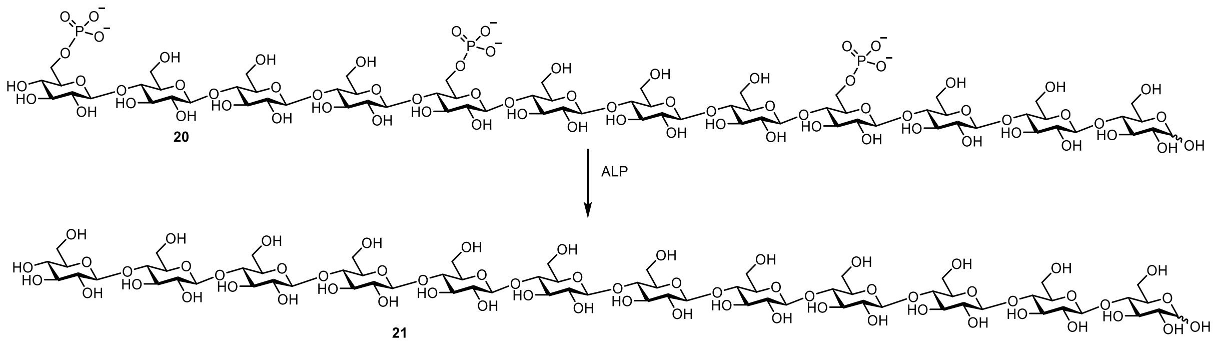

Reactions were set up following the general ALP deposphorylation procedure at 25 µL scale (50 nmol substrate, 2 mM substrate, 1600 U/µmol ALP) at 37 °C. Reaction progress was monitored by RP-HPLC (Method **M$_{3A}$**).

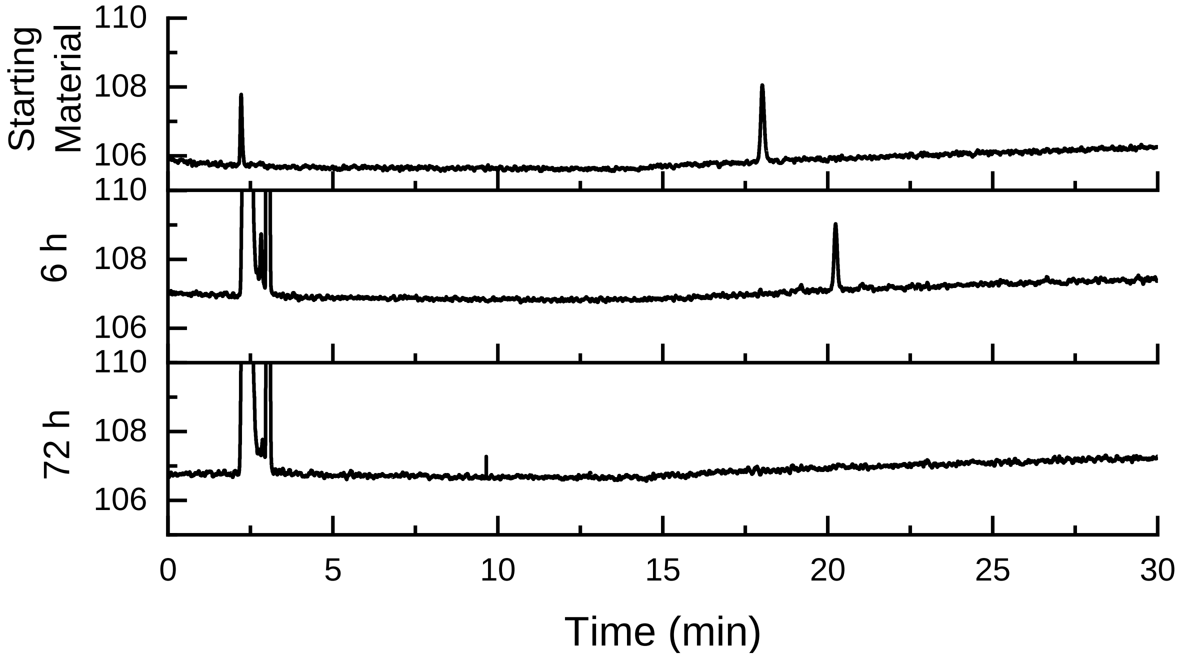

Figure S20. RP-HPLC ELSD trace for monitoring dephosphorylation of **20**. The disappearance of the peak in the trace at 72 h is due to complete precipitation of **21**.

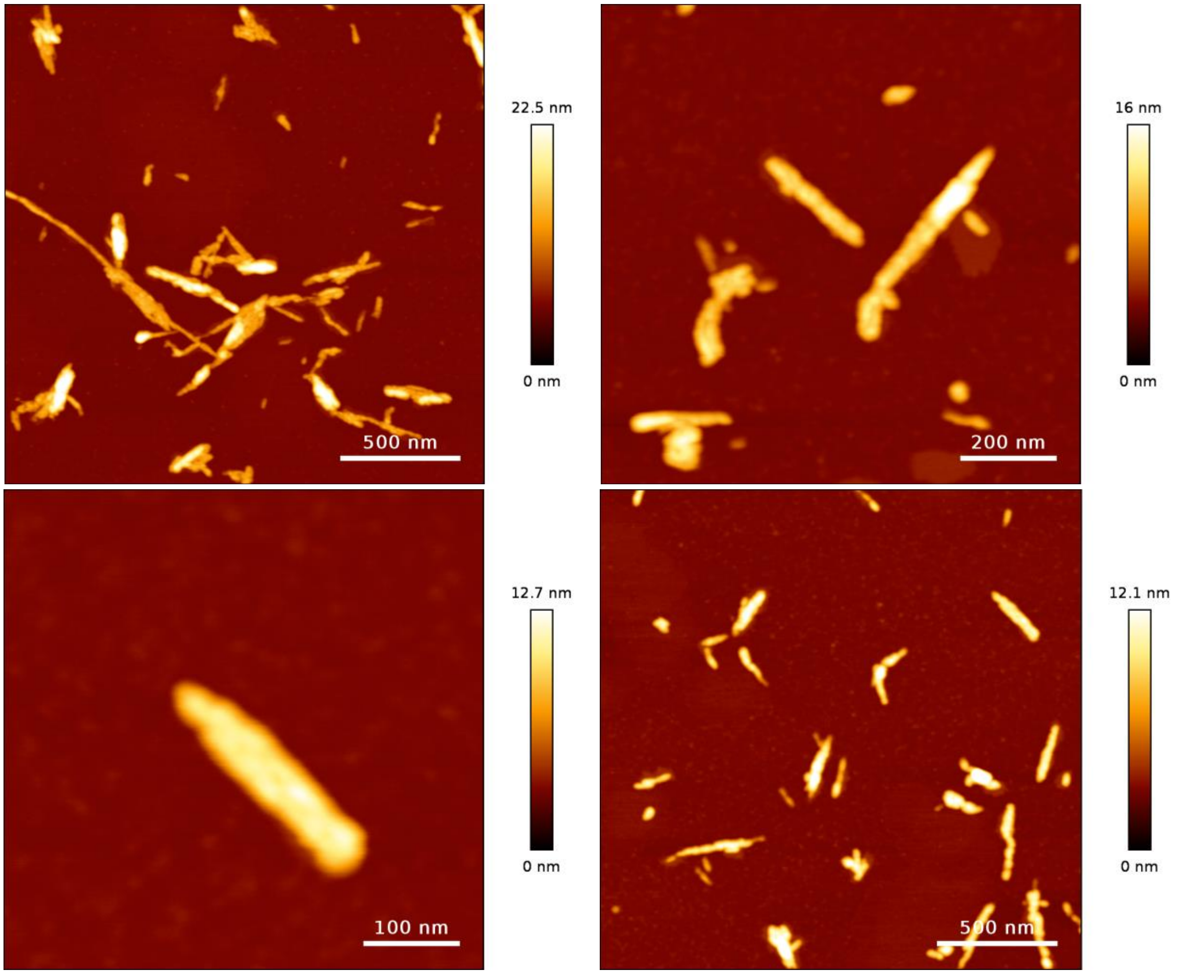

Figure S21. Representative AFM images of cellulose dodecamer **21** obtained as a precipitate after dephosphorylation of the triphosphate dodecamer (2 mM substrate, 1600 U/µmol ALP, temperature 37 °C). Sample diluted 100x and drop casted on freshly cleaved mica.

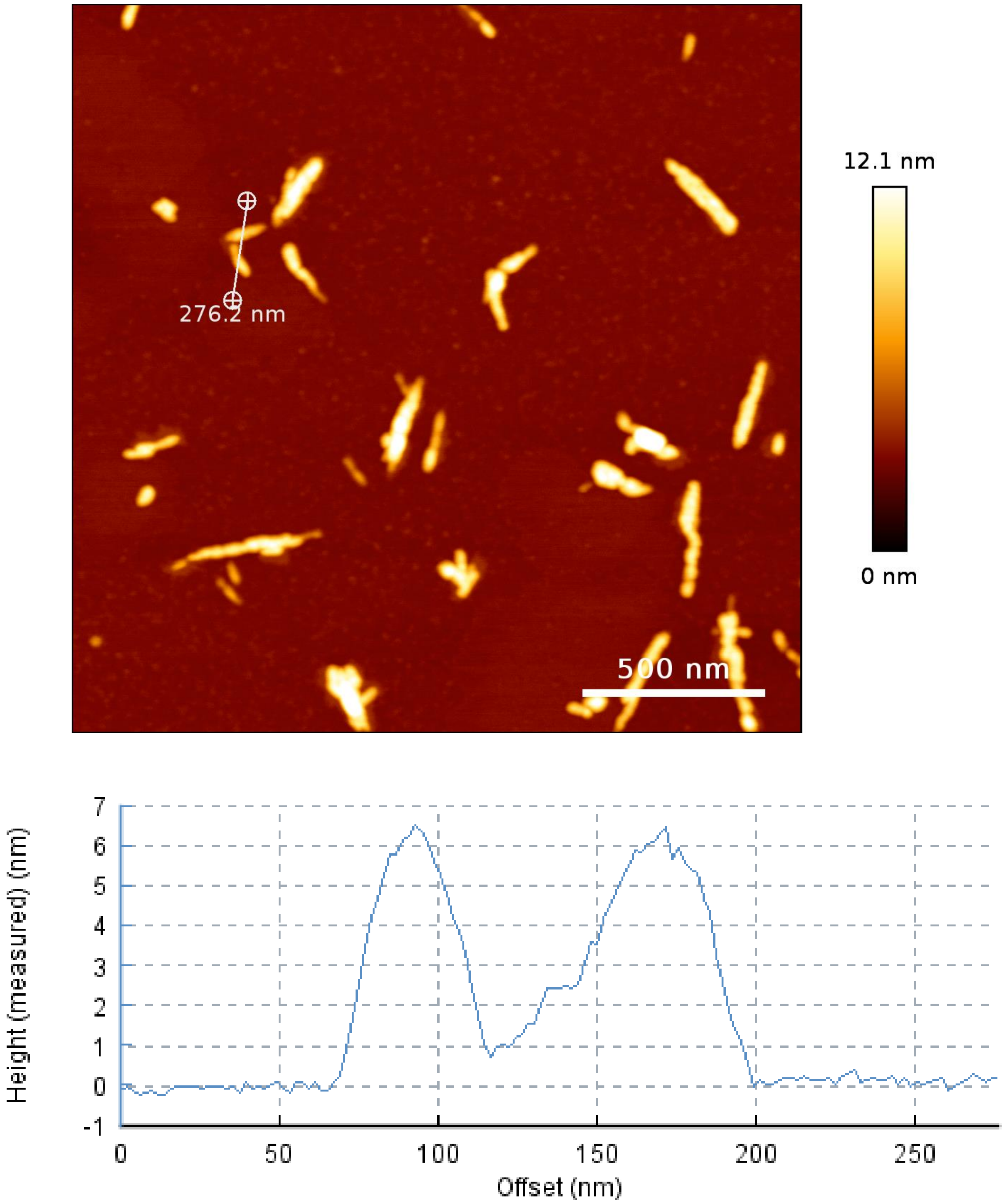

Figure S22. AFM height image of cellulose dodecamer **21** obtained after dephosphorylation of the triphosphate dodecamer (2 mM), using 1600 U/µmol ALP, temperature 37 ˚C.

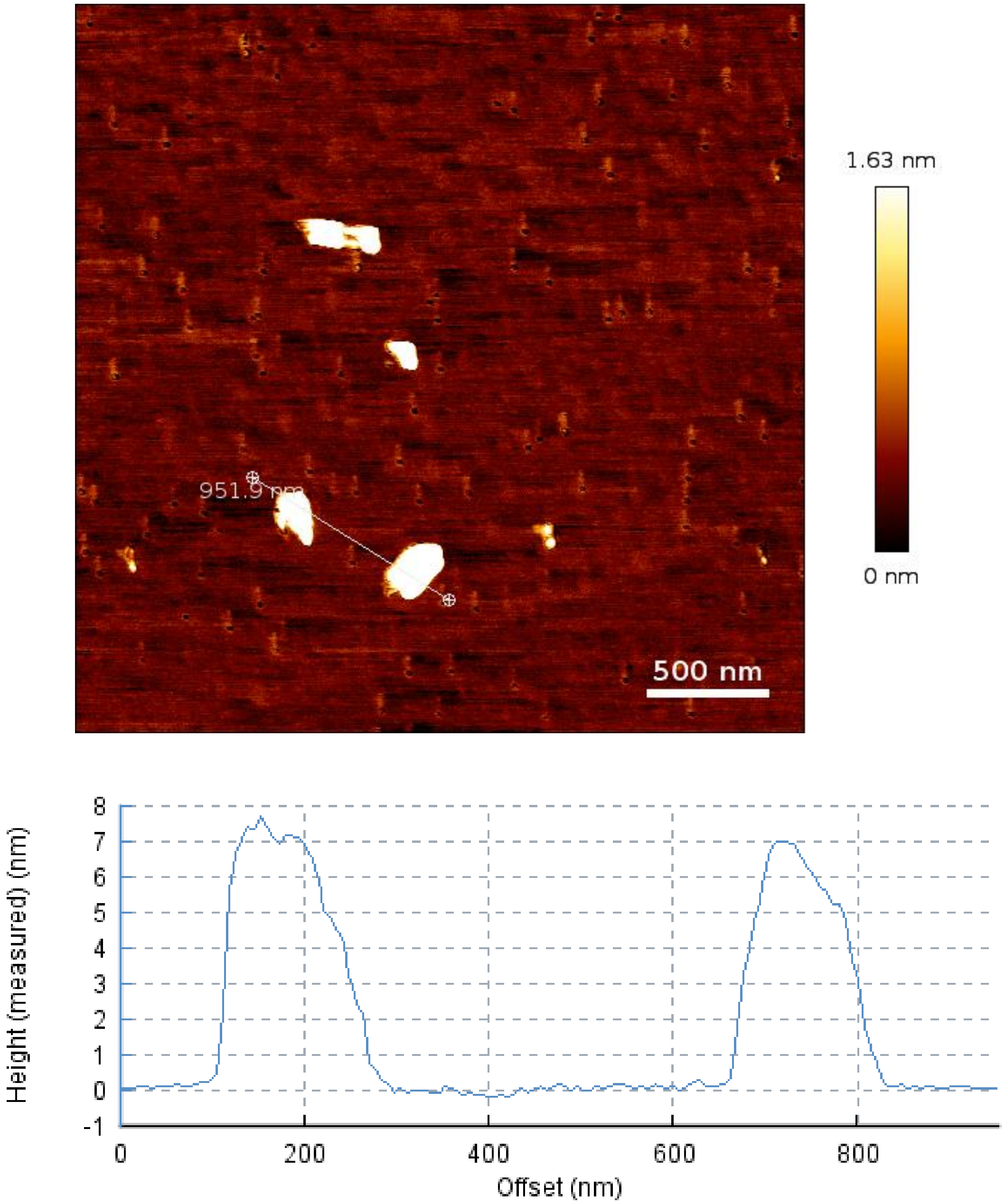

Figure S23. AFM height image of cellulose dodecamer **21** obtained after dephosphorylation of the triphosphate dodecamer **20** (2 mM), using 1600 U/µmol ALP, temperature 37 ˚C.

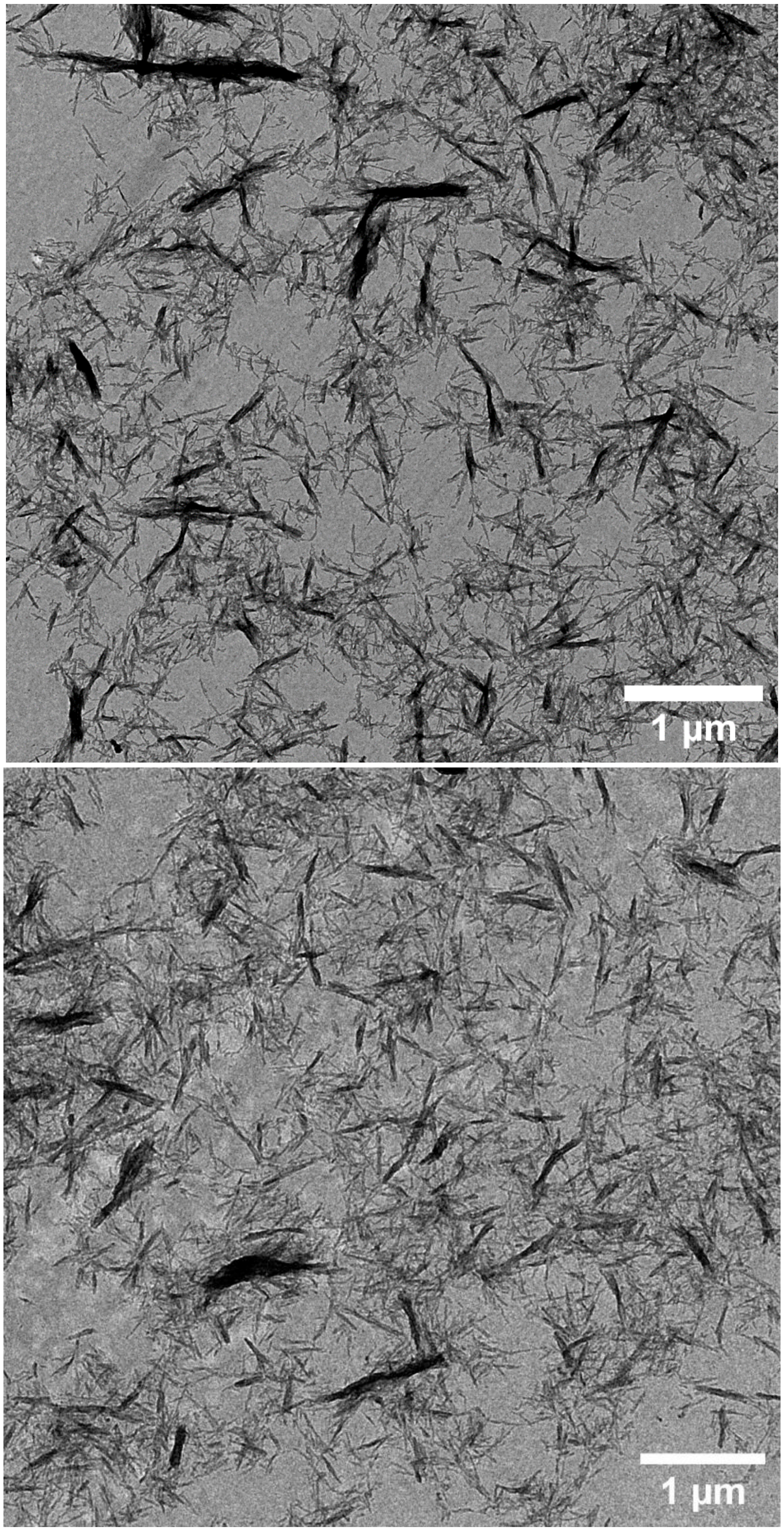

Figure S24. Representative TEM images of cellulose dodecamer **21** obtained as a precipitate after dephosphorylation of the triphosphate dodecamer (2 mM substrate, 1600 U/µmol ALP, temperature 37 °C). Sample diluted 100x and drop casted on freshly glow discharged TEM copper grid.

6.5 Platelet height comparison data

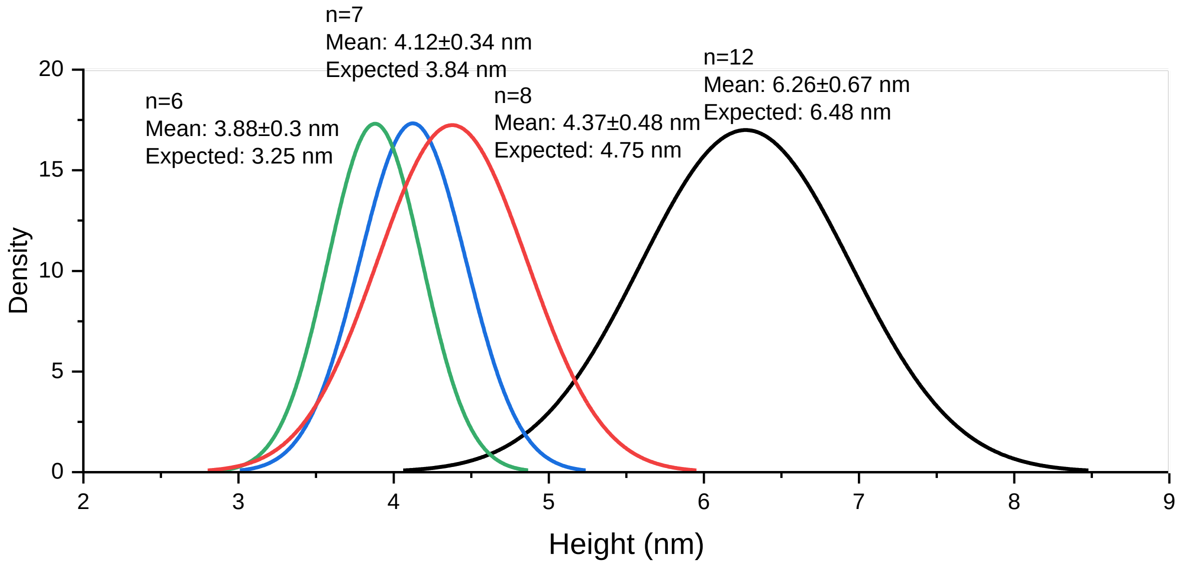

Figure S25. Comparison of average platelet height from cellulose oligosaccharides with chain lengths of 6, 7, 8 or 12 monosaccharide units.[8] Height was measured from AFM images, between 31 and 60 platelets were measured. Data can be found in Table S1.

	Chain Length			
Count	**12**	**8**	**7**	**6**
1	5.846	5.199	4.02	4.07
2	5.138	3.925	3.99	4.32
3	7.849	4.917	3.72	3.02
4	6.622	4.217	4.15	3.58
5	4.912	3.938	3.78	4.05
6	4.11	4.106	3.66	4.19
7	5.705	3.899	4.27	4.9
8	6.681	4.637	4	3.71
9	6.441	4.383	4.35	3.57
10	6.654	4.269	3.14	3.88
11	6.398	3.997	3.93	3.96
12	6.6	5.097	3.9	3.59
13	6.244	4.325	4.48	4.02
14	6.807	4.158	4.23	4.23
15	7.135	4.364	4.36	4.41
16	5.518	4.359	4.23	3.59
17	6.436	4.269	4.02	4.01
18	5.641	4.595	4.03	3.83
19	6.837	4.165	4.26	4.16
20	5.642	3.706	4.1	4.37
21	5.582	5.013	3.87	3.47
22	7.714	4.921	4.42	3.8
23	6.384	3.594	3.94	3.61
24	5.633	4.074	3.58	3.82
25	5.912	3.924	3.93	3.72
26	6.152	3.585	4.17	3.48
27	5.377	5.202	4.13	3.72
28	6.64	4.882	3.77	3.82
29	6.342	4.877	4.55	3.67
30	6.276	5.061	4.45	4.13
31	5.855	4.054	3.96	3.52
32	6.541		3.94	3.78
33	6.624		4.46	4.22
34	6.361		3.99	4.27
35	6.226		4.27	4.08
36	6.674		4.11	3.98
37	7.038		4.68	4
38	7.005		3.95	3.78
39	6.238		4.33	3.98
40	5.66		3.95	3.95
41	5.434		3.29	4.02
42	7.062		3.47	4
43	6.184		4.65	4.19
44	5.578		4.42	3.84
45	6.331		4.79	3.76
46	6.695		4.06	3.77

47	6.723	4.5	3.62
48	7.461	4.59	3.77
49	5.736	4.53	3.75
50	6.201	4.35	3.66
51	6.635	4.14	3.95
52	6.059	4.53	3.67
53	7.039	4.01	3.35
54	5.072	4.58	3.66
55	6.537	3.82	4.07
56	6.141	4.13	3.93
57	6.526		
58	7.028		
59	5.89		
60	6.383		

Table S1. Heights measured from AFM images of average platelet height from cellulose oligosaccharides with chain length of 6, 7, 8 or 12 monosaccharide units, between 31 and 60 platelets were measured.[8]

7 References

1. Bartetzko, M. P.; Schuhmacher, F.; Hahm, H. S.; Seeberger, P. H.; Pfrengle, F., *Org. Lett.* **2015,** *17*, 4344-4347.

2. Zhang, S.; Sella, M.; Sianturi, J.; Priegue, P.; Shen, D.; Seeberger, P. H., *Angew. Chem. Int. Ed.* **2021,** *60*, 14679-14692.

3. Gim, S.; Fittolani, G.; Yu, Y.; Zhu, Y.; Seeberger, P. H.; Ogawa, Y.; Delbianco, M., *Chem. - Eur. J.* **2021,** *27*, 13139-13143.

4. Dallabernardina, P.; Schuhmacher, F.; Seeberger, P. H.; Pfrengle, F., *Org. Biomol. Chem.* **2016,** *14*, 309-313.

5. Le Mai Hoang, K.; Pardo-Vargas, A.; Zhu, Y.; Yu, Y.; Loria, M.; Delbianco, M.; Seeberger, P. H., *J. Am. Chem. Soc.* **2019,** *141*, 9079-9086.

6. Gude, M.; Ryf, J.; White, P. D., *Lett. Pept. Sci.* **2002,** *9*, 203-206.

7. Eller, S.; Collot, M.; Yin, J.; Hahm, H. S.; Seeberger, P. H., *Angew. Chem. Int. Ed.* **2013,** *52*, 5858-5861.

8. Fittolani, G.; Vargová, D.; Seeberger, P. H.; Ogawa, Y.; Delbianco, M., *J. Am. Chem. Soc.* **2022,** *144*, 12469-12475.
