# Supplementary material for: Phosphates as Assisting Groups in Glycan Synthesis
Source: ACS Cent Sci. 2023 Dec 20;10(1):138–42. doi: 10.1021/acscentsci.3c00896 (PMC10823511; doi:10.1021/acscentsci.3c00896)
Supplement: Supplementary file 2 — oc3c00896_si_002.pdf [file oc3c00896_si_002.pdf]

oc-2023-008969.R1

Name: Peer Review Information for "Phosphates as assisting groups in glycan synthesis"

First Round of Reviewer Comments

Reviewer: 1

Comments to the Author

This paper describes synthesis of oligosaccharides with free phosphate groups that can regulate subsequent enzymatic synthesis and oligosaccharide solubility.

The authors installed phosphate groups on an automatic oligosaccharide synthesizer. After the deprotection of all protecting groups, they examined sialylation with bacterium sialyltransferase. In this case, the sialy-acceptor had two galactose residues, but one of galactosides had a phosphate group. The sialyl-transfer succeeded in toward non-phosphate galacoside. The authors well demonstrated the utility of the phosphate groups for the regulation of enzyme-substrate specificity. However, this concept of using protecting groups for the regulation of enzyme-substrate specificity has been widely used. Therefore this is not conceptionally new.

However, synthesis of cellulose with phosophates is very interesting. Especially 1H NMR of S5 is impressive. It is known that cellulose consisting of six beta glucose can not dissolve into water. The authors successfully synthesize octa-glucose derivatives with three phosphate groups. After that, the phosphate group could be successfully removed with phosphatase and this protocol enabled the authors to prepare nano-structure of cellulose which was analyzed by AFM.

These results and protocols will give an impact on a broad readership.

This referee strongly recommends for publication in ACS Central Science.

Before publication, the following points need to be revised.

1) Several 1H NMR spectra show unknown signals at 8.25 ppm. The authors need to give comments in their footnotes.

2) RF HPLC shows two peaks. (P31). The authors need to give comments in the footnote.

Reviewer: 2

Comments to the Author

In this article, the authors report on the use of phosphate as a transient protecting group during solid phase chemical synthesis of complex oligosaccharides. In a first strategy, a previously reported galactose building block phosphorylated at the anomeric center was introduced by automated glycan assembly into a pentasaccharide. Enzymatic sialylation resulted in the complete and selective formation of a hexasaccharide and the phosphate moiety prevented further sialylation from occurring. Treatment of the resulting hexasaccharide with a phosphatase allowed for the high yielding removal of the phosphate moiety. In a second strategy, glucose building blocks equipped with phosphate moieties were introduced into cellulose oligosaccharides to increase the solubility and prevent undesired aggregation from occurring. Overall, the authors clearly demonstrate the usefulness of temporary phosphate protecting groups for glycan synthesis and this approach is of interest for a large scientific community. The manuscript is well written, all experiments were carried out in a competent manner, and the supporting information is of good quality. Hence, publication in ACS Central Science is highly recommended, pending some revisions:

- While the introduction of phosphorylated galactose units has been shown to be useful for the site-selective sialylation of short glycans, the authors should demonstrate the general applicability of this approach. This can be done by using larger oligosaccharides or by extending the approach to other enzymatic reactions.

- The authors clearly demonstrate that the inclusion of phosphorylated glucose moieties allows for the formation of 12mer cellulose oligosaccharides which aggregate in a similar manner to natural systems upon dephosphorylation. While obtaining 12mer cellulose oligosaccharides is remarkable, the yield remains modest (1% overall yield). The authors should comment on this.

- Building block 13 in Figure 3A is modified with a dibutyl phosphate but oligosaccharides 16 and 19 are equipped with dibenzyl phosphates. The authors should fix this.

- Introduction: phosphorylation has not only been considered for peptide synthesis but also for nucleic acid synthesis (see e.g. Commun. Chem. 2022, 68, 5).

Reviewer: 3

Comments to the Author

In this work, the authors reported a new approach using phosphate ester to facilitate regio-control in enzymatic glycosylation and synthesis of aggregation prone cellulo-oligosaccharides. During deprotection, the phosphate ester would be deprotected and removed by phosphatase as a traceless protective group. The idea of using a temporary protective group that could be enzymatically removed is not necessarily new (refs 24, 33, 34). However, this is the first time phosphate esters have been adapted for this purpose beyond the more traditional anomeric leaving group or facilitating deoxygenation, which is interesting. However, major revisions are needed as outlined below.

1) I found the introduction on the analogy between the roles of phosphate in nature and the proposed usage in chemical synthesis and Figure 1 a bit misleading. In nature, phosphates play a more active role in modulating biological activities. When I read that, I had the expectation that the phosphate would be actively participating in the reactions. However, the phosphates are really more for blocking the enzyme modification of nearby sites and for improving solubilities. I don’t feel it is necessary to have the phosphates in nature panel in figure 1.

2) Figure 2A is not consistent with the synthetic steps outlined in the SI. Figure 2A showed that before Post AGA steps, the glycan was already phosphorylated. But in the SI, phosphorylation was a step in Post AGA.

3) The synthetic steps outlined in the SI are difficult to understand. For example, page 21 indicated 5 BB steps and yet on page 22, 6 BB steps were shown. As all the steps were labeled BB, it was difficult to figure out which building block was used in that particular step. I suggest the authors label them as BB1, BB2 etc and include the info on the donor used for each glycosylation to make it easier for the readers.

4) The authors suggested selective phosphorylation of the O-2 branch (arm 1). There were no characterization data supporting that. This is a major issue.

5) What were the peaks around 8.4 ppm in 1H-NMR of compounds 8, S5, 20?

6) For the cellulo-oligosaccharides, do they know the phosphate removal by phosphatase is complete? Or were there “defects” in the chain as encountered in the methylation modification approach?

Author's Response to Peer Review Comments:

MPI of Colloids and Interfaces • Am Mühlenberg 1 • 14476 Potsdam

Department of Biomolecular Systems
T +49 331 567-9301
boehme@mpikg.mpg.de
www.mpikg.mpg.de

Prof. Senior Editor,
ACS Central Science

Sept. 28th, 2023

Manuscript ID: oc-2023-008969

Title: "Phosphates as assisting groups in glycan synthesis".

Authors: Eric T. Sletten, Giulio Fittolani, Nives Hribernik, Marlene C. S. Dal Colle, Peter H. Seeberger, and Martina Delbianco.

Dear,

Thank you very much for your letter dated September 13th, 2023 informing us that you would consider the above-mentioned manuscript for publication in *ACS Central Science* after revisions.

We are including a revised manuscript that takes into account the helpful comments by all the reviewers and the editorial office. All changes in the revised manuscript are clearly marked. Below please find a detailed response to all the comments.

Reviewer #1:

1) Several 1H NMR spectra show unknown signals at 8.25 ppm. The authors need to give comments in their footnotes.

The peak at 8.25 ppm in the NMR corresponds to residual formic acid from the HPLC purification process. A footnote has been added in the SI.

2) RF HPLC shows two peaks. (P31). The authors need to give comments in the footnote.

The two peaks in the HPLC correspond to the α and β anomers at the reducing end of the glycan. A footnote has been added in the SI.

Reviewer #2:

1) While the introduction of phosphorylated galactose units has been shown to be useful for the site-selective sialylation of short glycans, the authors should demonstrate the general applicability of this approach. This can be done by using larger oligosaccharides or by extending the approach to other enzymatic reactions.

Thank you for this suggestion, we are currently expanding the scope of this approach to other classes of compounds, including new polymers and new enzymatic reactions, and this will be part of a broader follow up manuscript. For this initial communication we wanted to first showcase the proof-of-principle for the power of phosphates as traceless assisting groups. We would like to clarify that phosphorylation is performed on a backbone assembled on AGA and not introduced with a phosphorylated galactose BB. Thus, the approach could be extended for many different backbones that can be phosphorylated on resin, as demonstrated for example for cellulose (later on in the manuscript) or oligomannosides (Sletten Carb. Res. 2021).

2) The authors clearly demonstrate that the inclusion of phosphorylated glucose moieties allows for the formation of 12mer cellulose oligosaccharides which aggregate in a similar manner to natural systems upon dephosphorylation. While obtaining 12mer cellulose oligosaccharides is remarkable, the yield remains modest (1% overall yield). The authors should comment on this.

We agree that this looks like a modest yield, but it should be considered that this is over 29 independent steps, which on average would be 86 % yield per step.

3) Building block 13 in Figure 3A is modified with a dibutyl phosphate but oligosaccharides 16 and 19 are equipped with dibenzyl phosphates. The authors should fix this.

This is not an error and we apologize for the confusion. We utilize phosphates in two different ways in this is synthesis as an anomeric leaving group (BB13) and as a solubilizing group (16 and 19). Dibutylphosphate glycosyl donors (BB13) are used to construct the cellulose backbone with AGA; dibenzyl phosphate groups are introduced in a subsequent phophorylations step. To further clarify the process, we have added a full synthetic scheme in the SI and a sentence to the main text.

4) Introduction: phosphorylation has not only been considered for peptide synthesis but also for nucleic acid synthesis (see e.g. Commun. Chem. 2022, 68, 5).

This reference has been added and manuscript modified accordingly.

Reviewer #3

1) I found the introduction on the analogy between the roles of phosphate in nature and the proposed usage in chemical synthesis and Figure 1 a bit misleading. In nature, phosphates play a more active role in modulating biological activities. When I read that, I had the expectation that the phosphate would be actively participating in the reactions. However, the phosphates are really more for blocking the enzyme modification of nearby sites and for improving solubilities. I don't feel it is necessary to have the phosphates in nature panel in figure 1.

This panel has been removed from Figure 1.

2) Figure 2A is not consistent with the synthetic steps outlined in the SI. Figure 2A showed that before Post AGA steps, the glycan was already phosphorylated. But in the SI, phosphorylation was a step in Post AGA.

The graphic 2A and 3A have been altered to better reflect the SI.

3) The synthetic steps outlined in the SI are difficult to understand. For example, page 21 indicated 5 BB steps and yet on page 22, 6 BB steps were shown. As all the steps were labeled BB, it was difficult to figure out which building block was used in that particular step. I suggest the authors label them as BB1, BB2 etc and include the info on the donor used for each glycosylation to make it easier for the readers.

We apologize for that error and have made the correction on page 21. Additionally, thank you for this suggestion we have now labeled the BB#s in the synthesis table.

4) The authors suggested selective phosphorylation of the O-2 branch (arm 1). There were no characterization data supporting that. This is a major issue.

We apologize for the confusion. We have synthesized compound **5** in a way that only allows for phosphorylation at arm 1 (see scheme below, also added to the SI). Monophosphorylation was then confirmed by phosphorous NMR (SI page 24).

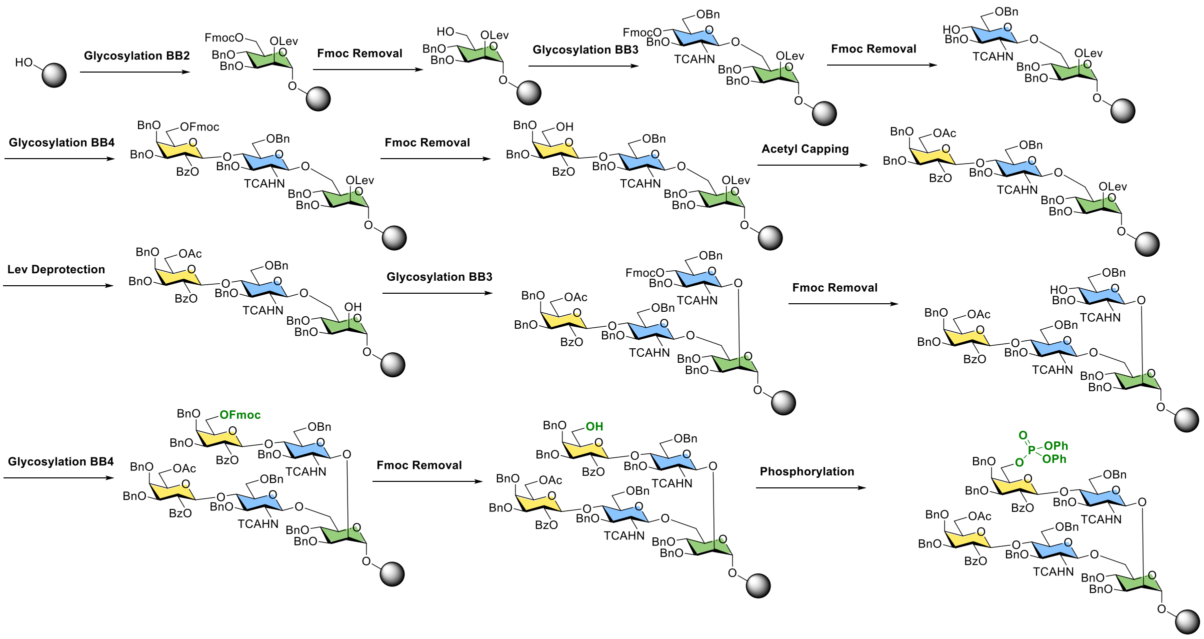

5) What were the peaks around 8.4 ppm in 1H-NMR of compounds 8, S5, 20?

The peak at 8.4 ppm in the NMR corresponds to residual formic acid from the HPLC purification process. A footnote has been added in the SI.

6) For the cellulo-oligosaccharides, do they know the phosphate removal by phosphatase is complete? Or were there “defects” in the chain as encountered in the methylation modification approach?

While we cannot rule out the presence of trace amounts of phosphorylated oligomers, we have evidence that the dephosphorylation step is nearly quantitative (See HPLC traces below, Figure S19). In Figure S19 we showed that exposure of the phosphorylated hexamer to ALP rapidly converted the substrate into the monophosphorylated hexamer (blue box, t=1 h); while cleavage of the second phosphate is slower, the target dephophorylated hexamer (yellow box, t =120 h) is obtained with a purity > 98% (based on HPLC analysis). MALDI analysis of the phosphorylated cellulose octamer (before and after

ALP treatment) also indicates complete dephosphorylation. This has also been now included into the SI as Figure S4.

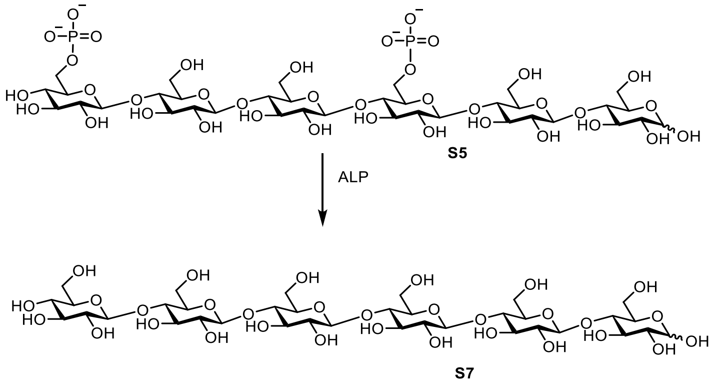

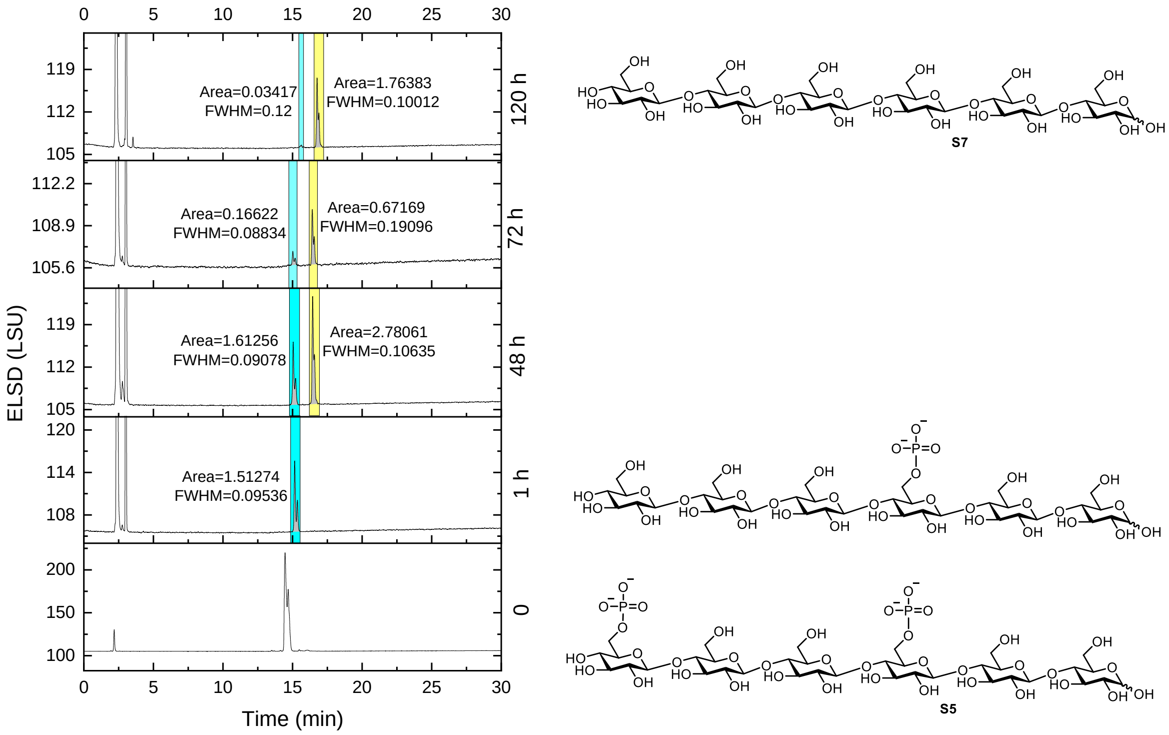

Figure S18. Representative RP-HPLC ELSD trace for monitoring the optimization for phosphate removed in **S5.**

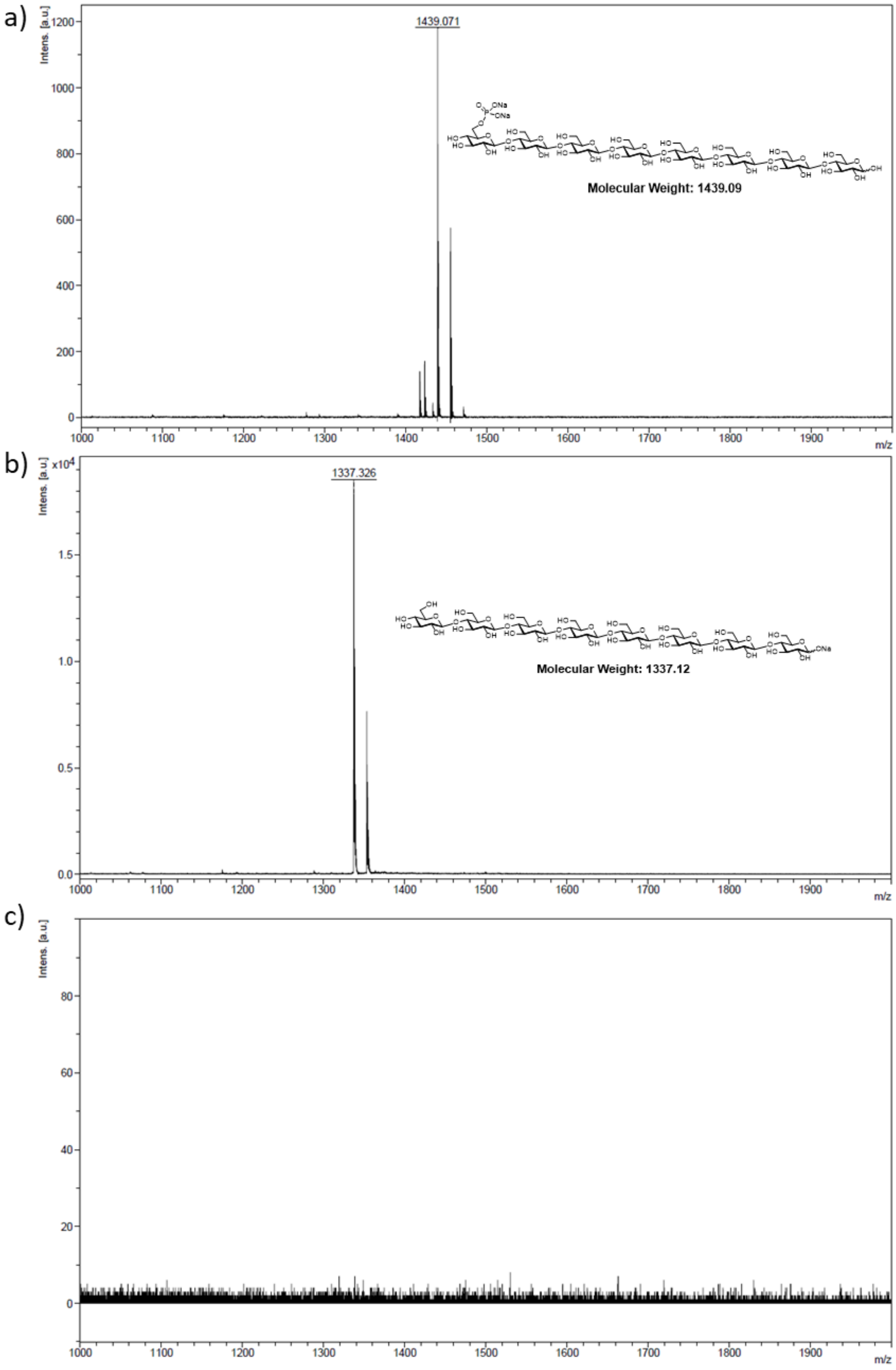

Figure S7. MALDI-ToF of cellulose octamer **17** before (a-postive mode) and after (**18**, b-positive mode, c-negative mode) exposure to ALP.

Editorial office

SI PARAGRAPH: If the manuscript is accompanied by any supporting information for publication, a brief description of the supplementary material is required in the manuscript. The appropriate format is: Supporting Information. Brief statement in non-sentence format listing the contents of the material supplied as Supporting Information.

GENERAL REF FORMATTING: Periodical references should contain authors' surnames followed by initials, article title, journal abbreviation, year, volume number, and page range. Refs with more than 10 authors should list the first 10 and then be followed by "et al."

Web sources must include access date.

TOC: Please label as "TOC Graphic"

SYNOPSIS MISSING: The synopsis should be no more than 200 characters (including spaces) and should reasonably correlate with the TOC graphic. The synopsis is intended to explain the importance of the article to a broader readership across the sciences. Please place your synopsis in the manuscript file after the TOC graphic.

SI HEADER: The supporting information should be formatted with a cover sheet listing authors, author affiliations, corresponding author email, manuscript title, and the number of pages, figures, and tables. The Author affiliations must match the MS.

SI PG#S: The supporting information pages must be numbered consecutively, starting with page S1.

The formatting needs have all been properly addressed.

Thank you for considering this manuscript for publication in *ACS Central Science.*

Sincerely yours,

Martina Delbianco & Peter H. Seeberger

Max Planck Institute of Colloids and Interfaces at Potsdam Science Park

Biomaterials
Prof. Dr. Dr.h.c. P. Fratzl

Biomolecular Systems
Prof. Dr. Dr. h.c. P. H. Seeberger

Colloid Chemistry
Prof. Dr. Dr. h.c. M. Antonietti

Sustainable and Bio-inspired Materials
Prof. S. Vignolini, Ph.D.

oc-2023-008969.R2

Name: Peer Review Information for "Phosphates as assisting groups in glycan synthesis"

Second Round of Reviewer Comments

Reviewer: 2

Comments to the Author

The authors have revised this manuscript to account for the comments of three reviewers. My original review included various comments, and the authors have now comprehensively addressed all the points that I raised and certainly markedly improved the quality of the manuscript. The response to the comments made by the other two reviewers, particularly reviewer#3, is also satisfactory. This is a very nice piece of work and publication in ACS Central Science is now recommended.

Reviewer: 1

Comments to the Author

The revision is well revised. The scientific new finding is impressive. But the authors may need to revise the footnotes according to the editorial office for the final acceptance.

Reviewer: 3

Comments to the Author

The authors have addressed prior comments satisfactorily. I recommend its publication as is.

Author's Response to Peer Review Comments:

Dear Editor,
Thank you for informing us that our revised manuscript is now suitable for publication. We have attached an updated version that includes all the changes requested by the editorial office.
Best regards
Martina and Pete
